# Orthogonal decomposition of left ventricular remodeling in myocardial infarction

**DOI:** 10.1093/gigascience/gix005

**Published:** 2017-02-06

**Authors:** Xingyu Zhang, Pau Medrano-Gracia, Bharath Ambale-Venkatesh, David A. Bluemke, Brett R Cowan, J. Paul Finn, Alan H. Kadish, Daniel C. Lee, Joao A. C. Lima, Alistair A. Young, Avan Suinesiaputra

**Affiliations:** 1 Department of Anatomy and Medical Imaging, University of Auckland, Auckland, New Zealand; 2 The Donald W. Reynolds Cardiovascular Clinical Research Center, The Johns Hopkins University, Baltimore, USA; 3 National Institute of Biomedical Imaging and Bioengineering, Bethesda, Maryland, USA; 4 Department of Radiology, UCLA, Los Angeles, USA; 5 Feinberg Cardiovascular Research Institute, Northwestern University Feinberg School of Medicine, Chicago, USA

**Keywords:** Cardiac remodeling, Magnetic resonance imaging, Shape components, Partial least squares regression

## Abstract

Left ventricular size and shape are important for quantifying cardiac remodeling in response to cardiovascular disease. Geometric *remodeling indices* have been shown to have prognostic value in predicting adverse events in the clinical literature, but these often describe interrelated shape changes. We developed a novel method for deriving orthogonal *remodeling components* directly from any (moderately independent) set of clinical remodeling indices. Results: Six clinical remodeling indices (end-diastolic volume index, sphericity, relative wall thickness, ejection fraction, apical conicity, and longitudinal shortening) were evaluated using cardiac magnetic resonance images of 300 patients with myocardial infarction, and 1991 asymptomatic subjects, obtained from the Cardiac Atlas Project. Partial least squares (PLS) regression of left ventricular shape models resulted in *remodeling components* that were optimally associated with each remodeling index. A Gram–Schmidt orthogonalization process, by which remodeling components were successively removed from the shape space in the order of shape variance explained, resulted in a set of orthonormal remodeling components. *Remodeling scores* could then be calculated that quantify the amount of each remodeling component present in each case. A one-factor PLS regression led to more decoupling between scores from the different remodeling components across the entire cohort, and zero correlation between clinical indices and subsequent scores. Conclusions: The PLS orthogonal remodeling components had similar power to describe differences between myocardial infarction patients and asymptomatic subjects as principal component analysis, but were better associated with well-understood clinical indices of cardiac remodeling. The data and analyses are available from www.cardiacatlas.org.

## Introduction

Left ventricular (LV) remodeling refers to the process by which the heart adapts its size, shape, and function in response to disease processes, or under the influence of mechanical, neurohormonal, and genetic factors [1]. Remodeling can be compensatory, for example, increased concentric hypertrophy in hypertension; or adverse, for example, increased end-systolic volume after myocardial infarction. Adverse LV remodeling characteristics after myocardial infarction provide important diagnostic and prognostic information for the therapeutic management of disease progression [2–5]. Clinical studies have identified quantitative geometric parameters (termed *clinical remodeling indices* in this paper) that describe recognized clinical patterns of remodeling with prognostic value for predicting adverse events. For example, increased LV end-diastolic volume index has been shown to be an important predictor of mortality after myocardial infarction [6]. Increased LV sphericity has also been linked with decreased survival [5]. Relative LV wall thickness [1] and apical conicity [7] are also important indices of adverse remodeling after myocardial infarction. Functional parameters such as ejection fraction (EF), which is the most common index of cardiac performance in clinical practice, are also heavily influenced by the degree of LV remodeling [8, 9]. LV longitudinal shortening is another sensitive marker of LV functional remodeling [10].

Although these clinical remodeling indices have validated prognostic value, they are often coupled so that it is difficult to separate the relative effects on heart shape. For example, end-diastolic volume is often correlated with EF in patients with myocardial infarction. It is therefore difficult to tease out the relative effects of dilatation (structural) from contraction (functional). In computational shape analysis, it is desirable to characterize the space of possible heart shapes in terms of orthogonal shape components. A shape component is a unit vector in shape space, and orthogonal components have zero dot product between different components. An orthogonal decomposition of heart shape, in which each component is related to a remodeling index with clear clinical importance, would assist clinical interpretation of the relative effects of different physiological processes underlying the development of disease. In addition, such an orthogonal decomposition would enable computational analysis of each component of remodeling present in various forms of heart disease. In particular, an orthogonal basis for shape enables robust calculation of the contribution of each component independently to the overall shape. Also, regressions using orthogonal shape components as independent variables do not suffer from the problem of multicolinearity. Thus, when analyzing the combined effects of different remodeling characteristics, it is preferred to have an orthogonal basis in a linear space.

Principal component analysis (PCA) [11] is a powerful and widely used shape analysis technique that provides an orthogonal linear shape basis. In previous work, PCA analysis of LV geometry has achieved more powerful descriptions of LV shape, and their relationships with risk factors, than traditional mass and volume analysis [12]. In a large population study, the first and second PCA LV shape components were associated with LV size and sphericity, respectively [13]. However, PCA shape components are not designed to be related to any particular clinical remodeling index, and the clinical interpretation of PCA shape components is often difficult. Previous work has shown that LV PCA shape components do not have clear clinical interpretation beyond the first two [12]. This is a common problem with PCA shape components, since they are designed to efficiently characterize shape variation without regard to possible underlying mechanisms of disease processes. Remme *et al*. [14] developed a method to decompose shape changes into modes with clear clinical interpretation. However, these modes were not orthogonal.

Decomposition of the shapes into orthogonal components enables calculation of scores as projections of each patient's shape onto the corresponding component (see Appendix). These scores quantify the amount of each shape component present in the patient's heart. One advantage of PCA shape components is that the resulting scores have zero correlation across the population (see Appendix). This is desirable in some applications; that is, if the scores can be related to underlying processes, then low correlation between scores implies that the processes have different effects within the population.

Previously, orthogonal remodeling components were generated from clinical remodeling indices using an ad hoc approach [24]. For each clinical index, a subset of cases was chosen outside two standard deviations from the mean, that is, those with very high and very low values of the clinical index. The remodeling component was then derived from these cases by fitting a line between the two groups. The problem with this method is that it relies on extremes of the distribution of the clinical index and ignores the majority of cases. This may lead to difficulties in the interpretation of the remodeling component. Therefore, the current paper sought to provide the following novel contributions: (i) calculation of remodeling components directly from regression coefficients, (ii) use of the entire distribution of the clinical index to formulate the remodeling component, and (iii) reduction of correlation among resulting remodeling component scores.

In this paper, we used partial least squares (PLS) regression to sequentially construct an orthogonal shape decomposition that is optimally related to clinical remodeling indices. Clinical remodeling indices of EDVI, sphericity, EF, relative wall thickness, conicity, and longitudinal shortening, known from the literature to have important prognostic information in the management of myocardial infarction, were used to create corresponding orthogonal shape components. By using a single PLS latent factor per clinical index, the resulting component scores were less correlated with each other and had zero correlation with those clinical indices previously removed.

## Data Description

### Patient data

LV shape models of 300 patients with myocardial infarction and 1991 asymptomatic study subjects were obtained through the Cardiac Atlas Project [15]. The cohort data have been described previously [12, 16] and are available from the Cardiac Atlas Project (http://www.cardiacatlas.org). Briefly, myocardial infarction patients (n = 300, age 31−86, mean age 63, 20% women) had clinical history of myocardial infarction with EF > 35% and infarct mass >10% of LV myocardial mass. All had stable myocardial infarction (i.e., no acute cases). Asymptomatic subjects (n = 1991, age 45−84, mean age 61, 52% women) did not have physician-diagnosed heart attack, angina, stroke, heart failure, or atrial fibrillation and had not undergone procedures related to cardiovascular disease, at the time of recruitment [12, 16].

Finite element shape models were customized to cardiac MRI exams in each case using a standardized procedure [12]. The shape models were evenly sampled on the epicardial and endocardial surfaces at sufficient resolution to capture all shape features, which resulted in 1682 Cartesian (x, y, z) points in homologous anatomical locations for each LV model.

### Clinical remodeling indices

Clinical remodeling indices included EDVI, EF, relative wall thickness, sphericity, apical conicity, and longitudinal shortening. Volumes were calculated by the summation of surface triangle volumes [17]. LV mass was calculated by subtracting endocardial from epicardial volumes multiplied by 1.05 g/ml [18]. EDVI was calculated as endocardial surface volume at end-diastole (EDV) divided by body surface area. EF was calculated as (EDV-ESV)/EDV, where ESV is the endocardial surface volume at end-systole. Relative wall thickness was defined as twice the posterior wall thickness divided by the end-diastolic diameter [19] at mid-ventricle. Sphericity was calculated as the EDV divided by the volume of a sphere with a diameter corresponding to the major axis at end-diastole in LV long axis view [20]. Apical conicity was calculated as the ratio of the apical diameter (defined as the diameter of the endocardium one-third above the apex) over the basal diameter [7] at end-diastole. Longitudinal shortening was calculated as the difference of the distance between the centroid of the most basal ring of points to the most apical point at end-systole divided by the distance at end-diastole. These indices were not intended as a comprehensive list and were limited to geometric indices (i.e., ratios that correct for size in some sense), which have either been studied for many years (e.g., relative wall thickness as a measure of concentric versus eccentric hypertrophy), or can be readily calculated from several different imaging modalities (e.g., 3D echocardiography, MRI, or CT). Attempts were made to include only indices that are moderately independent (e.g., end-systolic volume index was not included since it can be derived from end-diastolic volume index and EF).

### Remodeling components

In this paper, we use partial least squares (PLS) regression [21, 22] to explain each response variable (remodeling index) $\boldsymbol{Y} \in {\mathbb{R}^{N \times 1}}$ with a linear combination of predictor variables (LV surface points) $\boldsymbol{X} \in {\mathbb{R}^{N \times P}}$, so that

(1) \begin{equation*}\boldsymbol{Y\ } = \ \boldsymbol{X\beta '} + {\boldsymbol{E}_Y}, \end{equation*}

where $\boldsymbol{\beta }' \in {\mathbb{R}^{P \times 1}}$ is a vector of regression coefficients and ***E***_*Y*_ is the residual vector. In this paper, the dimensions *N* and *P* denote the number of cases and the number of shape features (3D surface point coordinates), respectively.

Details of the PLS regression method in comparison with principal component regression are given in the Appendix. PLS regression calculates the regression coefficients ***β***΄ as a linear combination of M latent factors, where M < P. The latent factors are chosen to maximize the covariance between response and predictor variables.

In this paper, we use centered ***Y*** and ***X*** so that the intercept is zero. We define the normalized vector of regression coefficients (ignoring the intercept term) as the “remodeling component” associated with the corresponding remodeling index ***Y***. By analogy with PCA shape components, the remodeling component is a unit length vector in shape space (column space of ***X***). We define “remodeling scores” by analogy with PCA scores, as the projection of each case onto the remodeling component:

(2) \begin{equation*}{\boldsymbol{Y}_{\boldsymbol{score}}} = \ \boldsymbol{X\beta }, \end{equation*}

where $\boldsymbol \beta $ is the normalized regression coefficients. The estimated remodelling indices can be calculated from ***Y***_*score*_  by scaling by the norm of ***β***΄ and adding the mean index.

### Orthogonal remodeling components

**Figure 1:**
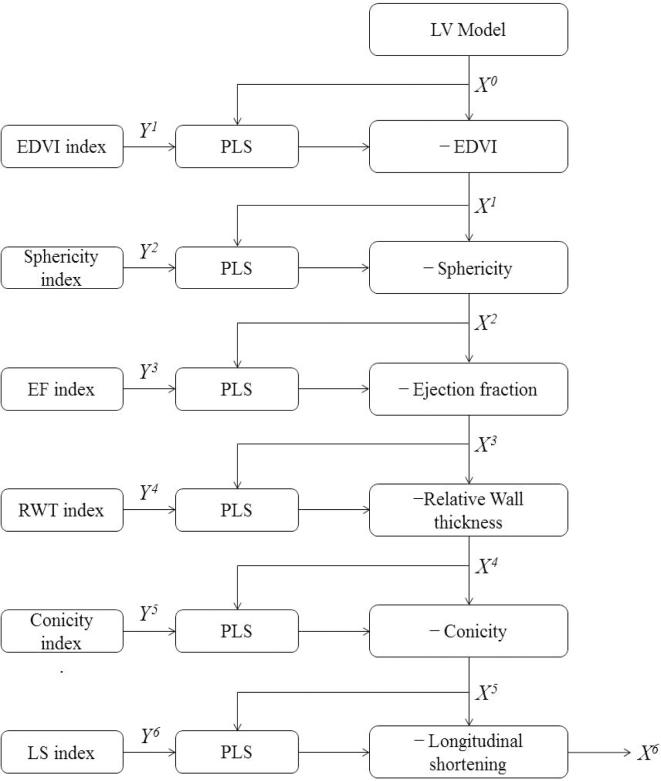
Data processing flow chart. X, shape space; Y, response variable.

Orthogonal remodeling components are calculated following the flow chart in Fig. 1. First, the remodeling index with the highest variance is chosen (EDVI). The corresponding remodeling component is calculated by PLS regression. Then a residual data matrix is generated by subtracting the projections of all cases onto the remodeling component:

(3) \begin{equation*}{\boldsymbol{X}^{\left( {{\rm{i}} + 1} \right)}} = {\boldsymbol{X}^{\rm{i}}}{\rm{\ }} - {\boldsymbol{X}^{\rm{i}}}{\boldsymbol{\beta }^{\rm{i}}}{( {{\boldsymbol{\beta }^{\rm{i}}}})^{\rm{T}}}, \end{equation*}

for i = 1,…,K, where K is the number of indices. The residual data matrix is then used in the next iteration to calculate the next remodeling component, associated with the remodeling index with the next highest variance in the data set (in this case the second index is sphericity). This process is repeated for all K = 6 remodeling indices (Fig. 1). The resulting orthonormal remodeling components [***β***^1^, ***β***^2^, …, ***β***^*K*^] form an orthogonal basis for a linear subspace of ***X***. Each ***β***^(*i* + 1)^ is orthogonal to the preceding ***β***^*i*^ because the residual data matrix ***X***^(*i* + 1)^ is orthogonal to ***β***^*i*^.

With this approach, the order of the response variables is important. We ordered the remodeling indices based on their variance in remodeling scores over the population. This is a measure of the shape variance explained by each index. The order of remodeling indices was: (1) EDVI, (2) sphericity, (3) EF, (4) relative wall thickness, (5) conicity, and (6) longitudinal shortening.

### Number of latent factors

**Figure 2:**
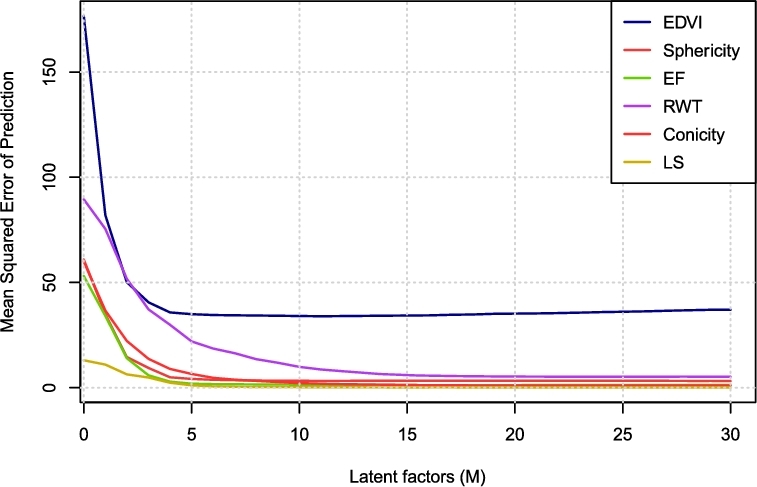
Mean squared error predictions of PLS regression coefficients using different number of latent factors (M). 10-fold cross validations were applied.

Selection of the number of latent factors *M* has a fundamental effect on the resulting remodeling components. In the current context, there is no standard method to choose the number of latent factors. In the context of prediction, cross-validation is commonly used to examine estimation error in the response variable [23]. We compared remodeling components and scores calculated from one-factor PLS (M = 1) to multi-factor PLS up to M = 30 (see Fig. 2). Standard 10-fold cross-validation was performed to test estimation error, showing that the mean squared error in estimating ***Y*** did not substantially improve after 10 latent factors. In terms of remodeling components, results for M > 10 were similar to M = 10. Experiments for 1 < M < 10 gave intermediate results. Therefore, in the following, we only compared two regression models: one-factor PLS (M = 1) and multi-factor PLS (M = 10).

### Characterization of myocardial infarction

We demonstrate the clinical applicability of our proposed shape decomposition method by examining how these clinically motivated remodeling components were associated with myocardial infarction, compared to the clinical indices themselves, or PCA shape components. Logistic regression models were used to evaluate the discriminatory power of the orthogonal remodeling components to characterize LV remodeling due to myocardial infarction. Logistic regression is a common clinical tool for examining relative effects on disease, and relative strengths of associations with disease can be quantified using odds ratios. Confounding factors (age, sex, body mass index, diastolic blood pressure, smoking status, and diabetes history) were included in each regression model as baseline variables (covariates), since they were significantly different between groups in Table 1. This was done to control for the effects of these confounding factors in each of the logistic regression models. Four logistic regression models were examined. Model 1 consisted of the baseline variables and the first 6 PCA component scores. This was used as a reference for comparison. Model 2 consisted of the baseline variables and the 6 clinical remodeling indices. Model 3 included the baseline variables and the orthogonal remodeling component scores derived from one-factor PLS. Model 4 included the baseline variables and the orthogonal remodeling component scores derived from multi-factor PLS. In each case the presence or absence of symptomatic disease was defined by the dependant variable as 1 or 0, respectively.

**Table 1:** Demographics and clinical remodeling indices for asymptomatic subjects and patients with myocardial infarction (mean ± SD). BMI, Body mass index.

Variable	Unit	Asymptomatic	MI cases	*P* value
Sex	F/M	1034/975	60/238	<0.01
Age	years	61.47 ± 10.15	62.76 ± 10.76	0.043
Height	cm	165.98 ± 9.99	173.82 ± 9.77	<0.001
Weight	kg	76.75 ± 16.50	90.06+14.14	<0.001
BMI		27.77 ± 5.09	29.73+5.57	<0.001
SBP	mmHg	126.28 ± 21.98	126.36 ± 17.50	>0.05
DBP	mmHg	71.49 ± 10.33	73.26 ± 9.82	0.006
Diabetes history	%	13.11	35.67	<0.001
Smoking status	%	12.51	11.33	>0.05
EDVI		67.83 ± 13.29	96.53 ± 25.03	<0.001
Sphericity		0.38 ± 0.08	0.41 ± 0.09	<0.001
RWT	%	39.71 ± 9.49	35.21 ± 8.38	<0.001
Conicity		0.74 ± 0.08	0.70 ± 0.08	<0.001
EF		0.63 ± 0.07	0.41 ± 0.11	<0.001
LS		0.13 ± 0.04	0.08 ± 0.03	<0.001

### Implementation

Codes were implemented in Matlab (Mathwork, Natick, MA) and R (The R Foundation, Vienna, Austria) programming languages and are available from the Cardiac Atlas Project web site^1^. The Matlab implementation requires the plsregress function from the Statistics and Machine Learning Toolbox. The R implementation requires the pls package [25]. We used SIMPLS algorithm [22] to compute the PLS regression in both versions due to its fast calculation. We compared the PLS regression coefficients using different methods provided by the pls package from R, that is, kernel, wide kernel, and classical orthogonal scores algorithms, and the results were very similar in the regression coefficients obtained.

### Statistical analyses

Root mean square (RMS) errors in the angle between remodeling component unit vectors were used to quantify the differences arising from different training data sets: (1) asymptomatic cases from 100 to 1900, versus all asymptomatic cases, and (2) balanced data set (300 asymptomatic and 300 myocardial infarction) versus the full data set (1991 asymptomatic and 300 myocardial infarction).

For the logistic regression, the independent variables (components and baseline variables) were included simultaneously and the models were computed using SAS. A *P* value of <0.05 was considered significant. Four commonly used measures were used to quantify the goodness-of-fit of the regression models: deviance, Akaike information criterion (AIC), Bayesian information criterion (BIC), and the area under the receiver operating characteristic curve (AUC) [12]. Smaller deviance, AIC, and BIC, and larger AUC are indicative of better goodness-of-fit. Statistical tests to determine whether the AUC of a model is significantly greater or less than another model were performed using one-sided paired nonparametric tests for AUC values [26], implemented in the pROC package [27]. A *P* value < 0.05 was considered as statistically higher or smaller AUC value.

## Results

Unless otherwise stated, all experiments were performed including all cases (asymptomatic and MI patients). Participant characteristics are summarized in Table 1. Some demographic characteristics were significantly different between the asymptomatic subjects and the myocardial infarction cases, including gender ratio, age, height, weight, blood pressure, and diabetes history. Clinical LV remodeling indices were also significantly different, as expected. The myocardial infarction patients had larger LV EDVI, increased sphericity, thicker walls, less conicity, smaller EF, and reduced longitudinal shortening than the asymptomatic subjects.

**Figure 3:**
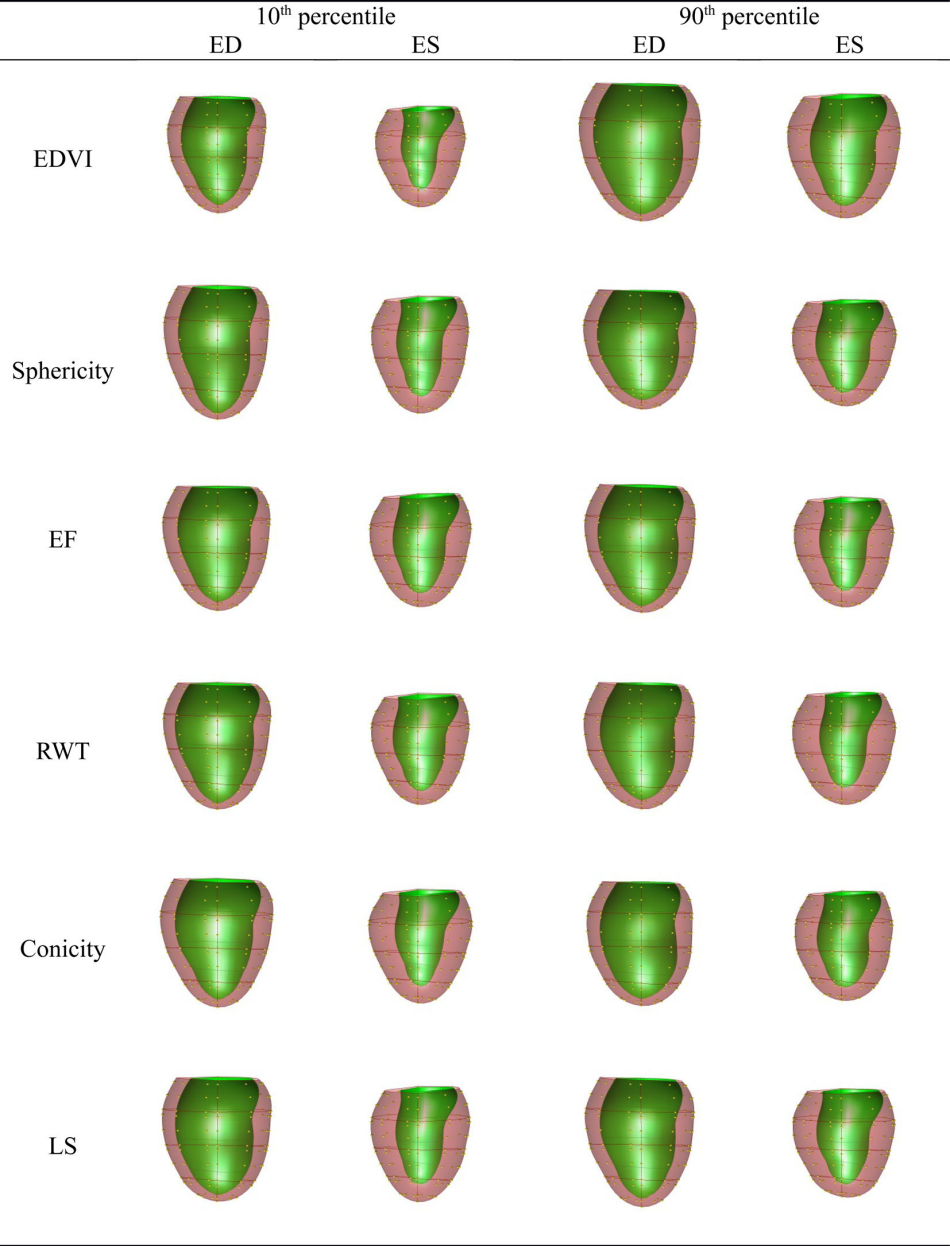
Plot of the PLS clinical components (*M* = 1). Viewpoint is from the posterior with the septum on the left. ED, end-diastole; ES, end-systole. Full animations of each clinical component are shown http://www.cardiacatlas.org/tools/lv-shape-orthogonal-clinical-modes.

**Figure 4:**
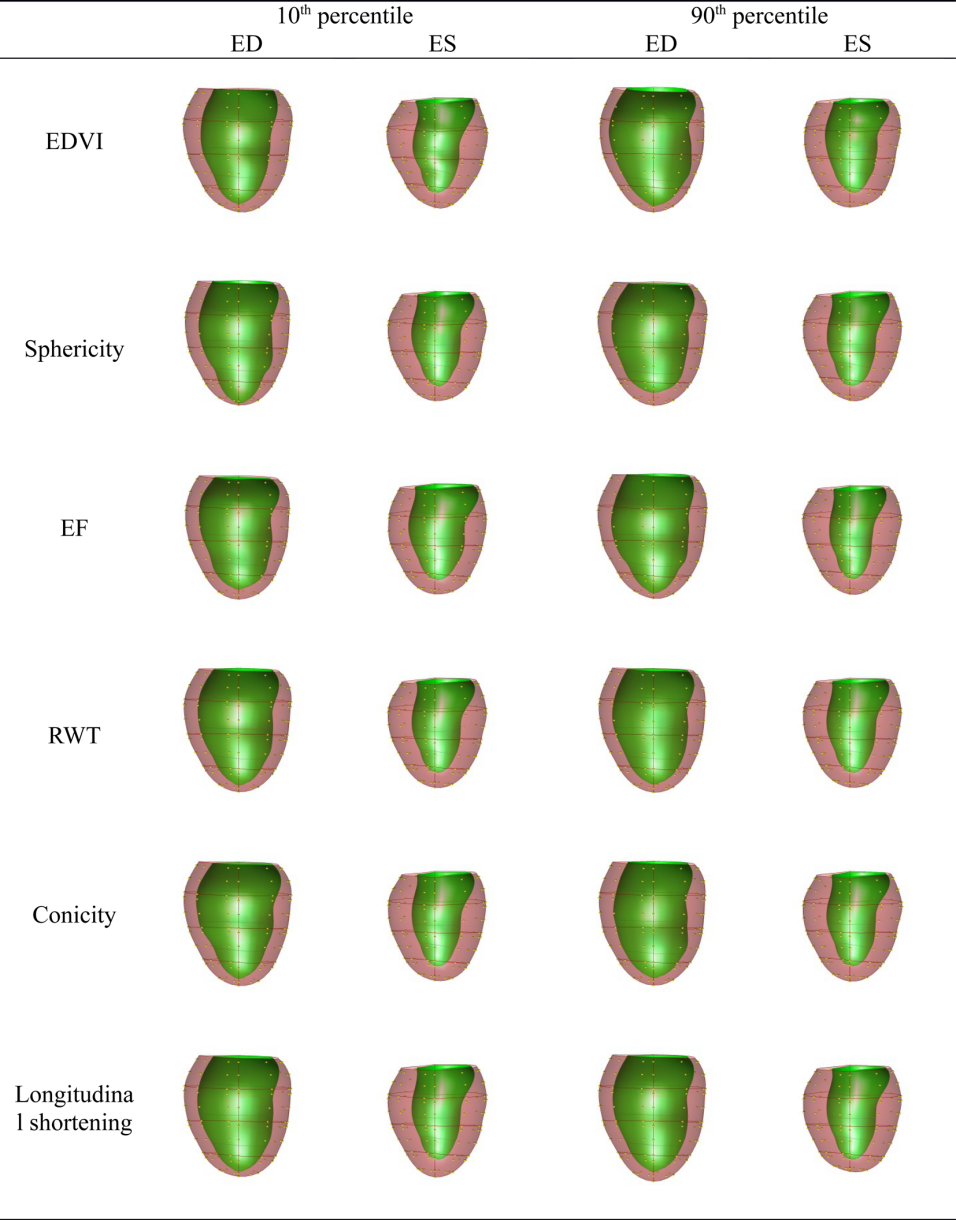
Plot of the PLS clinical components (*M* = 10). Viewpoint is from the posterior with the septum on the left. ED = end-systole; ES = end-diastole.

The orthogonal PLS components corresponding to EDVI, sphericity, EF, relative wall thickness, conicity, and longitudinal shortening are visualized in Fig. 3 (M = 1) and Fig. 4 (M = 10). These visualizations are useful in understanding the effect of each component on shape.

Linear correlation coefficients (Pearson) were calculated between the clinical indices and the component scores in the combined population. Correlation coefficients between PLS remodeling scores and clinical indices are reported in Table 2 for M = 1 and in Table 3 for M = 10. A single latent factor resulted in zero correlation between the remodeling scores and the indices corresponding to all the components previously removed in the Gram-Schmidt procedure (Table 2). Using more latent factors resulted in better correlation between each remodeling score and its corresponding index (diagonal elements are higher in Table 3 than in Table 2). Correlation coefficients between clinical indices and scores of the first 6 PCA components of the original dataset are shown in Table 4 for comparison.

**Table 2:** Correlation coefficients between the clinical indices and the PLS remodeling component scores (M = 1).

	EDVI score	Sphericity score	EF score	RWT score	Conicity score	LS score
EDVI	0.82	0	0	0	0	0
Sphericity	0.03	0.83	0	0	0	0
EF	−0.75	0.03	0.61	0	0	0
RWT	−0.20	−0.16	−0.04	0.53	0	0
Conicity	−0.14	−0.28	0.30	0.21	0.72	0
LS	−0.45	0.03	0.61	−0.17	0.20	0.53

**Table 3:** Correlation coefficients between the clinical indices and the PLS remodeling component scores (M = 10).

	EDVI score	Sphericity score	EF score	RWT score	Conicity score	LS score
EDVI	0.94	0.27	−0.34	−0.64	−0.13	−0.31
Sphericity	0.30	0.97	−0.15	−0.16	−0.25	−0.13
EF	−0.41	−0.28	0.90	0.22	0.25	−0.02
RWT	−0.65	−0.12	0.26	0.99	0.25	0.53
Conicity	−0.13	−0.22	0.38	0.25	0.97	0.24
LS	−0.32	−0.13	0.02	0.56	0.25	0.98

**Table 4:** Correlation coefficients between the clinical indices and the first six PCA shape components.

	PC 1	PC 2	PC 3	PC 4	PC 5	PC 6
EDVI	0.80	−0.01	−0.74	−0.18	−0.13	−0.45
Sphericity	−0.26	−0.80	0.19	0.19	0.30	0.06
EF	−0.01	0.09	−0.11	0.03	−0.09	−0.20
RWT	0.10	0.24	−0.21	−0.25	−0.25	−0.18
Conicity	0.10	0.13	−0.15	−0.11	−0.15	−0.14
LS	0.21	0.02	0.03	−0.15	0.50	0.37

The correlation coefficients among the clinical indices are shown in Table 5. These show strong correlations between several clinical indices. The decreasing diagonal correlations in Tables 2 and 3 are likely due to this interdependence between clinical indices. Thus, RWT and LS are related to indices previously removed by the orthogonalization process (RWT is related to EDVI and sphericity, LS is related to EF, etc.).

**Table 5:** Correlation coefficients among the clinical indices.

	EDVI	Sphericity	EF	RWT	Conicity	LS
EDVI	1	0.28	−0.60	−0.37	−0.11	−0.29
Sphericity	0.28	1	−0.11	−0.28	−0.22	−0.13
EF	−0.60	−0.11	1	0.18	0.26	0.57
RWT	−0.37	−0.28	0.18	1	0.32	0.00
Conicity	−0.11	−0.22	0.26	0.32	1	0.26
LS	−0.29	−0.13	0.57	0.00	0.26	1

Correlations between the PLS remodeling scores are shown in Table 6 for M = 1 and in Table 7 for M = 10. The minimum correlation between remodeling scores was achieved with M = 1 (Table 6).

**Table 6:** Correlation coefficients among the PLS remodeling scores (M = 1).

	EDVI score	Sphericity score	EF score	RWT score	Conicity score	LS score
EDVI score	1	−0.29	−0.15	0.22	−0.15	−0.08
Sphericity score	−0.29	1	0.001	−0.04	0.01	0.22
EF score	−0.15	0.001	1	0.09	0.09	0.47
RWT score	0.22	−0.04	0.09	1	−0.08	0.002
Conicity score	−0.15	0.01	0.09	−0.08	1	0.16
LS score	−0.08	0.22	0.47	0.002	0.16	1

**Table 7:** Correlation coefficients among the PLS remodeling scores (M = 10).

	EDVI score	Sphericity score	EF score	RWT score	Conicity score	LS score
EDVI score	1	0.29	−0.68	−0.37	−0.15	−0.34
Sphericity score	0.29	1	−0.17	−0.15	−0.25	−0.14
EF score	−0.68	−0.17	1	0.27	0.25	0.53
RWT score	−0.37	−0.15	0.27	1	0.31	−0.01
Conicity score	−0.15	−0.25	0.25	0.31	1	0.24
LS score	−0.34	−0.14	0.53	−0.01	0.24	1

**Figure 5:**
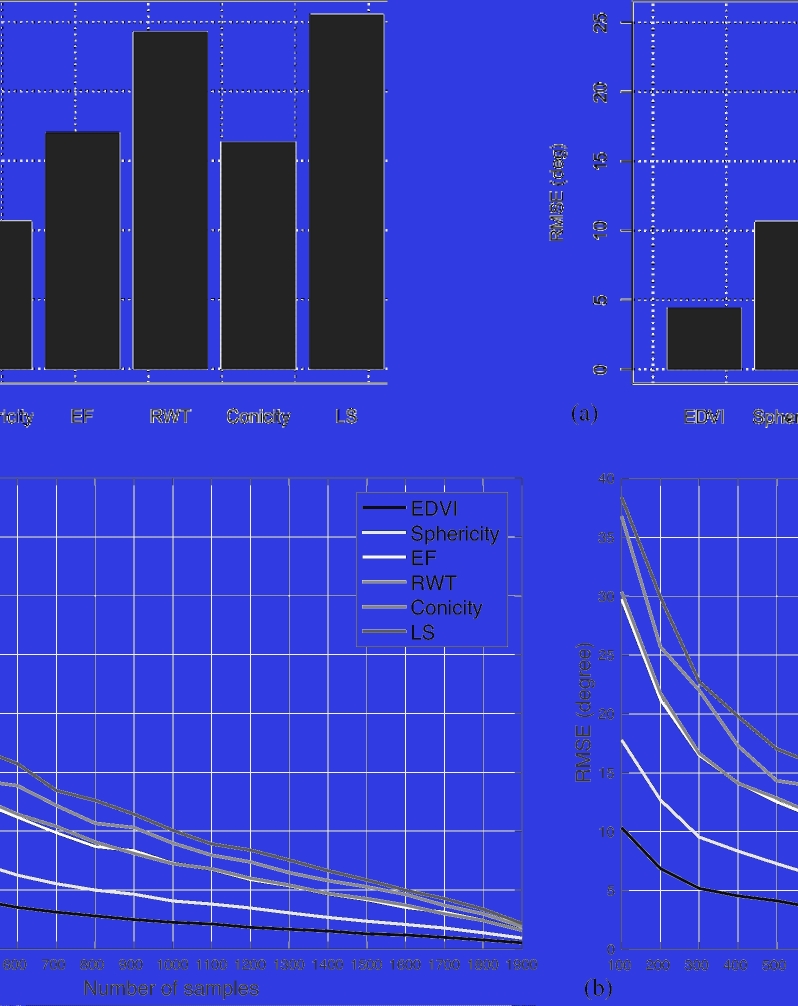
Root mean squared error (RMSE) in terms of angle differences between remodeling components. (**a**) Root mean squared errors between randomly sampled balanced data sets (300 ASYMP and 300 MI) and full data set (1991 ASYMP and 300 MI). Average of 50 trials. (**b**) Root mean squared errors varying number of asymptomatic subjects compared with the full data set (1991 samples). Average of 50 trials.

A series of experiments was performed to compare remodeling components between the full data set (1991 asymptomatic + 300 myocardial infarction) with symmetric datasets, that is, 300 asymptomatic and 300 MI patients) with 50 trials of randomly selected asymptomatic subsets. In this case, similar remodeling components are reflected by the same unit ***β*** vectors, which can be measured by angle differences (derived from the dot product) between two ***β*** vectors. Fig. 5a shows the root mean square errors of ***β*** vector differences between the subset and the full models. Only the first component (EDVI) showed <5 degrees difference, but increasing differences in other components were observed. This was expected since the characteristics of the cases included in the training set have an influence on the results.

Considering only the asymptomatic cases, we investigated the differences in the remodeling components with different number of samples. Fig. 5b shows the RMS errors of randomly sampled cases (50 trials each) with respect to the full 1991 cases. At least 1100 cases were needed to get below 10 degrees difference with the full cohort in all components.

The results of logistic regression models to characterize remodeling associated with myocardial infarction using the orthogonal remodeling scores are shown in Table 8. For the one-factor PLS remodeling scores, the odds ratio of EDVI, sphericity, EF, wall thickness, and conicity indicate that myocardial infarction patients tend to have larger and more spherical LV shapes with thinner walls and a less conical shape. The multi-factor PLS remodeling scores showed somewhat different results, with EDVI, EF, conicity, and longitudinal shortening scores being significant. This may be due to the increased multi-colinearity between remodeling scores in the multi-factor case.

**Table 8:** Four logistic regressions for myocardial infarction.

		Standard		Standardized	Odds	OR 95 % Confidence	
Variable	Coefficient	error	*P* value	coefficient	ratio (OR)	Interval	
Model 1: PCA shape components + baseline variables							
**PC 1**	**2.644**	**0.177**	**<.0001**	**1.455**	**14.066**	**9.942**	**19.901**
**PC 2**	**−0.605**	**0.102**	**<.0001**	**−0.334**	**0.546**	**0.447**	**0.666**
PC 3	0.071	0.112	0.524	0.039	1.074	0.863	1.336
**PC 4**	**2.031**	**0.153**	**<.0001**	**1.111**	**7.625**	**5.652**	**10.287**
**PC 5**	**0.391**	**0.106**	**<.0001**	**0.215**	**1.478**	**1.200**	**1.821**
PC 6	−0.113	0.119	0.342	−0.062	0.893	0.708	1.127
Model 2: Clinical indices + baseline variables							
**EDVI**	**0.041**	**0.008**	**<.0001**	**0.412**	**1.042**	**1.027**	**1.058**
Sphericity	0.002	0.014	0.870	0.010	1.002	0.975	1.030
**EF**	**−0.164**	**0.015**	**<.0001**	**−0.966**	**0.849**	**0.825**	**0.874**
RWT	0.002	0.014	0.875	0.012	1.002	0.975	1.030
**Conicity**	**−0.037**	**0.016**	**0.018**	**−0.161**	**0.963**	**0.934**	**0.994**
**LS**	**−0.148**	**0.037**	**<.0001**	**−0.325**	**0.862**	**0.802**	**0.927**
Model 3: PLS remodeling scores (M = 1) + baseline variables							
**EDVI score**	**2.859**	**0.191**	**<.0001**	**1.574**	**17.444**	**11.997**	**25.365**
**Sphericity score**	**0.895**	**0.125**	**<.0001**	**0.492**	**2.446**	**1.915**	**3.124**
**EF score**	**−1.540**	**0.148**	**<.0001**	**−0.846**	**0.214**	**0.160**	**0.287**
**RWT score**	**−1.289**	**0.146**	**<.0001**	**−0.710**	**0.275**	**0.207**	**0.367**
**Conicity score**	**0.331**	**0.124**	**0.007**	**0.181**	**1.392**	**1.093**	**1.774**
LS score	−0.041	0.140	0.769	−0.023	0.960	0.729	1.263
Model 4: PLS remodeling scores (M = 10) + baseline variables							
**EDVI score**	**0.823**	**0.161**	**<.0001**	**0.454**	**2.277**	**1.661**	**3.120**
Sphericity score	−0.189	0.114	0.098	−0.103	0.828	0.662	1.036
**EF score**	**−1.843**	**0.180**	**<.0001**	**−1.016**	**0.158**	**0.111**	**0.225**
RWT score	0.087	0.128	0.495	0.048	1.091	0.849	1.403
**Conicity score**	**−0.393**	**0.122**	**0.001**	**−0.216**	**0.675**	**0.531**	**0.858**
**LS score**	**−0.665**	**0.141**	**<.0001**	**−0.365**	**0.514**	**0.390**	**0.678**

All the models are adjusted for age, gender, BMI, DBP, smoking status, and diabetes history. Bold rows indicate *P* < 0.05.

**Figure 6:**
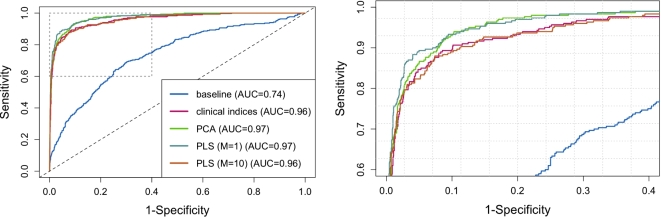
ROC curves for the five logistic regression models. The right figure shows a zoomed-in view to demonstrate the differences between the four models. ROC, receiver operating curve.

Table 9 shows the comparisons of the regression models. All four regression models showed significant improvement compared with the baseline variables alone. The logistic regression based on one-factor PLS orthogonal remodeling scores showed the best deviance, AIC and BIC, and AUC. The AUC (Fig. 6) for the one-factor remodeling scores was significantly greater than the multi-factor remodeling scores and the original clinical indices, but was not significantly different from the PCA model.

**Table 9:** Comparison of the four logistic regression models. Smaller deviance, AIC and BIC, and larger AUC are indicative of better goodness-of-fit. Bold row indicates best performance

	Deviance	AIC	BIC	AUC
Baseline	1560	1574	1615	0.7415
Indices	710	727	802	0.9594
PCA scores	607	633	708	0.9725
**PLS scores (M = 1)**	**569**	**595**	**669**	**0.9739**
PLS scores (M = 10)	683	709	784	0.9598

**Figure 7:**
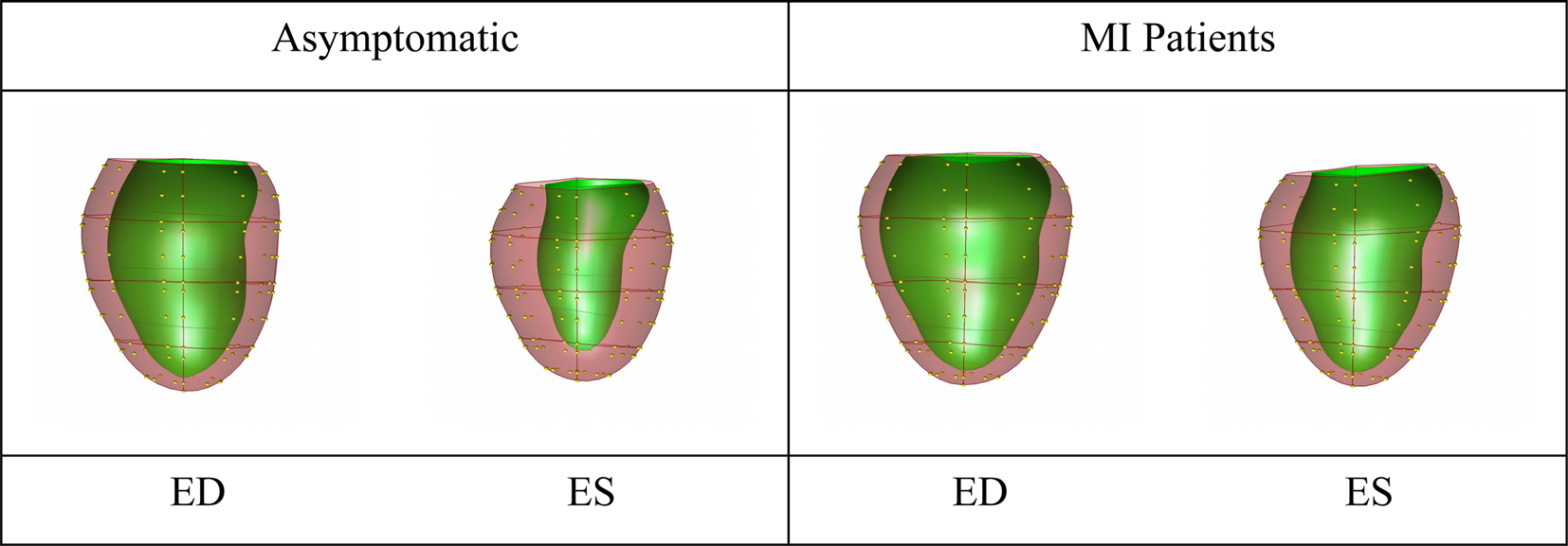
Visualization of shape changes between asymptomatic volunteers and MI patients, using the combined PLS (*M* = 1) components. Viewpoint is from the posterior with the septum on the left. Plots show the shapes associated with the median logistic regression score for the asymptomatic and MI patient groups respectively. MI patients show larger ventricles, less ejection, and thinner walls. ED, end-diastole; ES, end-systole

The standardized coefficients of the logistic regression model were used to create a linear combination of the PLS (M = 1) components generating a combined remodeling score, called the logistic regression score, separating the two groups. The F logistic regression scores (Model 3) for all cases were calculated, and the median shapes were calculated by projecting the coefficients of the PLS components estimated in the logistic regression model back on the population shape space. These are plotted in Fig. 7. This graphically shows the shape changes that best distinguish the two groups with baseline variables adjusted, showing that LV remodeling due to myocardial infarction is associated with larger volume, more spherical shape, and thinner wall thickness. Since the logistic regression coefficients refer to contributions from remodeling components, the amount of each remodeling component contributing to the logistic regression score could be quantified. This gives an intuitive explanation of the logistic regression score in terms of remodeling components associated with clinical remodeling indices.

## Discussion

Patients with myocardial infarction exhibit significant shape changes with respect to the normal population due to cardiac remodeling. An atlas-based analysis of cardiac remodeling has previously shown better characterization of remodeling due to myocardial infarction than traditional mass and volume analysis in large data sets [12]. The framework consisted of three steps: (1) fitting a finite element model to the LV MR images, (2) shape component extraction from the aligned shapes, and (3) quantification of the association between the components and disease using logistic regression. Although PCA provides orthogonal shape components, which describe the maximum amount of variation for the fewest number of components, these components typically do not correspond with clinical indices of cardiac remodeling. To avoid this problem and give the components a clear clinical interpretation, while maintaining the advantages of orthogonality, we developed a method to generate orthogonal shape components from any set of clinical indices using PLS.

In this paper, we generated a linear orthogonal shape basis from the full finite element shape parameters. Clinical indices, such as EDVI, sphericity, EF, relative wall thickness, conicity, and longitudinal shortening, were derived from the finite element shape model. Similar to PCA, the shape components derived from PLS regression are orthogonal. In PCA, the resulting component scores also have zero correlation across the population cohort, but this is not the case with PLS. Table 7 shows that PLS component scores with M = 10 were significantly correlated, similar to the original clinical indices in Table 5. This is expected since M = 10 results in strong correlations between scores and indices (Table 3). PLS components using both M = 10 and M = 1 obtain effective shape representation for each clinical index, as evidenced by the correlation coefficients with the clinical indices (diagonal terms in Tables 2 and 3), compared to the first six components of PCA (Table 4).

We found that the correlations between the scores of different indices for PLS with M = 1 become smaller than the original indices and scores of PLS with M = 10. For example, the correlation between EDVI and EF was originally −0.60 (Table 5), then became −0.68 from PLS with M = 10 (Table 7); however it was −0.15 from PLS with M = 1 (Table 6). Not only did a single latent factor result in the least correlation between component scores (Table 6), but it also resulted in zero correlation between component scores and previously removed indices (upper triangle of Table 2). This result is a feature of one-factor PLS applied in this context. One-factor PLS computes a single latent factor that maximizes the cross-correlation between ***X*** and ***Y***. The resulting remodeling component is a vector in the same direction as this single latent factor (in fact ***β***∝***X***^*T*^***Y***). Subtracting this component from the shape space leads to zero correlation between the residual shapes and ***Y***. For multi-factor PLS, the resulting remodeling component is a combination of all the latent factors and no longer has this property.

These orthogonal components derived from traditional remodeling indices may be used to partition shape into contributions from each component, independent of the others. Correlation analysis shows that these clinically derived components have high correspondence with traditional remodeling indices (diagonals in Tables 2 and 3), either virtually following the clinical indices’ original correlation (Table 5) in M = 10 (Table 3) or by sacrificing some of the diagonal correlations in exchange for decoupling with previous indices in M = 1 (Table 2). Remodeling scores at M = 10 are more correlated with the original clinical indices than M = 1 but at the expense of their ability to explain variance in the original shape space. It can therefore be argued that M = 10 generates more ‘specific’ shapes with lesser representative power.

Previous studies have also used PLS to derive information on cardiac remodeling [28]. Lekadir *et al*. [28] used PLS to characterize myocardial infarction using class labels as the response variable and the data matrix as the predictor variables. They found that running the regression with a range of latent factors and combining the estimations with a median operator could obtain better performance. In the current paper, logistic regression was used (instead of PLS in [28]) with the class labels as the response variable, because this is a commonly used clinical tool to examine associations with disease, and it is simple to calculate relative effects of the components on the response variable as odds ratios. The current paper also differs from [28] in the use of PLS to derive orthogonal remodeling components and the finding that a single latent factor reduces correlations in the resulting remodeling scores.

The results also show that clinically derived components quantitatively characterize remodeling associated with myocardial infarction with similar power as PCA components. Three logistic regression models based on the clinical indices, PCA components, and orthogonal remodeling components derived from clinical indices were all similar in terms of goodness of fit. Significance tests on areas under the ROC curves (AUC) revealed that the one-factor PLS model showed significantly greater AUC compared with the multi-factor PLS model and the clinical indices model, but not significantly different from the PCA model. Hence the single latent factor remodeling components characterized myocardial infarction similarly to PCA, while having the added advantage of having clear clinical interpretation with respect to their corresponding clinical indices, as well as being an orthogonal decomposition of shape space.

Coefficients of the remodeling components estimated in the logistic regression model were projected back on the population shape space. Fig. 7 visualizes the shape changes characterizing presence of disease. This combined component can be used for tracking individual patients over time in future studies, by quantifying the degree to which their LV shapes compare with the remodeling spectrum.

In this study, we included all of the available cases (1991 asymptomatic and 300 myocardial infarction), since we were primarily interested in the proof of concept. Having a balanced data set is preferable to enable the analysis of differences between “asymptomatic remodeling” and “symptomatic remodeling”, which would be of considerable interest in terms of physiological driving factors. However, Fig. 5b indicates that over 1000 cases would be required for robust identification of remodeling components. Also, physiological functions between different pathological groups can be quite different. For example, comparing the remodeling components of 1991 asymptomatic subjects only with remodeling components of 1991 asymptomatic + 300 myocardial infarction revealed differences of 9.1 degrees in EDVI, 6.4 degrees in sphericity, 15.1 degrees in EF, 7.0 degrees in RWT, 9.5 degrees in conicity, and 8.4 degrees in longitudinal shortening. Hence, the myocardial infarction patients, which were only 24 % from all samples, had a significant influence on all the remodeling components.

Supervised dimension reduction techniques such as information maximizing component analysis and linear discriminate analysis have also been used to extract a single remodeling component that can best characterize myocardial infarction using surface sampling [29]. In the current study, the shape components of each clinical index were obtained first and then combined using logistic regression. The shape changes due to myocardial infarction obtained by this logistic regression model can therefore be more easily explained as a combination of well-understood shape components through the logistic regression coefficients.

This method can be applied to any set of (moderately independent) clinical measures, enabling visualization and quantification of the corresponding shape components, thereby further exploiting shape information in a clinically meaningful fashion.

## Limitations

The cross-sectional nature of these data limits the understanding that can be gained on the physiological factors underlying remodeling processes. However, the methods developed in this work can be applied to future studies to track patients over time, or to epidemiological studies such as the Multi-Ethnic Study of Atherosclerosis [30] and the UK Biobank [31]. We also limited the clinical remodeling indices examined in this paper to those geometric indices that have been well established in the clinical literature. These indices are also readily available from several imaging modalities such as 3D echo and CT. The order the indices are included in the basis has an effect on the resulting remodeling components. While we used the variance of the corresponding remodeling scores (a measure of shape variance explained), other methods are possible and this requires further research. Finally, we did not include structural information on the location and size of the infarct. While more information is becoming available on the interesting effects of infarct size and transmurality, this is left for future work. Also, many patients have comorbidities such as valvular disease, which was not examined in the current study.

## Potential implications

An orthogonal decomposition of shape in relation to remodeling indices of known prognostic value will enable multi-dimensional characterization of the ways in which the heart adapts with the progression of disease, for example, after myocardial infarction. The remodeling components were able to characterize disease as well as standard methods, with the added advantages of having clear clinical interpretation with respect to their corresponding clinical indices, as well as being an orthogonal decomposition of shape space. The resulting remodeling scores can be used to track the progression of remodeling over time, against reference populations. This would enable automatic computation of z-scores giving precise information on how the patient's heart compares against the reference population. Although the remodeling components were generated from a largely asymptomatic population in this work, we showed how they describe the shape changes undergone in myocardial infarction relatively well. We also showed how the amount of each remodeling component could be quantified in association with the presence of clinical disease, highlighting significant contributions of ventricular size, sphericity, and relative wall thickness. These methods enable new knowledge to be derived from medical imaging examinations on the underlying mechanisms driving the adaptation of the heart in response to disease. Future work can also examine how the remodeling scores are related to future adverse events, for example, using clinical outcomes.

## Availability of supporting data and materials

All data and results are available from www.cardiacatlas.org. The data are not publicly available due to IRB restrictions on the contributing studies; however, data are made available on approval of a research application submitted under the Cardiac Atlas Project data sharing policy (www.cardiacatlas.org). Data further supporting this work are available in the *GigaScience* repository, GigaDB [32].

## Declarations

### Abbreviations and acronyms

DBP: Diastolic blood pressure; EDVI: End-diastolic Volume Index; EF: Ejection fraction; PCA: Principal component analysis; PLS: Partial least squares; LS: Longitudinal shortening; LV: Left ventricular; MI: Myocardial infarction; RWT: Relative wall thickness; SBP: Systolic blood pressure

### Ethics approval and consent to participate

This study was approved by the local institutional review boards (Johns Hopkins University School of Medicine NA_00031350; Northwestern University CR1_STU00000078; New Zealand Multi-region Ethics Committee MEC/08/04/052) and all participants gave written informed consent.

### Consent for publication

Not applicable.

### Competing interests

The authors declare that they have no competing interests.

### Funding

This project was supported by award numbers R01HL087773 and R01HL121754 from the National Heart, Lung, and Blood Institute. David A. Bluemke is supported by the NIH intramural research program. Xingyu Zhang was supported by the China Scholarship Council.

### Authors΄ contributions

All authors were involved in the design of the study, interpretation of the data, drafting and revision of the manuscript, and final approval of the submitted manuscript. XZ, PM-G, and AS performed the statistical analyses.

### Authors’ information

XZ is a biostatistician. PM-G is a biostatistician and expert in bioinformatics. BA-V is a bioengineer and expert in medical image analysis. DB is a radiologist and Director of Radiology and Imaging Sciences at the National Institute of Biomedical Imaging and Bioengineering. BR is a clinical engineer and an expert in cardiac MRI. JPF is a radiologist and Director of Magnetic Resonance Research at UCLA. AK, DL and JL are cardiologists. AY is a bioengineer and PI of the Cardiac Atlas Project and head of Department of Anatomy and Medical Imaging at the University of Auckland. AS is an expert in atlas-based medical image analysis.

## Supplementary Material

GIGA-D-16-00049_Original_Submission.pdf

GIGA-D-16-00049_Revision_1.pdf

GIGA-D-16-00049_Revision_2.pdf

Response_to_reviewer_comments_Original_Submission.pdf

Response_to_Reviewer_Comments_Revision_1.pdf

Reviewer_1_Report_(Original_Submission).pdf

Reviewer_1_Report_(Revision_1).pdf

Reviewer_2_Report_(Original_Submission).pdf

Reviewer_3_Report_(Original_Submission).pdf

Reviewer_3_Report_(Revision_1).pdf

## Footnotes

1
http://www.cardiacatlas.org/tools/lv-shape-orthogonal-clinical-modes/

## Appendix

### Principal component regression

Let $\boldsymbol{X} \in {\mathbb{R}^{N \times P}}$be a data matrix of predictor variables where each row is a case (shape vector) and each column a shape feature (in our case [x y z] coordinates of sampled points). There are N cases and P shape features. We first “column center” the data by subtracting the mean across cases.

PCA decomposes ***X*** into an othonormal matrix $\boldsymbol{\Phi } \in {\mathbb{R}^{P \times M}}\ $containing eigenvectors of the covariance matrix ***X***^T^***X***. The columns of ***Φ*** define “shape components”. M is the number of shape components used to approximate ***X***, typically M < P, by

(A.1) \begin{equation*}{\boldsymbol{X}_{est}}{\rm{\ }} = {\rm{\ }}\boldsymbol{T}{\boldsymbol{\Phi }^T} \end{equation*}

(A.2) \begin{equation*}\boldsymbol{T}{\rm{\ }} = {\rm{\ }}\boldsymbol{X\Phi }, \end{equation*}

where $\boldsymbol{T} \in {\mathbb{R}^{N \times M}}{\rm{\ }}$is a matrix of “scores.” Each case is thus approximated by a linear combination of shape components. The weights of the combination (rows of ***T***) are the amount of each shape component present in that case, and are calculated by projecting each shape vector onto the shape component.

In principal component regression (PCR), the response or dependent variable ***Y*** (at present we consider a single response variable being a centered remodeling index such as EDVI) is regressed against the principal component scores (scores being used as predictor variables):

(A.3) \begin{equation*}{\boldsymbol{Y}_{est}}\ = \ \boldsymbol{T}{\boldsymbol{B}_{PCR}},\ \end{equation*}

where ***B***_*PCR*_ is a vector of regression coefficients.

The advantage of this method is that the regression coefficients do not suffer from the well-known multicolinearity problem, in which the regression coefficients can be ill-defined if the independent variables are correlated, leading to instability in future predictions. Note that in PCA the resulting scores ***T*** are orthogonal, so the resulting scores have zero correlation within the dataset between different component scores.

### PCR remodeling component

The PCR can be written as

(A.4) \begin{equation*} {\boldsymbol {Y}_{est}} = {\boldsymbol T}{\boldsymbol {B}_{PCR}} = \boldsymbol {X\Phi }{\boldsymbol {B}_{PCR}} = {\boldsymbol{X\beta }}_{PCR}^{\prime} \end{equation*}

Here ***X*** are the predictor variables and the regression coefficients are calculated from the PCR as ${\boldsymbol \beta }_{PCR}^{\prime} = {\boldsymbol \Phi }{\boldsymbol {B}_{PCR}}$. This vector of regression coefficients can be thought of as the linear combination of shape components that best predict the response variable. We define a “PCR remodeling component” ${\boldsymbol \beta }_{PCR}^{}$by normalizing ${\boldsymbol \beta }_{PCR}^{\prime}$ (note the data and response are centered so we exclude the zero intercept). The PCR remodeling scores are defined as follows:

(A.5) \begin{equation*} {\boldsymbol {Y}_{PCRscore}} = \frac{{\boldsymbol {X\beta }_{PCR}^\prime}}{{\left| {\boldsymbol {\beta }_{PCR}^{\prime}} \right|}} = {\boldsymbol X}{\boldsymbol {\beta }_{PCR}} \end{equation*}

The remodeling score for each case is then a projection (inner product) of the shape vector on the remodeling component. The remodeling component is defined by analogy to PCA shape components as a unit length direction in shape space. Remodeling scores are defined by analogy to shape scores in PCA; we can get the estimated remodeling index from ***Y***_*PCRscore*_ by scaling by the norm of ${\boldsymbol \beta }_{PCR}^{\prime}$ and adding the mean.

### Partial least squares regression

A problem with PCR is that the independent variables are chosen by their ability to explain variance in ***X***, not ***Y***. PLS regression solves this problem by finding the “latent factors” that best explain the covariance between ***Y*** and ***X***. These are ranked from largest to smallest covariance, so the first factor explains the most covariance, the second factor for the second largest covariance, and so on.

PLS finds a linear decomposition of ***X*** and ***Y*** such that

(A.6) \begin{equation*} {\boldsymbol X} = {\boldsymbol T}{\boldsymbol {\Psi }^T} + {\boldsymbol {E}_X} \end{equation*}

(A.7) \begin{equation*}\boldsymbol{Y}{\rm{\ }} = {\rm{\ }}\boldsymbol{U}{\boldsymbol{\Omega }^T} + {\boldsymbol{E}_Y}, \end{equation*}

where ${\boldsymbol T} \in {\mathbb{R}^{N \times M}}$ and ${\boldsymbol U} \in {\mathbb{R}^{N \times M}}$ are PLS scores for predictor and response variables, respectively. Similarly, $\boldsymbol{\Psi } \in {\mathbb{R}^{P \times M}}$ and $\boldsymbol{\Omega } \in {\mathbb{R}^{K \times M}}$ (K = 1 for a single response variable) are the PLS loadings for the predictor and response variables. Unlike PCR, Ψ and ***Ω*** are not orthogonal and not normalized. The parameter *M* ≤ *P* is the number of latent factors, typically determined by examining the percentage variance explained in ***Y***.

PLS derives the ***β*** regression coefficients as linear combinations of the latent factors, which are chosen to maximize correlation between response and predictor variables. Several variants exist in the literature, differing in the calculation of ***T*** [21, 22]. However, similar to PCR, we can define PLS remodeling components and remodeling scores as

(A.8) \begin{equation*}{\boldsymbol {Y}_{PLSscore}}\ = \ \frac{{\boldsymbol{X\beta }_{PLS}^{\prime}}}{{\left| {\boldsymbol{\beta }_{PLS}^{\prime}} \right|}}\boldsymbol{\ } = \boldsymbol{\ X}{\boldsymbol{\beta }_{PLS}} \end{equation*}

As for PCR, the estimated ***Y*** can be derived from the scores by scaling by $| {\boldsymbol{\beta }_{PLS}^{\prime}} |$ and adding the mean.

### Orthogonal remodeling components

The orthogonalization process given in (3) can be applied to the results of PCR or PLS regression. PLS regression is always more efficient than PCA regression, in that fewer terms are required to capture the variance of the response variable. However, if all PCA components are included in the PCR, and all latent factors in the PLS, the two methods are equivalent. One-factor PLS (i.e., M = 1 in the PLS regression) has particular properties that may make it attractive in some applications. For example, one-factor PLS has been shown to be equivalent to rescaled ridge regression as the ridge parameter tends to infinity [22].

For K > 1, that is, more than one response variable included in Y, the PLS regression finds latent factors that explain the most covariance between the ***X*** and ***Y*** matrices simultaneously. This was not considered for the current work, because the resulting regression coefficients are not orthogonal.
