# Supplementary material for: Orthogonal decomposition of left ventricular remodeling in myocardial infarction
Source: Gigascience. 2017 Feb 6;6(3):1–15. doi: 10.1093/gigascience/gix005 (PMC5791439; doi:10.1093/gigascience/gix005)
Supplement: GIGA-D-16-00049_Original_Submission.pdf [file gix005_giga-d-16-00049_original_submission.pdf]

Manuscript

Click here to view linked References

Click here to download Manuscript Orthogonal PLS 2016-07-27.docx

Orthogonal Decomposition of Left Ventricular Remodelling in Myocardial Infarction

Xingyu Zhang[1], MHS, Pau Medrano-Gracia[1], PhD, Bharath Ambale-Venkatesh[2], PhD, David A Bluemke[3], MD, Brett R Cowan[1], MBChB, J Paul Finn[4], MD, Alan H Kadish[5], MD, Daniel C Lee[5], MD, Joao AC Lima[2], MD, Alistair A Young[1], PhD, Avan Suinesiaputra[1], PhD.

[1] Department of Anatomy and Medical Imaging, University of Auckland, Auckland, New Zealand,

[2] The Donald W. Reynolds Cardiovascular Clinical Research Center, The Johns Hopkins University, Baltimore, USA.

[3] National Institute of Biomedical Imaging and Bioengineering, Bethesda, Maryland, USA,

[4] Department of Radiology, UCLA, Los Angeles, USA.

[5] Feinberg Cardiovascular Research Institute, Northwestern University Feinberg School of Medicine, Chicago, USA

Abstract

Background: Left ventricular size and shape is important for quantifying cardiac remodelling in response to cardiovascular disease. Geometric *remodelling indices* have been shown to have prognostic value in predicting adverse events in the clinical literature, but these do not independently describe shape changes. We developed a novel method for deriving orthogonal shape components directly from any set of clinical indices. Six clinical remodelling indices (end-diastolic volume index, sphericity, relative wall thickness, ejection fraction, apical conicity and longitudinal shortening) were evaluated using cardiac magnetic resonance images of 300 patients with myocardial infarction, and 1,991 asymptomatic subjects, obtained from the Cardiac Atlas Project.

Results: Partial least squares (PLS) regression of left ventricular shape models resulted in shape components that were optimally associated with each remodelling index. A Gram–Schmidt orthogonalization process, by which components were removed from the shape space in order of variance explained, resulted in a set of orthogonal shape components. A single PLS hidden variable per clinical index resulted in the greatest decorrelation between scores, and complete decorrelation with all previously removed remodelling indices.

Conclusions: The PLS orthogonal remodelling components had similar power to describe differences between patients and subjects as principal component analysis, but were more correlated to well-understood clinical indices of cardiac remodelling. The data and analyses are available from www.cardiacatlas.org.

Keywords: cardiac remodelling, magnetic resonance imaging, feature extraction, partial least squares regression.

Background

Left ventricular (LV) remodelling refers to the process by which the heart adapts its size, shape and function in response to disease processes, or under the influence of mechanical, neurohormonal and genetic factors [1]. Remodelling can be compensatory, for example increased concentric hypertrophy in hypertension, or adverse, for example increased end-systolic volume after myocardial infarction. Adverse LV remodelling characteristics after myocardial infarction provide important diagnostic and prognostic information for the therapeutic management of disease progression [2-5]. Clinical studies have identified quantitative geometric parameters (termed *remodelling indices* in this paper) that describe recognised clinical patterns of remodelling with prognostic value for predicting adverse events. For example, increased LV volume index has been shown to be an important predictor of mortality after myocardial infarction [6]. Increased LV sphericity has also been linked with decreased survival [5]. Relative LV wall thickness [1] and apical conicity [7] are also important indices of adverse remodelling after myocardial infarction. Functional parameters such as ejection fraction (EF), which is the most common index of cardiac function performance in clinical practice, are also heavily influenced by the degree of LV remodelling [8, 9]. LV longitudinal shortening is also a sensitive marker of LV remodelling [10].

Although these clinical remodelling indices have validated prognostic value, they are often co-dependent and do not provide an orthogonal decomposition of cardiac shape. Such an orthogonal decomposition would enable computational analysis of the independent components of remodelling present in various forms of heart disease. In particular, orthogonal shape decompositions enable simplified tensor calculus in the computation of e.g. arc lengths and areas, because they do not present off-diagonal terms in their metric tensor [11]. An orthogonal basis for shape enables robust calculation of contribution of each

component independently to the overall shape. Also, regressions using orthogonal shape components as independent variables do not suffer from the problem of multicolinearity. Thus, when analysing the combined effects of different remodelling characteristics, it is preferred to have an orthogonal basis in a linear space.

Principal component analysis (PCA) [12] is a powerful and widely used shape analysis technique that provides an orthogonal linear shape basis. In previous work, PCA analysis of cardiac remodelling has achieved more powerful descriptions of remodelling, and their relationships with risk factors, than traditional mass and volume analysis [13]. In a large population study, the first and second PCA components corresponded with LV size and sphericity respectively [14]. However, PCA shape components do not generally relate to clinical remodelling indices, making clinical interpretation of the relative contribution of shape components difficult. Remme *et al.* [15] developed a method to decompose shape changes into modes with clear clinical interpretation. However, these modes were not orthogonal.

In this paper, we used partial least squares (PLS) regression to sequentially construct an orthogonal shape decomposition that is optimally related to clinical remodelling indices. At each step, the contribution of the previous component was removed mathematically from the shape description, similar to Gram–Schmidt orthogonalization. Clinical remodelling indices of end-diastolic volume index (EDVI), sphericity, ejection fraction, relative wall thickness, conicity and longitudinal shortening, known from the literature to have important prognostic information in the management of myocardial infarction, were used to create corresponding orthogonal components from the shape parameters. By using a single PLS hidden variable per clinical index, the resulting component scores were maximally de-correlated, and completely de-correlated with those clinical indices previously removed.

Data Description

Patient Data

LV shape models of 300 patients with myocardial infarction and 1,991 asymptomatic study subjects were obtained through the Cardiac Atlas Project [16]. The patient data have been described previously [13] [17] and are available from the Cardiac Atlas Project (http://www.cardiacatlas.org). Briefly, myocardial infarction patients (n=300, age 31−86, mean age 63, 20% women) had clinical history of myocardial infarction with EF>35% and infarct mass >10% of LV myocardial mass. Asymptomatic subjects (n=1991, age 45−84, mean age 61, 52% women) did not have physician-diagnosed heart attack, angina, stroke, heart failure of atrial fibrillation, and had not undergone procedures related to cardiovascular disease, at the time of recruitment [13] [17].

Finite element shape models were customized to cardiac MRI exams in each case using a standardized procedure [13]. The shape models were evenly sampled at sufficient resolution to capture all visible features, which resulted in 1,682 Cartesian (x_i, y_i, z_i) points in homologous anatomical locations for each LV model.

Clinical Remodelling Indices

Clinical remodelling indices included EDVI, EF, relative wall thickness, sphericity, apical conicity and longitudinal shortening. LV mass and volumes were calculated by numerical integration of the LV shape models. EDVI was calculated as EDV divided by body surface area. Ejection fraction was calculated as (EDV-ESV)/EDV. Relative wall thickness was defined as twice the posterior wall thickness divided by the end-diastolic diameter [18] at mid-ventricle. Sphericity was calculated as the EDV divided by the volume of a sphere with a diameter corresponding to the major axis at end-diastole in LV long axis view [19]. Apical conicity was calculated as the ratio of the apical diameter (defined as the

endocardium one third above the apex) over the basal diameter [7] at end-diastole. Longitudinal shortening was calculated as the difference of the distance of the central basal point to the apical point at end-diastole and end-systole over the distance at end-diastole.

Partial Least Squares Regression

Partial least squares (PLS) regression [20, 21] is a statistical method that is related to principal components regression; however, instead of using independent variables derived from their ability to explain variance in the predictive variables only, PLS finds independent variables by projecting both the predicted variables and the predictor variables to a new space, typically with reduced dimension, chosen to maximize the correlation between predicted and predictor variables. PLS is typically used to find the fundamental relations between the predicted and predictor variables, i.e. a latent variable approach to modelling the covariance structures in these two spaces.

Mathematically, let X represent the data matrix where each row contains the coordinates of 3D points describing the shape of one case at ED, concatenated with the points at ES. In our application all asymptomatic and myocardial infarction cases were included in this matrix. Given a vector of clinical remodeling indices (e.g. EDVI) denoted as Y, PLS finds a linear decomposition of both X and Y such that

$$X = TP^T + E$$
$$Y = UQ^T + F$$

where T and U are, respectively, the projections of X and Y (also termed *scores*); P and Q are the loading matrices of reduced dimensionality N_{latent} (i.e. the number of latent variables used in the PLS decomposition) and E and F are the error terms assumed to be independent and identically distributed random normal variables. This decomposition is optimised to maximise the covariance between T and U [20].

The regression coefficients are also calculated so that $Y = \begin{bmatrix} 1 & X \end{bmatrix} \mathrm{B} + Yresiduals$, where B is a matrix of coefficients including the intercept. In this paper we used the SIMPLS algorithm as provided by the Statistics and Machine Learning Toolbox (MATLAB R2013a, The MathWorks, Inc., Natick, Massachusetts, United States). In this implementation T is orthonormal, but P, Q and U are not. However, each column of U is orthogonalised with respect to preceding columns of T, so that $T^T U$ is lower triangular.

Orthogonalization of PLS Components

The orthogonal remodelling components were created using PLS sequentially as shown in Figure 1. EDVI was selected as the first component, because it accounts for the greatest variance in the LV shape [13]. The contribution of this component was then removed from the shape description, by using a mathematical formulation similar to the Gram–Schmidt orthogonalization algorithm [22], prior to the calculation of the following components. This step ensures orthogonality in the new shape basis. The other remodelling components were calculated by PLS regression to the remaining clinical indices, using the deflated shape space. The component explaining the greatest variance in shape was then chosen as the next component to be removed from the shape space. This procedure was performed iteratively until all components were explained. The final order of the components was: (1) EDVI, (2) sphericity, (3) ejection fraction, (4) relative wall thickness, (5) conicity and (6) longitudinal shortening.

Following Figure 1, using super-indices to enumerate steps and sub-indices for columns, let $X = X^0$, the original shape space, and $Y = Y^1$, the first clinical index, i.e. EDVI. After the PLS regression, we define our "PLS component" as the normalized PLS regression coefficients B^1 omitting the intercept term. This is a vector in shape space that is maximally

correlated with EDVI. The projections associated with that component are $X^0\mathrm{B}^1$, and these were used as the component scores in the remodelling analysis below.

The shape space is then deflated by the EDVI-derived PLS component, giving rise to a new data matrix X^1: $X^1 = X^0 - X^0\mathrm{B}^1(\mathrm{B}^1)^\mathrm{T}$. The next clinical index is then chosen as the one that explains the most variation of the population in the new shape space, and the PLS regression and deflation are repeated for the remaining indices Y^k where at each step the previous PLS component contribution is removed:

$$X^\mathrm{k} = X^{\mathrm{k-1}} - X^{\mathrm{k-1}}\mathrm{B}^\mathrm{k}(\mathrm{B}^\mathrm{k})^\mathrm{T}$$

Note that subsequent B^{k+1} will be orthogonal to B^k by construction since X^{k+1} is orthogonal to B^k. Therefore the set of basis vectors B^k generate an orthogonal linear sub-space of X^0.

Number of latent variables

Selection of the number of latent variables N_{latent} is critical for obtaining PLS regression models with good predictive ability [23]. However, there is currently no standard method to choose the number of latent variables for PLS. We compared PLS regression results with N_{latent} =1 and N_{latent} =10. Results for N_{latent} >10 were similar to N_{latent} =10 because 10 latent variables accounted for most of the covariance between Y and X. Experiments for $1 < N_{latent} < 10$ gave intermediate results.

Characterization of myocardial infarction

To assess the clinical applicability of the orthogonal remodelling components, we analysed how these components were associated with myocardial infarction. Logistic regression models [24] were used to evaluate the discriminatory power of the orthogonal remodelling components to characterize LV remodelling due to myocardial infarction. Confounding

factors (age, gender, BMI, SBP, smoking status and diabetes history) were included in each regression model as baseline variables (covariates). Four logistic regression models were examined. Model 1 consisted of the baseline variables and the first 6 PCA scores. This was used as a reference for comparison. Model 2 consisted of the baseline variables and the clinical remodelling indices. Model 3 included the baseline variables and the orthogonal component scores for N_{latent} =1. Model 4 included the baseline variables and the orthogonal component scores for N_{latent} =10.

Four commonly-used measures were used to quantify the goodness-of-fit of the regression models: Deviance, Akaike information criterion (AIC), Bayesian information criterion (BIC) and the area under the receiver operating characteristic curve (AUC) [13]. Smaller Deviance, AIC and BIC, and larger AUC, are indicative of better goodness-of-fit.

Analyses

Participant characteristics are summarised in Table 1. Demographic characteristics were significantly different between the asymptomatic subjects and the myocardial infarction cases, including gender ratio, age, height, weight, blood pressure, diabetes history and smoking status. Clinical LV remodelling indices were also significantly different. The myocardial infarction patients had larger LV EDVI and ESV, increased sphericity, thicker walls, less conicity, smaller EF and reduced longitudinal shortening than the asymptomatic subjects.

The orthogonal PLS components corresponding to EDVI, sphericity, ejection fraction, relative wall thickness, conicity and longitudinal shortening, computed across all patient and asymptomatic cases, are shown in Figure 2 (N_{latent} =1) and Figure 3 (N_{latent} =10). Linear correlation coefficients were calculated between the clinical indices and the component scores in the combined population. Linear correlation coefficients between all PLS component scores and clinical indices are reported in Table 2 for N_{latent} =1 and in Table 3 for

N_{latent} =10. The linear correlation coefficients among the clinical indices are shown in Table 4, among the PLS component scores are shown in Table 5 for N_{latent} =1 and in Table 6 for N_{latent} =10. Correlation coefficients between clinical indices and scores of the first six PCA components of the original dataset are shown in Table 7 for comparison with PLS components in Tables 2 and 3.

The minimum correlation between remodelling scores was achieved with N_{latent} =1 (Table 5), because the PLS regression with only one latent variable finds the single shape vector that is maximally correlated with the clinical index. A single latent variable also resulted in complete decorrelation between the remodelling scores and the remodelling indices of all the components previously removed in the Gram-Schmidt procedure (Table 2).

Using more latent variables resulted in a subspace that yielded better correlation between each score and its corresponding index (diagonal elements are higher in Table 3 than in Table 2). However the deflated shape space retains correlation with the index.

Figure 4 shows the shape variance explained by each one of the PLS components for N_{latent}=1, N_{latent}=10 and by PCA. The total variance explained was the highest for PCA components (75.7% for 6 components). For PLS with N_{latent}=1, variance explained was 66.49% for 6 components, whereas for N_{latent} =10 it was 15.0%. This reflects the fact that PLS is designed to explain covariance between indices and shapes, rather than variance in the shapes themselves.

The results of logistic regression models to characterize remodelling associated with myocardial infarction using the orthogonal remodelling components are shown in Table 8. The scores from all orthogonal remodelling components showed significant odds ratios. The odds ratio of EDVI, sphericity, wall thickness, conicity, ejection fraction and longitudinal

shortening component scores indicate that myocardial infarction patients tend to have larger and more spherical LV shapes with thinner walls, and a less conical shape.

Table 9 shows the comparisons of the regression models with the baseline models. All three regression models showed significant improvement compared with the baseline model. The logistic regression based on orthogonal remodelling components showed smaller Deviance, AIC and BIC and higher AUC than the PCA logistic regression. The AUC (Figure 5) for the PLS components with a single latent variable was 97.38%, slightly greater than that for the PLS components with 10 latent variables (95.99%), or the logistic classification using scores from the first 6 PCA components (97.28%), or the logistic classification using the corresponding clinical indices (95.96%). The PLS components with a single latent variable (Model 3) obtained the best classification power and goodness-of-fit measures.

The standardized coefficients of the logistic regression model were used to create a linear combination of the PLS (N_{latent} =1) components generating a combined remodelling score, called the LR score (Figure 6), separating the two groups. The median LR scores (Model 3) for all cases were calculated and the median shapes were calculated by projecting the coefficients of the PLS components estimated in the logistic regression model back on the population shape space. These are plotted in Figure 6. This graphically shows the shape changes which best distinguish the two groups with baseline variables adjusted, showing that LV remodelling due to myocardial infarction is associated with larger volume, more spherical shape, and thinner wall thickness. Since the logistic regression coefficients refer to contributions from remodelling components, the amount of each remodelling component contributing to the LR score could be quantified. This gives an intuitive explanation of the LR score in terms of remodelling components associated with clinical remodelling indices.

Discussion

Patients with myocardial infarction exhibit significant shape changes with respect to the normal population, due to cardiac remodelling. An atlas-based analysis of cardiac remodelling has previously shown better characterization of remodelling due to myocardial infarction than traditional mass and volume analysis in large data sets [13]. The framework consisted of three steps: (1) fitting a finite element model to the LV MR images, (2) feature extraction of the aligned shape parameters, and (3) quantification of the association between the features and disease using logistic regression. Although PCA provides orthogonal shape features, which describe the maximum amount of variation for the fewest number of components, these components typically do not correspond with clinical indices of cardiac remodelling. To avoid this problem, and maintain the advantages of orthogonality, we developed a method to generate orthogonal shape components from any set of clinical indices using PLS.

In this paper, we generated a linear orthogonal shape basis from the full finite element shape parameters. Clinical indices, such as EDVI, sphericity, ejection fraction, relative wall thickness, conicity and longitudinal shortening, were derived from the finite element shape model. Similar to PCA, the shape components derived from PLS regression are orthogonal (zero dot product between different component shape vectors). In PCA, the resulting component scores are also decorrelated across the population cohort, but this is not the case with PLS. Table 6 shows that PLS component scores with N_{latent} =10 were significantly correlated, similar to the original clinical indices in Table 4. This is expected since N_{latent} =10 results in strong correlations between scores and indices (Table 3). PLS components both using N_{latent} =10 and N_{latent} =1 obtain effective shape representation for each clinical index, as evidenced by the correlation coefficients with the clinical indices (diagonal terms in Tables 2 and 3). However correlations between the scores of different indices for PLS with N_{latent} =1

become smaller than the original indices and scores of PLS with N_{latent} =10. For example, the correlation between EDVI and EF was originally -0.60 (Table 4), then became -0.68 from PLS with N_{latent} =10 (Table 6); however it was -0.15 from PLS with N_{latent} =1 (Table 5).

Not only did a single latent variable result in the greatest decorrelation between component scores (Table 5), but it also resulted in total decorrelation between component scores and previously removed indices (upper triangle of Table 2).

These orthogonal components derived from traditional remodelling indices may be used to partition shape into contributions from each component, independent of the others. Correlation analysis shows that these clinically derived components have high correspondence with traditional remodelling indices (diagonals in Tables 2 and 3), either virtually following the clinical indices' original correlation (Table 4) in N_{latent} =10 (Table 3), or by sacrificing some of the diagonal correlations in exchange for decoupling with previous indices in N_{latent} =1 (Table 2). Shapes features at N_{latent} =10 are more correlated with the original clinical indices than N_{latent} =1 but at the expense of their ability to explain variance in the original shape space (Figure 7). It can therefore be argued that N_{latent} =10 generates more 'specific' shapes with lesser representative power.

The results also show that clinically derived components quantitatively characterise remodelling features associated with myocardial infarction with similar power as PCA components. Three logistic regression models based on the clinical indices, PCA components and orthogonal remodelling components derived from clinical indices were all similar in terms of goodness of fit.

Coefficients of the PLS components estimated in the logistic regression model were projected back on the population shape space. By projecting these components back onto the population space (Figure 5), we can visualise the shape changes of the six features (the

change of EDVI, sphericity, EF, RWT, conicity and longitudinal shortening) due to remodelling. This combined component can be used for tracking individual patients over time in future studies, by quantifying the degree to which their LV shapes compare with the remodelling spectrum.

Supervised feature extraction techniques such as information maximising component analysis and linear discriminate analysis have also been used to extract a remodelling component which can best characterize myocardial infarction using surface sampling [25]. In the current study, the shape features of each clinical index were obtained first and then combined using logistic regression with baseline information removed. The shape changes due to myocardial infarction obtained by this LR model can be more easily explained as a combination of well-understood shape features, through the LR coefficients.

This method can be applied to any index with particular clinical utility, with visualization of their corresponding shape features and quantification of components, thereby further exploiting shape information in a clinically meaningful fashion.

Potential implications

This work enables precise multi-dimensional characterization of the ways in which the heart adapts with the progression of disease after myocardial infarction. The computed shape components are clinically meaningful since they are optimally related to indices with proven prognostic value. The resulting shape component scores can be used to track the progression of remodelling over time, against reference populations. This enables automatic computation of z-scores giving precise information on how the patient’s heart compares against the reference population.

Availability of supporting data and materials

LV shape models, clinical indices and orthogonal shape modes, together with code for their calculation and visualization, are available for public download at http://www.cardiacatlas.org/tools/lv-shape-orthogonal-clinical-modes/. Information on the original imaging studies can be found at http://www.cardiacatlas.org/studies/. DICOM image data and associated clinical variables are obtainable on request at http://www.cardiacatlas.org/data-access/request-cap-access/. Because of the variety of sources of imaging data, each with different IRB and steering committee requirements, the DICOM images and associated clinical information are not publically available; however, these data are made available to researchers on approval of a research application submitted under the Cardiac Atlas Project data sharing policy (www.cardiacatlas.org).

Declarations

Abbreviations and Acronyms

Left ventricular =LV， Ejection Fraction = EF, Principal Component Analysis = PCA, Partial Least Squares = PLS, End-diastolic Volume Index (EDVI), End-systolic Volume = ESV, Myocardial infarction =MI， Ejection fraction =EF， Longitudinal shortening =LS， End-systolic volume = ESV, end-diastolic volume = EDV, LV mass =LVM, left ventricle mass index= LVMI, Relative wall thickness = RWT, mass to volume ratio =MVR, Systolic Blood Pressure=SBP, Diastolic blood pressure=DBP

Ethics approval and consent to participate

This study was approved by the local institutional review boards (Johns Hopkins University School of Medicine NA_00031350; Northwestern University CR1_STU00000078; New

Zealand Multi-region Ethics Committee MEC/08/04/052) and all participants gave written informed consent.

Consent for Publication

Not applicable

Competing interests

None

Funding

This project was supported by award numbers R01HL087773 and R01HL121754 from the National Heart, Lung, and Blood Institute. MESA was supported by contracts N01-HC-95159 through N01-HC-95169 from the NHLBI and by grants UL1-RR-024156 and UL1-RR-025005 from NCRR. DETERMINE was supported by St. Jude Medical, Inc; and the National Heart, Lung and Blood Institute (R01HL91069). David A. Bluemke is supported by the NIH intramural research program. Xingyu Zhang was supported by the China Scholarship Council.

Authors' contributions

All authors were involved in the design of the study, interpretation of the data, drafting and revision of the manuscript, and final approval of the submitted manuscript. XZ, PM-G, and AS performed the statistical analyses.

Authors’ Information

XZ is a PhD student and biostatistician. PM-G is a biostatistician and expert in bioinformatics. BA-V is a bioengineer and expert in medical image analysis. DB is a radiologist and Director of Radiology and Imaging Sciences at the National Institute of Biomedical Imaging and Bioengineering and a co-PI of the MESA study. BR is a clinical engineer and an expert in

cardiac MRI. JPF is a radiologist and Director of Magnetic Resonance Research at UCLA and a co-PI of the DETERMINE study. AK is a cardiologist and PI of the DETERMINE study; DL is a cardiologist and Director of the DETERMINE MRI Core Lab. JL is a cardiologist and Director of the MESA MRI Core Lab. AY is a bioengineer and PI of the Cardiac Atlas Project and head of Department of Anatomy and Medical Imaging at the University of Auckland. AS is an expert in atlas-based medical image analysis.

References

1. Sutton MGSJ, Sharpe N: **Left Ventricular Remodeling After Myocardial Infarction: Pathophysiology and Therapy.** *Circulation* 2000, **101:**2981-2988.

2. Gjesdal O, Bluemke DA, Lima JA: **Cardiac remodeling at the population level[mdash]risk factors, screening, and outcomes.** *Nat Rev Cardiol* 2011, **8:**673-685.

3. Lieb W, Gona P, Larson MG, Aragam J, Zile MR, Cheng S, Benjamin EJ, Vasan RS: **The Natural History of Left Ventricular Geometry in the CommunityClinical Correlates and Prognostic Significance of Change in LV Geometric Pattern.** *JACC: Cardiovascular Imaging* 2014, **7:**870-878.

4. Zile MR, Gaasch WH, Patel K, Aban IB, Ahmed A: **Adverse left ventricular remodeling in community-dwelling older adults predicts incident heart failure and mortality.** *JACC: Heart Failure* 2014.

5. Wong SP, French JK, Lydon A-M, Manda SOM, Gao W, Ashton NG, White HD: **Relation of left ventricular sphericity to 10-year survival after acute myocardial infarction.** *The American journal of cardiology* 2004, **94:**1270-1275.

6. White HD, Norris RM, Brown MA, Brandt PW, Whitlock RM, Wild CJ: **Left ventricular end-systolic volume as the major determinant of survival after recovery from myocardial infarction.** *Circulation* 1987, **76:**44-51.

7. Di Donato M, Dabic P, Castelvecchio S, Santambrogio C, Brankovic J, Collarini L, Joussef T, Frigiola A, Buckberg G, Menicanti L, the RG: **Left ventricular geometry in normal and post-anterior myocardial infarction patients: sphericity index and 'new' conicity index comparisons.** *European Journal of Cardio-Thoracic Surgery* 2006, **29:**S225-S230.

8. Konstam MA, Kramer DG, Patel AR, Maron MS, Udelson JE: **Left Ventricular Remodeling in Heart FailureCurrent Concepts in Clinical Significance and Assessment.** *JACC: Cardiovascular Imaging* 2011, **4:**98-108.

9. Anand I, McMurray J, Cohn JN, Konstam MA, Notter T, Quitzau K, Ruschitzka F, Lüscher TF: **Long-term effects of darusentan on left-ventricular remodelling and clinical**

outcomes in the Endothelin A Receptor Antagonist Trial in Heart Failure (EARTH): randomised, double-blind, placebo-controlled trial. *The Lancet* 2004, **364:**347-354.

10. Kramer CM, Lima JA, Reichek N, Ferrari VA, Llaneras MR, Palmon LC, Yeh IT, Tallant B, Axel L: **Regional differences in function within noninfarcted myocardium during left ventricular remodeling.** *Circulation* 1993, **88:**1279-1288.
11. Synge JL, Schild A: *Tensor calculus.* Courier Corporation; 1969.
12. Jolliffe I: *Principal component analysis.* Wiley Online Library; 2005.
13. Zhang X, Cowan BR, Bluemke DA, Finn JP, Fonseca CG, Kadish AH, Lee DC, Lima JAC, Suinesiaputra A, Young AA, Medrano-Gracia P: **Atlas-Based Quantification of Cardiac Remodeling Due to Myocardial Infarction.** *PLoS ONE* 2014, **9:**e110243.
14. Medrano-Gracia P, Cowan BR, Ambale-Venkatesh B, Bluemke DA, Eng J, Finn JP, Fonseca CG, Lima JAC, Suinesiaputra A, Young AA: **Left ventricular shape variation in asymptomatic populations: the multi-ethnic study of atherosclerosis.** *Journal of Cardiovascular Magnetic Resonance* 2014, **16:**56.
15. Remme EW, Young AA, Augenstein KF, Cowan B, Hunter PJ: **Extraction and quantification of left ventricular deformation modes.** *IEEE Trans Biomed Eng* 2004, **51:**1923-1931.
16. Fonseca CG, Backhaus M, Bluemke DA, Britten RD, Do Chung J, Cowan BR, Dinov ID, Finn JP, Hunter PJ, Kadish AH: **The Cardiac Atlas Project—an imaging database for computational modeling and statistical atlases of the heart.** *Bioinformatics* 2011, **27:**2288-2295.
17. Medrano-Gracia P, Cowan BR, Bluemke DA, Finn JP, Kadish AH, Lee DC, Lima JAC, Suinesiaputra A, Young AA: **Atlas-based analysis of cardiac shape and function: correction of regional shape bias due to imaging protocol for population studies.** *Journal of Cardiovascular Magnetic Resonance* 2013, **15:**80.
18. Li L, Shigematsu Y, Hamada M, Hiwada K: **Relative Wall Thickness Is an Independent Predictor of Left Ventricular Systolic and Diastolic Dysfunctions in Essential Hypertension.** *Hypertension Research* 2001, **24:**493-499.

19. Izumo M, Lancellotti P, Suzuki K, Kou S, Shimozato T, Hayashi A, Akashi YJ, Osada N, Omiya K, Nobuoka S, et al: *Three-dimensional echocardiographic assessments of exercise-induced changes in left ventricular shape and dyssynchrony in patients with dynamic functional mitral regurgitation.* 2009.

20. Geladi P, Kowalski BR: **Partial least-squares regression: a tutorial.** *Analytica chimica acta* 1986, **185:**1-17.

21. de Jong S, Phatak A: **Partial least squares regression.** *Recent advances in total least squares techniques and errors-in-variables modeling* 1997**:**311-338.

22. Hoffmann W: **Iterative algorithms for Gram-Schmidt orthogonalization.** *Computing* 1989, **41:**335-348.

23. Gómez-Carracedo MP, Andrade JM, Rutledge DN, Faber NM: **Selecting the optimum number of partial least squares components for the calibration of attenuated total reflectance-mid-infrared spectra of undesigned kerosene samples.** *Analytica Chimica Acta* 2007, **585:**253-265.

24. Zhang X, Ambale-Venkatesh B, Bluemke D, Cowan B, Finn JP, Hundley W, Kadish A, Lee D, Lima JC, Suinesiaputra A, et al: **Orthogonal Shape Modes Describing Clinical Indices of Remodeling.** In *Functional Imaging and Modeling of the Heart. Volume* 9126. Edited by van Assen H, Bovendeerd P, Delhaas T: Springer International Publishing; 2015: 273-281: *Lecture Notes in Computer Science*].

25. Zhang X, Ambale-Venkatesh B, Bluemke D, Cowan B, Finn J, Kadish A, Lee D, Lima J, Hundley W, Suinesiaputra A, et al: **Information maximizing component analysis of left ventricular remodeling due to myocardial infarction.** *Journal of Translational Medicine* 2015, **13:**343.

Tables

Table 1 Baseline variables and clinical remodelling indices for asymptomatic subjects and patients with myocardial infarction (mean ± SD)

Variable	Unit	Asymptomatic	MI cases	p-value
Sex	F/M	1034/975	60/238	<0.01
Age	years	61.47±10.15	62.76±10.76	0.043
Height	cm	165.98±9.99	173.82±9.77	<0.001
Weight	kg	76.75±16.50	90.06+14.14	<0.001
BMI		27.77±5.09	29.73+5.57	<0.001
SBP	mmHg	126.28±21.98	126.36±17.50	>0.05
DBP	mmHg	71.49±10.33	73.26±9.82	0.006
Diabetes history	%	13.11	35.67	<0.001
Smoking status	%	12.51	11.33	>0.05
EDVI	ml/m^2	67.83±13.29	96.53±25.03	<0.001
Sphericity		0.38±0.08	0.41±0.09	<0.001
RWT	%	39.71±9.49	35.21±8.38	<0.001
Conicity		0.74±0.08	0.70±0.08	<0.001
EF		0.63±0.07	0.41±0.11	<0.001
LS		0.13±0.04	0.08±0.03	<0.001

MI=Myocardial infarction; BMI=Body mass index; SBP=Systolic blood pressure; DBP=diastolic blood pressure; EDV= end diastolic volume; RWT=relative wall thickness; EF= ejection fraction; LS=longitudinal shortening.

Table 2 Correlation coefficients between the clinical indices and the PLS component scores (N_{latent} =1)

	EDVI score	Sphericity score	EF score	RWT score	Conicity score	LS score
EDVI	0.82	0	0	0	0	0
Sphericity	0.03	0.83	0	0	0	0
EF	-0.75	0.03	0.61	0	0	0
RWT	-0.20	-0.16	-0.04	0.53	0	0
Conicity	-0.14	-0.28	0.30	0.21	0.72	0
LS	-0.45	0.03	0.61	-0.17	0.20	0.53

Table 3 Correlation coefficients between the clinical indices and the PLS component scores (N_{latent} =10)

	EDVI score	Sphericity score	WT score	EF score	Conicity score	LS score
EDVI	0.94	0.27	-0.64	-0.34	-0.13	-0.31
Sphericity	0.30	0.97	-0.16	-0.15	-0.25	-0.13
WT	-0.65	-0.12	0.99	0.26	0.25	0.53
EF	-0.41	-0.28	0.22	0.90	0.25	-0.02
Conicity	-0.13	-0.22	0.25	0.38	0.97	0.24
LS	-0.32	-0.13	0.56	0.02	0.25	0.98

Table 4 Correlation coefficients among the clinical indices.

	EDVI	Sphericity	EF	RWT	Conicity	LS
EDVI	1	0.28	-0.60	-0.37	-0.11	-0.29
Sphericity	0.28	1	-0.11	-0.28	-0.22	-0.13
EF	-0.60	-0.11	1	0.18	0.26	0.57
RWT	-0.37	-0.28	0.18	1	0.32	0.00
Conicity	-0.11	-0.22	0.26	0.32	1	0.26
LS	-0.29	-0.13	0.57	0.00	0.26	1

Table 5 Correlation coefficients among the PLS clinical modes' scores (N_{latent} =1)

	EDVI score	Sphericity score	EF score	RWT score	Conicity score	LS score
EDVI score	1	-0.29	-0.15	0.22	-0.15	-0.08
Sphericity score	-0.29	1	0.001	-0.04	0.01	0.22
EF score	-0.15	0.001	1	0.09	0.09	0.09
RWT score	0.22	-0.04	0.09	1	-0.08	0.002
Conicity score	-0.15	0.01	0.09	-0.08	1	0.16
LS score	-0.08	0.22	0.47	0.002	0.16	1

Table 6 Correlation coefficients among the PLS clinical mode scores (N_{latent} =10)

	EDVI score	Sphericity score	EF score	WT score	Conicity score	LS score
EDVI score	1	0.29	-0.68	-0.37	-0.15	-0.34
Sphericity score	0.29	1	-0.17	-0.15	-0.25	-0.14
EF score	-0.68	-0.17	1	0.27	0.25	0.53
WT score	-0.37	-0.15	0.27	1	0.31	-0.01
Conicity score	-0.15	-0.25	0.25	0.31	1	0.24
LS score	-0.34	-0.14	0.53	-0.01	0.24	1

Table 7 Correlation coefficients between the clinical indices and the first 6 modes of variation of X^0 using PCA

	PC 1	PC 2	PC 3	PC 4	PC 5	PC 6
EDVI	0.80	-0.01	-0.74	-0.18	-0.13	-0.45
Sphericity	-0.26	-0.80	0.19	0.19	0.30	0.06
EF	-0.01	0.09	-0.11	0.03	-0.09	-0.20
RWT	0.10	0.24	-0.21	-0.25	-0.25	-0.18
Conicity	0.10	0.13	-0.15	-0.11	-0.15	-0.14
LS	0.21	0.02	0.03	-0.15	0.50	0.37

Table 8 Four logistic regressions for myocardial infarction

Variable	Coefficient	Standard error	p value	Standardized Coefficient	Odds Ratio(OR)	OR 95% Confidence Interval	
Model 1: PCA modes + Baseline model							
PC 1*	2.647	0.177	<.0001	1.459	14.108	9.969	19.967
PC 2*	-0.605	0.102	<.0001	-0.334	0.546	0.447	0.666
PC 3	0.077	0.112	0.492	0.042	1.080	0.867	1.345
PC 4*	2.024	0.153	<.0001	1.116	7.571	5.610	10.217
PC 5*	0.394	0.106	0.0002	0.217	1.483	1.204	1.826
PC 6	-0.115	0.119	0.331	-0.064	0.891	0.706	1.124
Model 2: Clinical indices + Baseline model							
EDVI*	0.042	0.008	<.0001	0.420	1.043	1.028	1.059
Sphericity	0.003	0.014	0.803	0.015	1.003	0.977	1.031
EF	0.002	0.014	0.885	0.011	1.002	0.975	1.030
RWT *	-0.161	0.015	<.0001	-0.948	0.852	0.827	0.877
Conicity*	-0.037	0.016	0.020	-0.159	0.964	0.935	0.994
LS*	-0.148	0.037	<.0001	-0.327	0.862	0.802	0.927
Model 3: PLS modes (N_{latent} =1) + Baseline model							
EDVI score*	2.838	0.189	<.0001	1.565	17.078	11.782	24.756
Sphericity score*	0.895	0.125	<.0001	0.494	2.448	1.917	3.126
EF score*	-1.315	0.148	<.0001	-0.725	0.269	0.201	0.359
RWT score	-1.542	0.149	<.0001	-0.850	0.214	0.160	0.286
Conicity score	0.343	0.124	0.006	0.189	1.409	1.105	1.797
LS score*	-0.036	0.140	0.797	-0.020	0.965	0.733	1.269
Model 4: PLS modes (N_{latent} =10) + Baseline model							
EDVI score*	0.839	0.161	<.0001	0.463	2.315	1.688	3.175
Sphericity score*	-0.172	0.113	0.126	-0.095	0.842	0.675	1.050
EF score*	0.092	0.129	0.474	0.051	1.096	0.852	1.411
RWT score	-1.809	0.178	<.0001	-0.998	0.164	0.115	0.232
Conicity score*	-0.390	0.122	0.001	-0.215	0.677	0.533	0.859
LS score	-0.668	0.142	<.0001	-0.368	0.513	0.389	0.677

All the modes are adjusted for age, gender, BMI, SBP, smoking status and diabetes history. *p<0.05

Table 9 Comparison of the four logistic regression models

	Deviance	AIC	BIC	AUC
Baseline Model	1559	1573	1614	0.7441
Index model	704	730	804	0.9596
PCA model	606	632	707	0.9728
PLS model (N_{latent} =1)	**569**	**595**	**669**	**0.9738**
PLS model (N_{latent} =10)	686	712	786	0.9599

AIC = Akaike information criterion ; BIC =Bayesian information criterion; AUC =Area under the ROC curve.

Figure 1 Click here to download Figure Figure 1 .docx

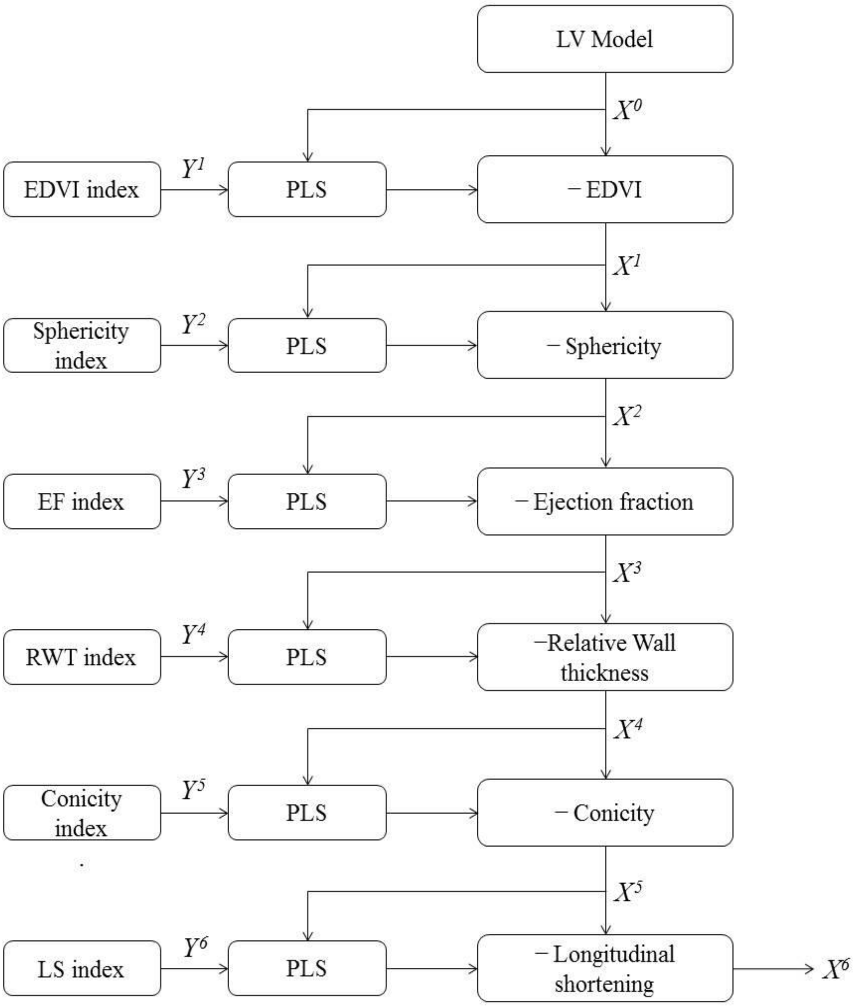

Figure 1 Data processing flow chart

Figure 2 Click here to download Figure Figure 2.docx

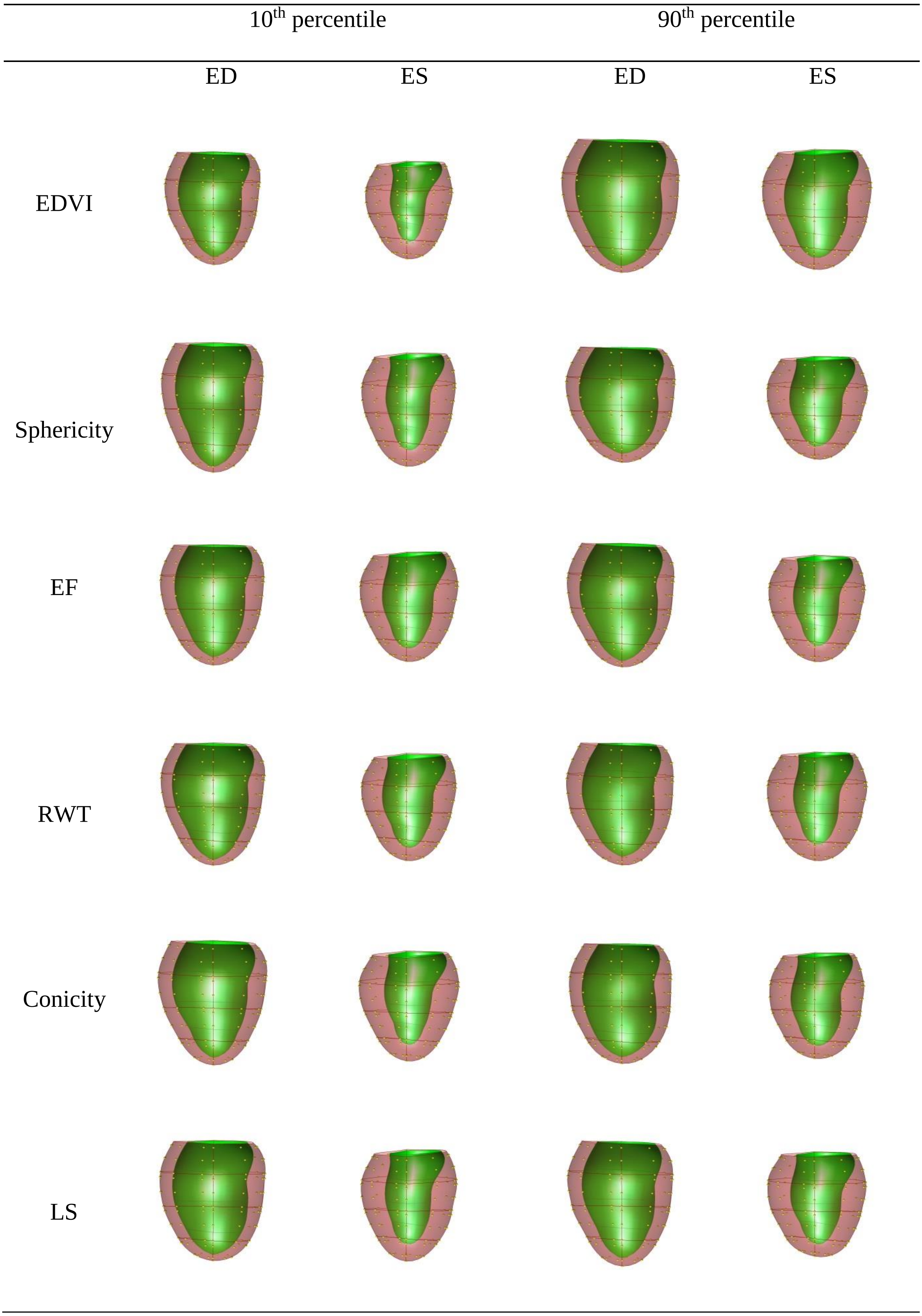

Figure 2 Plot of the PLS clinical components (N_{latent} =1)

Click here to download Figure Figure 3.docx

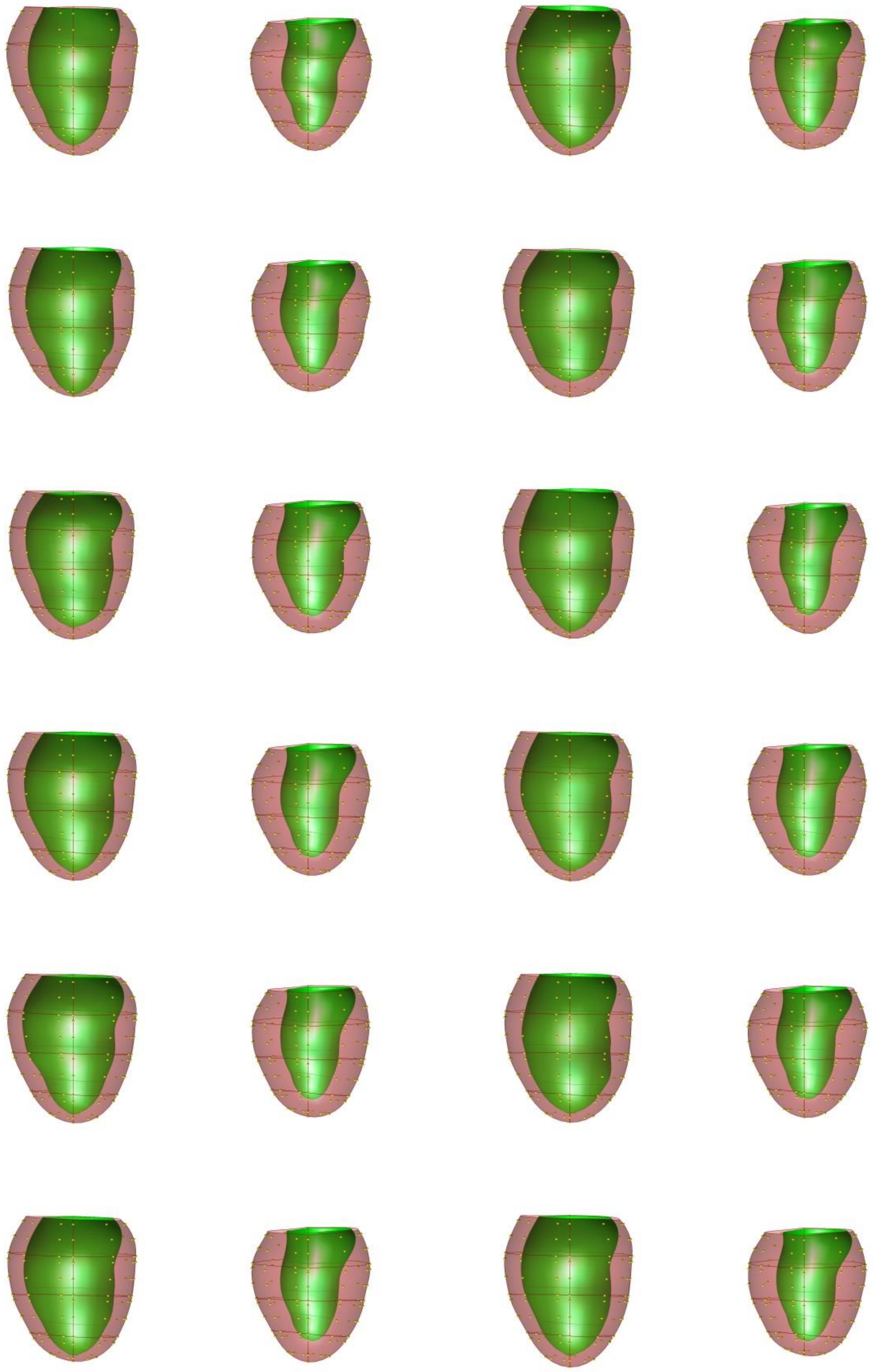

Figure 3 Plot of the PLS clinical components (N_{latent}=10)

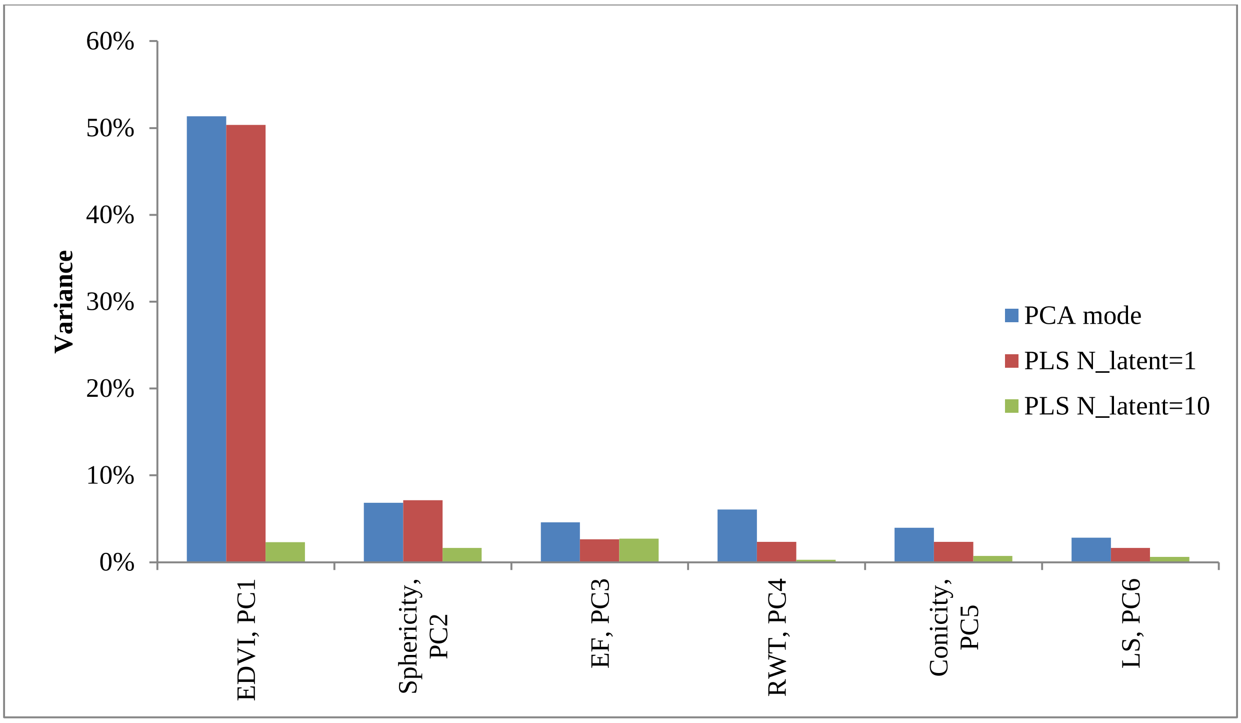

Figure 4 Variance explained by each PLS component and PCA component

 Click here to download Figure Figure 5 .docx

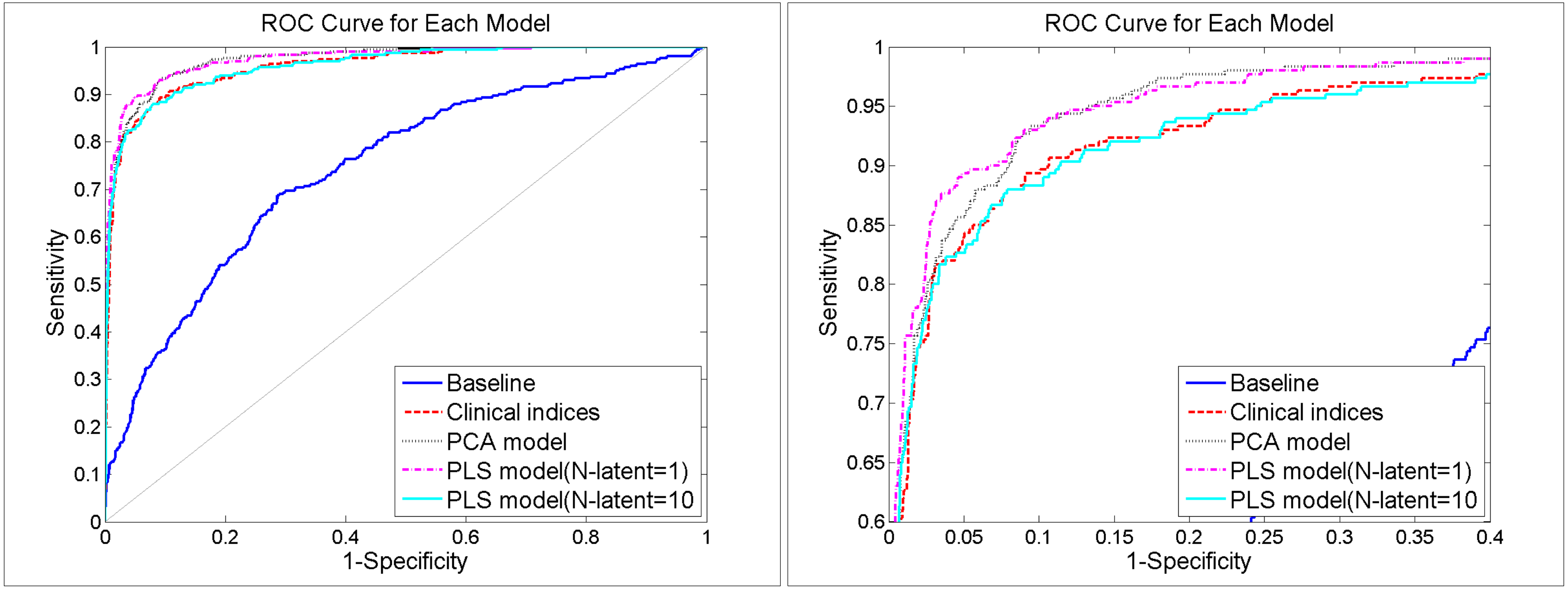

Figure 5 ROC curves for the five logistic regression models. The right figure shows a zoomed-in view to demonstrate the differences between the four models.

Figure 6 Click here to download Figure Figure 6.docx

Asymptomatic		MI Patients	
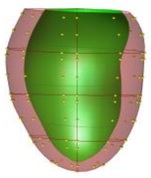	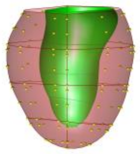	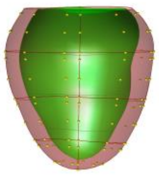	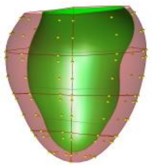
ED	ES	ED	ES

Figure 6 Visualization of shape changes between volunteers and patients, using the combined PLS (N_{latent} =1) component. Plots show the median LR score for the volunteer and patient groups respectively.
