# Supplementary material for: Orthogonal decomposition of left ventricular remodeling in myocardial infarction
Source: Gigascience. 2017 Feb 6;6(3):1–15. doi: 10.1093/gigascience/gix005 (PMC5791439; doi:10.1093/gigascience/gix005)
Supplement: GIGA-D-16-00049_Revision_1.pdf [file gix005_giga-d-16-00049_revision_1.pdf]

Manuscript

Click here to download Manuscript Orthogonal PLS - GigaScience - R1 markup.docx

Orthogonal Decomposition of Left Ventricular Remodelling in Myocardial Infarction

Xingyu Zhang[1], MHS, Pau Medrano-Gracia[1], PhD, Bharath Ambale-Venkatesh[2], PhD, David A Bluemke[3], MD, Brett R Cowan[1], MBChB, J Paul Finn[4], MD, Alan H Kadish[5], MD, Daniel C Lee[5], MD, Joao AC Lima[2], MD, Alistair A Young[1], PhD, Avan Suinesiaputra[1], PhD.

[1] Department of Anatomy and Medical Imaging, University of Auckland, Auckland, New Zealand,

[2] The Donald W. Reynolds Cardiovascular Clinical Research Center, The Johns Hopkins University, Baltimore, USA.

[3] National Institute of Biomedical Imaging and Bioengineering, Bethesda, Maryland, USA,

[4] Department of Radiology, UCLA, Los Angeles, USA.

[5] Feinberg Cardiovascular Research Institute, Northwestern University Feinberg School of Medicine, Chicago, USA

Abstract

Background: Left ventricular size and shape is important for quantifying cardiac remodelling in response to cardiovascular disease. Geometric *remodelling indices* have been shown to have prognostic value in predicting adverse events in the clinical literature, but these often describe interrelated shape changes. We developed a novel method for deriving orthogonal *remodelling components* directly from any (moderately independent) set of clinical remodelling indices. R2.2

Results: Six clinical remodelling indices (end-diastolic volume index, sphericity, relative wall thickness, ejection fraction, apical conicity and longitudinal shortening) were evaluated using cardiac magnetic resonance images of 300 patients with myocardial infarction, and 1,991 asymptomatic subjects, obtained from the Cardiac Atlas Project. Partial least squares (PLS) regression of left ventricular shape models resulted in *remodelling components* that were optimally associated with each remodelling index. A Gram–Schmidt orthogonalization process, by which remodelling components were successively removed from the shape space in the order of shape variance explained, resulted in a set of orthonormal remodelling components. *Remodelling scores* could then be calculated which quantify the amount of each remodelling component in each case. A one-factor PLS regression resulted in the least correlation between resulting remodelling scores, and zero correlation with all previously removed remodelling indices. R1.2

Conclusions: The PLS orthogonal remodelling components had similar power to describe differences between myocardial infarction patients and asymptomatic subjects as principal component analysis, but were better associated with well-understood clinical indices of cardiac remodelling. The data and analyses are available from www.cardiacatlas.org.

Keywords: cardiac remodelling, magnetic resonance imaging, feature extraction, partial least squares regression.

Background

Left ventricular (LV) remodelling refers to the process by which the heart adapts its size, shape and function in response to disease processes, or under the influence of mechanical, neurohormonal and genetic factors [1]. Remodelling can be compensatory, for example increased concentric hypertrophy in hypertension, or adverse, for example increased end-systolic volume after myocardial infarction. Adverse LV remodelling characteristics after myocardial infarction provide important diagnostic and prognostic information for the therapeutic management of disease progression [2-5]. Clinical studies have identified quantitative geometric parameters (termed *clinical remodelling indices* in this paper) that describe recognised clinical patterns of remodelling with prognostic value for predicting adverse events. For example, increased LV end-diastolic volume index (EDVI) has been shown to be an important predictor of mortality after myocardial infarction [6]. Increased LV sphericity has also been linked with decreased survival [5]. Relative LV wall thickness [1] and apical conicity [7] are also important indices of adverse remodelling after myocardial infarction. Functional parameters such as ejection fraction (EF), which is the most common index of cardiac performance in clinical practice, are also heavily influenced by the degree of LV remodelling [8, 9]. LV longitudinal shortening is another sensitive marker of LV functional remodelling [10].

Although these clinical remodelling indices have validated prognostic value, they are often R1.8
coupled so that it is difficult to separate the relative effects on heart shape. For example, end-diastolic volume is often correlated with ejection fraction in patients with myocardial infarction. It is therefore difficult to tease out the relative effects of dilatation (structural) from contraction (functional). In computational shape analysis, it is desirable to characterize the space of possible heart shapes in terms of orthogonal components. A shape component is R1.36
a unit vector in shape space, and orthogonal components have zero dot product between

different components. An orthogonal decomposition of heart shape, in which each component is related to a remodelling index with clear clinical importance, would assist clinical interpretation of the relative effects of different physiological processes underlying the development of disease. In addition, such an orthogonal decomposition would enable R2.4 computational analysis of a single component of remodelling present in various forms of heart disease. In particular, an orthogonal basis for shape enables robust calculation of contribution of each component independently to the overall shape. Also, regressions using orthogonal shape components as independent variables do not suffer from the problem of multicolinearity. Thus, when analysing the combined effects of different remodelling characteristics, it is preferred to have an orthogonal basis in a linear space.

Principal component analysis (PCA) [11] is a powerful and widely used shape analysis technique that provides an orthogonal linear shape basis. In previous work, PCA analysis of LV geometry has achieved more powerful descriptions of LV shape, and their relationships with risk factors, than traditional mass and volume analysis [12]. In a large population study, the first and second PCA LV shape components were associated with LV size and sphericity respectively [13]. However, PCA shape components are not designed to be related to any R1.9 particular clinical remodelling index, and the clinical interpretation of PCA shape components is often difficult. Previous work has shown that LV PCA shape components do not have clear clinical interpretation beyond the first two [12]. This is a common problem with PCA shape components, since they are designed to efficiently characterize shape variation without regard to possible underlying mechanisms of disease processes. Remme *et al.* [14] developed a method to decompose shape changes into modes with clear clinical interpretation. However, these modes were not orthogonal.

Decomposition of the shapes into orthogonal components enables calculation of scores as R1.2 R2.4 R3.5 projections of each patient’s shape onto the corresponding component (see Appendix). These

scores quantify the amount of each component present in the patient. One advantage of PCA shape components is that the resulting scores have zero correlation within the population (see Appendix). This is desirable in some applications, i.e. if the scores can be related to underlying processes, then low correlation between scores implies that the processes have different effects within the population.

Previously, orthogonal remodelling components were generated from clinical remodelling indices using an ad hoc approach [24]. For each clinical index, a subset of cases was chosen outside two standard deviations from the mean, i.e. those with very high and very low values of the clinical index. The remodelling component was then derived from these cases, by fitting a line between the two groups. The problem with this method is that it relied on extremes of the distribution of the clinical index and ignored the majority of cases. This may lead to difficulties in the interpretation of the remodelling component. Therefore, the current paper sought to provide the following novel contributions: i) calculation of remodelling components directly from regression coefficients, ii) use of the entire distribution of clinical index to formulate the remodelling component, and iii) reduction of correlation among resulting remodelling scores.

R1.4
R1.35

In this paper, we used partial least squares (PLS) regression to sequentially construct an orthogonal shape decomposition that is optimally related to clinical remodelling indices. Clinical remodelling indices of EDVI, sphericity, ejection fraction, relative wall thickness, conicity and longitudinal shortening, known from the literature to have important prognostic information in the management of myocardial infarction, were used to create corresponding orthogonal components from the shape parameters. By using a single PLS latent factor per clinical index, the resulting component scores were less correlated with each other, and had zero correlation with those clinical indices previously removed.

R1.5
R1.11

Data Description

Patient Data

LV shape models of 300 patients with myocardial infarction and 1,991 asymptomatic study subjects were obtained through the Cardiac Atlas Project [15]. The patient data have been described previously [12] [16] and are available from the Cardiac Atlas Project (http://www.cardiacatlas.org). Briefly, myocardial infarction patients (n=300, age 31−86, mean age 63, 20% women) had clinical history of myocardial infarction with EF>35% and infarct mass >10% of LV myocardial mass. All had stable myocardial infarction (i.e. no acute R2.12 cases). Asymptomatic subjects (n=1991, age 45−84, mean age 61, 52% women) did not have physician-diagnosed heart attack, angina, stroke, heart failure or atrial fibrillation, and had not undergone procedures related to cardiovascular disease, at the time of recruitment [12] [16].

Finite element shape models were customized to cardiac MRI exams in each case using a standardized procedure [12]. The shape models were evenly sampled at sufficient resolution to capture all visible features, which resulted in 1,682 Cartesian (x, y, z) points in homologous anatomical locations for each LV model.

Clinical Remodelling Indices

Clinical remodelling indices included EDVI, EF, relative wall thickness, sphericity, apical conicity and longitudinal shortening. Volumes were calculated by the summation of surface R1.15 triangle volumes [17]. LV mass was calculated by subtracting epicardial with endocardial volumes and then multiplied by 1.05 g/ml [18]. EDVI was calculated as endocardial surface R1.7 volume at end-diastole (EDV) divided by body surface area. Ejection fraction was calculated as (EDV-ESV)/EDV, where ESV is the endocardial surface volume at end-systole. Relative wall thickness was defined as twice the posterior wall thickness divided by the end-diastolic

diameter [19] at mid-ventricle. Sphericity was calculated as the EDV divided by the volume of a sphere with a diameter corresponding to the major axis at end-diastole in LV long axis view [20]. Apical conicity was calculated as the ratio of the apical diameter (defined as the diameter of the endocardium one third above the apex) over the basal diameter [7] at end-diastole. Longitudinal shortening was calculated as the difference of the distance between the centroid of the most basal ring of points to the most apical point at end-systole divided by the distance at end-diastole. R2.6 These indices not intended as a comprehensive list and were limited to geometric indices (i.e. ratios which correct for size in some sense), which have either been studied for many years (e.g. relative wall thickness as a measure of concentric versus eccentric hypertrophy), or can be readily calculated from several different imaging modalities (e.g. 3D echocardiography, MRI, or CT). Some attempts were made to only include indices that are moderately independent (e.g. end-systolic volume index was not included since it can be derived from end-diastolic volume index and ejection fraction). R2.5

Remodelling Components R1.1

In this paper, we use partial least squares (PLS) regression [21, 22] to explain each remodelling index $\boldsymbol{Y} \in \mathbb{R}^{N\times 1}$ with a linear combination of predictor variables $\boldsymbol{X} \in \mathbb{R}^{N\times P}$ so that

$$\boldsymbol{Y} = \boldsymbol{X}\boldsymbol{\beta}' + \boldsymbol{E}_Y \quad (1)$$

where $\boldsymbol{\beta}' \in \mathbb{R}^{P\times 1}$ is a vector of regression coefficients and $\boldsymbol{E}_Y$ is the residual vector. In this paper, the dimensions N and P denote the number of cases and the number of shape features (3D surface points) respectively.

Details of the PLS regression method in comparison with principal component regression are given in the Appendix. PLS regression calculates the regression coefficients $\boldsymbol{\beta}'$ as a linear

combination of M latent factors, where M<P. The latent factors are chosen to maximize the covariance between response and predictor variables.

In this paper, we use centered $\boldsymbol{Y}$ and $\boldsymbol{X}$ so that the intercept is zero. We define the normalized vector of regression coefficients (ignoring the intercept term) as the "remodelling component" associated with the corresponding remodelling index $\boldsymbol{Y}$. By analogy with PCA shape components, the remodelling component is a unit length vector in shape space (column space of X). We define "remodelling scores" by analogy with PCA scores, as the projection of each case onto the remodelling component:

$$\boldsymbol{Y}_{score} = \boldsymbol{X\beta} \qquad (2)$$

where $\boldsymbol{\beta}$ is the normalized regression coefficients. The estimated remodelling indices can be calculated from $\boldsymbol{Y}_{score}$ by scaling by the norm of $\boldsymbol{\beta}'$ and adding the mean index.

Orthogonal Remodelling Components

Orthogonal remodelling components are calculated following the flow chart in Figure 1. First, the remodelling index with the highest variance is chosen (EDVI). The corresponding remodelling component is calculated by PLS regression. Then a residual data matrix is generated by subtracting the projections of all cases onto the remodelling component: R1.16 R1.18 R1.22

$$\mathbf{X}^{(i+1)} = \mathbf{X}^{i} - \mathbf{X}^{i}\boldsymbol{\beta}^{i}\left(\boldsymbol{\beta}^{i}\right)^{T} \qquad (3)$$

for i=1,…,K, where K is the number of indices. The residual data matrix is then used in the next iteration to calculate the next remodelling component, associated with the remodelling index with the next highest variance in the data set (in this case the second index is sphericity). This process is repeated for all K=6 remodelling indices (Figure 1). The resulting orthonormal remodelling components $[\boldsymbol{\beta}^1, \boldsymbol{\beta}^2, \ldots, \boldsymbol{\beta}^K]$, form an orthogonal basis for a linear

sub-space of $\boldsymbol{X}$. Each $\boldsymbol{\beta}^{(i+1)}$ is orthogonal to the preceding $\boldsymbol{\beta}^{i}$ because the residual data matrix $\boldsymbol{X}^{(i+1)}$ is orthogonal to $\boldsymbol{\beta}^{i}$.

With this approach, the order of the response variables is important. We ordered the R2.7 remodelling indices based on their variance in remodelling scores over the population. This is a measure of the shape variance explained by each index. The order of remodelling indices was: 1) EDVI, 2) sphericity, 3) ejection fraction, 4) relative wall thickness, 5) conicity and 6) longitudinal shortening.

Number of latent factors

Selection of the number of latent factors *M* has a fundamental effect on the resulting R1.20 remodelling components. In the current context, there is no standard method to choose the R1.41 number of latent factors. In the context of prediction, cross-validation is commonly used to determine estimation error in the response variable [23]. We compared remodelling components and scores calculated from one-factor PLS (M=1) to multi-factor PLS up to M=30 (see Figure 2). Standard 10-fold cross-validation was performed to test estimation error, showing that 10 latent factors accounted for most of the mean squared error in estimating $\boldsymbol{Y}$. In term of remodelling components, results for M>10 were similar to M=10. Experiments for 1<M<10 gave intermediate results. Therefore, in the following, we only compared two remodelling models: one-factor PLS (M=1) and multi-factor PLS (M=10).

Characterization of myocardial infarction

We demonstrated the clinical applicability of our proposed shape decomposition method to analyse how these clinically driven remodelling components were associated with myocardial infarction, compared to the clinical indices themselves, or PCA shape components. Logistic regression models were used to evaluate the discriminatory power of the orthogonal

remodelling components to characterize LV remodelling due to myocardial infarction. Logistic regression is a common clinical tool for examining relative effects on disease, and relative strengths of associations with disease can be quantified using odds ratios. Confounding factors (age, sex, body mass index, diastolic blood pressure, smoking status and diabetes history) were included in each regression model as baseline variables (covariates), since there were significantly different between cohorts in Table 1. This was done to control for the effects of these confounding factors in each of the logistic regression models. Four logistic regression models were examined. Model 1 consisted of the baseline variables and the first 6 PCA component scores. This was used as a reference for comparison. Model 2 consisted of the baseline variables and the six clinical remodelling indices. Model 3 included the baseline variables and the orthogonal remodelling component scores for M=1. Model 4 included the baseline variables and the orthogonal remodelling component scores for M=10. In each case the presence or absence of symptomatic disease was defined by the dependant variable as 1 or 0 respectively.

R1.12
R2.18
R1.25
R2.14

Implementation

Codes were implemented in Matlab (Mathwork, Natick, MA) and R (The R Foundation, Vienna, Austria) programming languages, and are available from the Cardiac Atlas Project website[1]. The Matlab implementation requires the plsregress function from the Statistics and Machine Learning Toolbox. The R implementation requires the pls package [25]. We used SIMPLS algorithm [22] to compute the PLS regression in both versions due to its fast calculation. We compared the PLS regression coefficients using different methods provided by the pls package from R, i.e. kernel, wide kernel and classical orthogonal scores algorithms, and the results were very similar in the regression coefficients obtained.

R1.29
R2.19

[1] http://www.cardiacatlas.org/tools/lv-shape-orthogonal-clinical-modes/

Statistical analyses

Root mean square (RMS) errors in the angle between remodelling component unit vectors were used to quantify the differences arising from different training data sets: 1) asymptomatic cases from 100 to 1900, vs all asymptomatic cases, and 2) balanced data set (300 asymptomatic and 300 myocardial infarction) vs the full data set (1991 asymptomatic and 300 myocardial infarction).

For the logistic regression, the independent variables (components and baseline variables) R1.23 R1.28
were included simultaneously and the models were computed using SAS. A p value of <0.05 was considered significant. Four commonly-used measures were used to quantify the goodness-of-fit of the regression models: Deviance, Akaike information criterion (AIC), Bayesian information criterion (BIC) and the area under the receiver operating characteristic curve (AUC) [12]. Smaller Deviance, AIC and BIC, and larger AUC, are indicative of better goodness-of-fit. Statistical tests to determine whether the AUC of a model is significantly R1.55
greater or less than another model were performed using one-sided paired non-parametric tests for AUC values [26], implemented in the pROC package [27]. A p value of <0.05 was considered as statistically higher or smaller AUC value.

Results

Unless otherwise stated all experiments were performed including all cases (asymptomatic R1.40 R1.45
and MI patients). Participant characteristics are summarised in Table 1. Some demographic characteristics were significantly different between the asymptomatic subjects and the myocardial infarction cases, including gender ratio, age, height, weight, blood pressure, and diabetes history. Clinical LV remodelling indices were also significantly different, as expected. The myocardial infarction patients had larger LV EDVI, increased sphericity,

thicker walls, less conicity, smaller EF and reduced longitudinal shortening than the asymptomatic subjects.

The orthogonal PLS components corresponding to EDVI, sphericity, ejection fraction, relative wall thickness, conicity and longitudinal shortening, are visualized in Figure 3 (M=1) and Figure 4 (M=10). These visualizations are useful in understanding the effect of each R1.52 component on shape.

Linear correlation coefficients (Pearson) were calculated between the clinical indices and the R1.44 component scores in the combined population. Correlation coefficients between PLS remodelling scores and clinical indices are reported in Table 2 for M=1 and in Table 3 for M=10. A single latent factor resulted in zero correlation between the remodelling scores and the corresponding indices of all the components previously removed in the Gram-Schmidt procedure (Table 2). Using more latent factors resulted in better correlation between each remodelling score and its corresponding index (diagonal elements are higher in Table 3 than in Table 2). Correlation coefficients between clinical indices and scores of the first six PCA R1.49 components of the original dataset are shown in Table 4 for comparison.

The correlation coefficients among the clinical indices are shown in Table 5. These show R2.16 strong correlations between several clinical indices. The decreasing diagonal correlations in Tables 2 and 3 are likely due to this interdependence between clinical indices. Thus, RWT and LS are related to indices previously removed by the orthogonalization process (RWT is related to EDVI and sphericity, LS is related to EF, etc).

Correlations between the PLS remodelling scores are shown in Table 6 for M=1 and in Table 7 for M=10. The minimum correlation between remodelling scores was achieved with M=1 (Table 6).

A series of experiments was performed to compare remodelling components between the full data set (1991 asymptomatic + 300 myocardial infarction) with symmetric datasets, i.e. 300 asymptomatic and 300 MI patients) with 50 trials of randomly selected asymptomatic subsets. In this case, similar remodelling components are reflected by the same unit $\boldsymbol{\beta}$ vectors, which can be measured by angle differences between two $\boldsymbol{\beta}$ vectors. Figure 5a shows the root mean square errors (RMSE) of $\boldsymbol{\beta}$ vector differences between the subset and the full models. Only the first component (EDVI) showed less than 5 degrees differences, but increasing differences in other components were observed. This was expected since the characteristics of the cases included in the training set have an influence on the results. R2.20 R3.3

Considering only the asymptomatic cases, we investigated the differences in the remodelling components with different number of samples. Figure 5b shows the RMS errors of randomly sampled cases (50 trials each) with respect to the full 1991 cases. At least 1100 cases were needed to get below 10 degrees difference with the full cohort in all components.

The results of logistic regression models to characterize remodelling associated with myocardial infarction using the orthogonal remodelling scores are shown in Table 8. For the one-factor PLS remodelling scores, the odds ratio of EDVI, sphericity, ejection fraction, wall thickness, and conicity, indicate that myocardial infarction patients tend to have larger and more spherical LV shapes with thinner walls, and a less conical shape. The multi-factor PLS remodelling scores showed somewhat different results, with EDVI, EF, Conicity and longitudinal shortening scores being significant. This may be due to the increased multi-colinearity between remodelling scores in the multifactor case. R1.42

Table 9 shows the comparisons of the regression models. All four regression models showed significant improvement compared with the baseline variables alone. The logistic regression based on one-factor PLS orthogonal remodelling scores showed the best Deviance, AIC and R1.55 R2.14

BIC and AUC. The AUC (Figure 6) for the one-factor remodelling scores was significantly greater than the multi-factor remodelling scores, and the original clinical indices, but was not significantly different from the PCA model.

The standardized coefficients of the logistic regression model were used to create a linear combination of the PLS (M=1) components generating a combined remodelling score, called the logistic regression score, separating the two groups. The F logistic regression scores (Model 3) for all cases were calculated and the median shapes were calculated by projecting the coefficients of the PLS components estimated in the logistic regression model back on the population shape space. These are plotted in Figure 7. This graphically shows the shape changes which best distinguish the two groups with baseline variables adjusted, showing that LV remodelling due to myocardial infarction is associated with larger volume, more spherical shape, and thinner wall thickness. Since the logistic regression coefficients refer to contributions from remodelling components, the amount of each remodelling component contributing to the logistic regression score could be quantified. This gives an intuitive explanation of the logistic regression score in terms of remodelling components associated with clinical remodelling indices.

Discussion

Patients with myocardial infarction exhibit significant shape changes with respect to the normal population, due to cardiac remodelling. An atlas-based analysis of cardiac remodelling has previously shown better characterization of remodelling due to myocardial infarction than traditional mass and volume analysis in large data sets [12]. The framework consisted of three steps: (1) fitting a finite element model to the LV MR images, (2) feature extraction of the aligned shape parameters, and (3) quantification of the association between the features and disease using logistic regression. Although PCA provides orthogonal shape

features, which describe the maximum amount of variation for the fewest number of components, these components typically do not correspond with clinical indices of cardiac remodelling. To avoid this problem, and give the components a clear clinical interpretation, while maintaining the advantages of orthogonality, we developed a method to generate orthogonal shape components from any set of clinical indices using PLS. R1.3

In this paper, we generated a linear orthogonal shape basis from the full finite element shape parameters. Clinical indices, such as EDVI, sphericity, ejection fraction, relative wall thickness, conicity and longitudinal shortening, were derived from the finite element shape model. Similar to PCA, the shape components derived from PLS regression are orthogonal. In PCA, the resulting component scores also have zero correlation across the population cohort, but this is not the case with PLS. Table 7 shows that PLS component scores with M=10 were significantly correlated, similar to the original clinical indices in Table 5. This is expected since M=10 results in strong correlations between scores and indices (Table 3). PLS components both using M=10 and M=1 obtain effective shape representation for each clinical index, as evidenced by the correlation coefficients with the clinical indices (diagonal terms in Tables 2 and 3), compared to the first six components of PCA (Table 4).

We found that the correlations between the scores of different indices for PLS with M=1 become smaller than the original indices and scores of PLS with M=10. For example, the correlation between EDVI and EF was originally -0.60 (Table 5), then became -0.68 from PLS with M=10 (Table 7); however it was -0.15 from PLS with M=1 (Table 6). Not only did a single latent factor result in the least correlation between component scores (Table 6), but it also resulted in zero correlation between component scores and previously removed indices (upper triangle of Table 2).

These orthogonal components derived from traditional remodelling indices may be used to partition shape into contributions from each component, independent of the others. Correlation analysis shows that these clinically derived components have high correspondence with traditional remodelling indices (diagonals in Tables 2 and 3), either virtually following the clinical indices' original correlation (Table 5) in M=10 (Table 3), or by sacrificing some of the diagonal correlations in exchange for decoupling with previous indices in M=1 (Table 2). Remodelling scores at M=10 are more correlated with the original clinical indices than M=1 but at the expense of their ability to explain variance in the original shape space. It can therefore be argued that M=10 generates more 'specific' shapes with lesser representative power.

Previous studies have also used PLS to derive information on cardiac remodelling [28]. R3.1
Lekadir et al. [28] used PLS to characterize myocardial infarction using class labels as the response variable and the data matrix as the predictor variables. They found that running the regression with a range of latent factors and combining the estimations with a median operator could obtain better performance. In the current paper, logistic regression was used (instead of PLS in [28]) with the class labels as the response variable, because this is a commonly used clinical tool to examine associations with disease, and it is simple to calculate relative effects of the components on the response variable as odds ratios. The current paper also differs from [28] in the use of PLS to derive orthogonal remodelling components and the finding that a single latent factor reduces correlations in the resulting remodelling scores.

The results also show that clinically derived components quantitatively characterise remodelling features associated with myocardial infarction with similar power as PCA components. Three logistic regression models based on the clinical indices, PCA components and orthogonal remodelling components derived from clinical indices were all similar in

terms of goodness of fit. Significance tests on areas under the ROCcurves (AUC) revealed that the one-factor PLS model showed significantly greater AUC compared with the multi-factor PLS model and the clinical indices model, but not significantly different from the PCA model. Hence the single latent factor remodelling components characterised myocardial infarction similarly to PCA, while having the added advantage of having clear clinical interpretation with respect to their corresponding clinical indices, as well as being an orthogonal decomposition of shape space. R2.18

Coefficients of the remodelling components estimated in the logistic regression model were projected back on the population shape space. Figure 7 visualises the shape changes characterizing presence of disease. This combined component can be used for tracking individual patients over time in future studies, by quantifying the degree to which their LV shapes compare with the remodelling spectrum.

In this study, we included all of the available cases (1,991 asymptomatic and 300 myocardial infarction), since we were primarily interested in the proof of concept. Having a balanced data set is preferable to enable the analysis of differences between "asymptomatic remodelling" and "symptomatic remodelling", which would be of considerable interest in terms of physiological driving factors. However, Figure 5b indicates that over 1000 cases would be required for robust identification of remodelling components. Also, physiological functions between different pathological groups can be quite different. For example, comparing the remodelling components of 1991 asymptomatic subjects only with remodelling components of 1991 asymptomatic + 300 myocardial infarction revealed differences of 9.1 degrees in EDVI, 6.4 degrees in sphericity, 15.1 degrees in EF, 7.0 degrees in RWT, 9.5 degrees in conicity and 8.4 degrees in longitudinal shortening. Hence, the myocardial infarction patients, which were only 24% from all samples, had a significant influence on all the remodelling components. R2.20 R3.3

Supervised feature extraction techniques such as information maximising component analysis and linear discriminate analysis have also been used to extract a remodelling component which can best characterize myocardial infarction using surface sampling [29]. In the current study, the shape features of each clinical index were obtained first and then combined using logistic regression. The shape changes due to myocardial infarction obtained by this logistic regression model can be more easily explained as a combination of well-understood shape features, through the logistic regression coefficients.

This method can be applied to any index with particular clinical utility, with visualization of their corresponding shape features and quantification of components, thereby further exploiting shape information in a clinically meaningful fashion.

Limitations R2.1

The cross-sectional nature of these data limits the understanding that can be gained on the physiological factors underlying remodelling processes. However, the methods developed in this work can be applied to future studies to track patients over time, or to epidemiological studies such as the Multi-Ethnic Study of Atherosclerosis [30] and the UK Biobank [31]. We R2.3
also limited the clinical remodelling indices examined in this paper to those geometric indices which have been well established in the clinical literature. These indices are also readily available from several imaging modalities such as 3D echo and CT. The order the indices are R2.7 R3.4
included in the basis has an effect on the resulting remodelling components. While we used the variance of the corresponding remodelling scores (a measure of shape variance explained), other methods are possible and this requires further research. Finally, we did not include structural information on the location and size of the infarct. While more information is R2.3 R2.11
becoming available on the interesting effects of infarct size and transmurality, this is left for

future work. Also, many patients have comorbidities such as valvular disease, which was not examined in the current study.

Potential implications

An orthogonal decomposition of shape in relation to remodelling indices of known prognostic value will enable multi-dimensional characterization of the ways in which the heart adapts with the progression of disease, e.g. after myocardial infarction. The remodelling components R3.5 R2.10 were able to characterize disease as well as standard methods, with the added advantages of having clear clinical interpretation with respect to their corresponding clinical indices, as well as being an orthogonal decomposition of shape space. The resulting remodelling scores can be used to track the progression of remodelling over time, against reference populations. This would enable automatic computation of z-scores giving precise information on how the patient's heart compares against the reference population (in this case the MESA cohort). Although the remodelling components were generated from a largely asymptomatic R2.8 R3.5 population in this work, we showed how they describe the shape changes undergone in myocardial infarction relatively well. We also showed how the amount of each remodelling component could be quantified in association with the presence of clinical disease, highlighting significant contributions of ventricular size, sphericity and relative wall thickness. These methods enable new knowledge to be derived from medical imaging examinations on the underlying mechanisms driving the adaptation of the heart in response to disease. Future work can also examine how the remodelling scores are related to future adverse events, e.g. using clinical outcomes for MESA which are known over a >10 year follow up period.

Availability of supporting data and materials

All data and results are available from www.cardiacatlas.org. The data are not publically available due to IRB restrictions on the contributing studies; however, data are made available on approval of a research application submitted under the Cardiac Atlas Project data sharing policy (www.cardiacatlas.org).

Declarations

Abbreviations and Acronyms

Left ventricular =LV， Ejection Fraction = EF, Principal Component Analysis = PCA, Partial Least Squares = PLS, End-diastolic Volume Index (EDVI), Myocardial infarction =MI，Ejection fraction =EF， Longitudinal shortening =LS， Relative wall thickness = RWT, Systolic Blood Pressure=SBP, Diastolic blood pressure=DBP

Ethics approval and consent to participate

This study was approved by the local institutional review boards (Johns Hopkins University School of Medicine NA_00031350; Northwestern University CR1_STU00000078; New Zealand Multi-region Ethics Committee MEC/08/04/052) and all participants gave written informed consent.

Consent for Publication

Not applicable

Competing interests

None

Funding

This project was supported by award numbers R01HL087773 and R01HL121754 from the National Heart, Lung, and Blood Institute. MESA was supported by contracts N01-HC-95159 through N01-HC-95169 from the NHLBI and by grants UL1-RR-024156 and UL1-RR-025005 from NCRR. DETERMINE was supported by St. Jude Medical, Inc; and the National Heart, Lung and Blood Institute (R01HL91069). David A. Bluemke is supported by the NIH intramural research program. Xingyu Zhang was supported by the China Scholarship Council.

Authors' contributions

All authors were involved in the design of the study, interpretation of the data, drafting and revision of the manuscript, and final approval of the submitted manuscript. XZ, PM-G, and AS performed the statistical analyses.

Authors' Information

XZ is a biostatistician. PM-G is a biostatistician and expert in bioinformatics. BA-V is a bioengineer and expert in medical image analysis. DB is a radiologist and Director of Radiology and Imaging Sciences at the National Institute of Biomedical Imaging and Bioengineering and a co-PI of the MESA study. BR is a clinical engineer and an expert in cardiac MRI. JPF is a radiologist and Director of Magnetic Resonance Research at UCLA and a co-PI of the DETERMINE study. AK is a cardiologist and PI of the DETERMINE study; DL is a cardiologist and Director of the DETERMINE MRI Core Lab. JL is a cardiologist and Director of the MESA MRI Core Lab. AY is a bioengineer and PI of the Cardiac Atlas Project and head of Department of Anatomy and Medical Imaging at the University of Auckland. AS is an expert in atlas-based medical image analysis.

Appendix

R1.1
R1.13
R1.17

Principal Component Regression

Let $\boldsymbol{X} \in \mathbb{R}^{N \times P}$be a data matrix of predictor variables where each row is a case (shape vector) and each column a shape feature (in our case [x y z] coordinates of sampled points). There are N cases and P shape features. We first "column center" the data by subtracting the mean across cases.

Principal Component Analysis (PCA) decomposes $\boldsymbol{X}$ into an othonormal matrix $\boldsymbol{\Phi} \in \mathbb{R}^{P \times M}$ containing eigenvectors of the covariance matrix $\boldsymbol{X}^{\mathrm{T}}\boldsymbol{X}$. The columns of $\boldsymbol{\Phi}$ define "shape components". M is the number of shape components used to approximate $\boldsymbol{X}$, typically M<P, by

$$\boldsymbol{X}_{est} = \boldsymbol{T}\boldsymbol{\Phi}^{T} \tag{A.1}$$

$$\boldsymbol{T} = \boldsymbol{X}\boldsymbol{\Phi} \tag{A.2}$$

where $\boldsymbol{T} \in \mathbb{R}^{N \times M}$ is a matrix of "scores". Each case is thus approximated by a linear combination of shape components. The weights of the combination (rows of $\boldsymbol{T}$) are the amount of each shape component present in that case, and are calculated by projecting each shape vector onto the shape component.

In principal component regression (PCR), the response or dependent variable $\boldsymbol{Y}$ (at present we consider a single response variable being a centered remodeling index such as EDVI) is regressed against the principal component scores (scores being used as predictor variables):

$$\boldsymbol{Y}_{est} = \boldsymbol{T}\boldsymbol{B}_{PCR} \tag{A.3}$$

where $\boldsymbol{B}_{PCR}$ is a vector of regression coefficients.

The advantage of this method is that the regression coefficients do not suffer from the well-known multicolinearity problem, in which the regression coefficients can be ill-defined if the independent variables are correlated, leading to instability in future predictions. Note that in PCA the resulting scores $\boldsymbol{T}$ are orthogonal, so the resulting scores have zero correlation within the dataset between different component scores.

PCR Remodeling Component:

The PCR can be written as

$$\boldsymbol{Y}_{est} = \boldsymbol{T}\boldsymbol{B}_{PCR} = \boldsymbol{X}\boldsymbol{\Phi}\boldsymbol{B}_{PCR} = \boldsymbol{X}\boldsymbol{\beta}'_{PCR} \quad \text{(A.4)}$$

Here $\boldsymbol{X}$ are the predictor variables and the regression coefficients are calculated from the PCR as $\boldsymbol{\beta}'_{PCR} = \boldsymbol{\Phi}\boldsymbol{B}_{PCR}$. This vector of regression coefficients can be thought of as the linear combination of shape components that best predict the response variable. We define a "PCR remodeling component" $\boldsymbol{\beta}^{■}_{PCR}$by normalizing $\boldsymbol{\beta}'_{PCR}$ (note the data and response are centered so we exclude the zero intercept). The PCR remodelling scores are defined as follows:

$$\boldsymbol{Y}_{PCRscore} = \frac{\boldsymbol{X}\boldsymbol{\beta}'_{PCR}}{|\boldsymbol{\beta}'_{PCR}|} = \boldsymbol{X}\boldsymbol{\beta}_{PCR} \quad \text{(A.5)}$$

The remodelling score for each case is then a projection (inner product) of the shape vector on the remodelling component. The remodelling component is defined by analogy to PCA shape components as a unit length direction in shape space. Remodelling scores are defined by analogy to shape scores in PCA; we can get the estimated remodelling index from $\boldsymbol{Y}_{PCRscore}$ by scaling by the norm of $\boldsymbol{\beta}'_{PCR}$ and adding the mean.

Partial Least Squares Regression

A problem with PCR is that the independent variables are chosen by their ability to explain variance in $\boldsymbol{X}$, not $\boldsymbol{Y}$. Partial least squares (PLS) regression solves this problem by finding the

"latent factors" that best explain the covariance between $\boldsymbol{Y}$ and $\boldsymbol{X}$. These are ranked from largest to smallest covariance, so the first factor explains the most covariance, the second factor for the second largest covariance, and so on.

PLS finds a linear decomposition of $\boldsymbol{X}$ and $\boldsymbol{Y}$ such that

$$\boldsymbol{X} = \boldsymbol{T}\boldsymbol{\Psi}^T + \boldsymbol{E}_X \quad \text{(A.6)}$$

$$\boldsymbol{Y} = \boldsymbol{U}\boldsymbol{\Omega}^T + \boldsymbol{E}_Y \quad \text{(A.7)}$$

where $\boldsymbol{T} \in \mathbb{R}^{N\times M}$ and $\boldsymbol{U} \in \mathbb{R}^{N\times M}$ are PLS scores for predictor and response variables, respectively. Similarly, $\boldsymbol{\Psi} \in \mathbb{R}^{P\times M}$ and $\boldsymbol{\Omega} \in \mathbb{R}^{K\times M}$ (K=1 for a single response variable) are the PLS components or loadings for the predictor and response variables. Unlike PCR, $\boldsymbol{\Psi}$ and $\boldsymbol{\Omega}$ are not orthogonal and not normalized. The parameter $M \leq P$ is the number of latent factors, typically determined by examining the percentage variance explained in Y.

PLS derives the $\boldsymbol{\beta}$ regression coefficients as linear combinations of the PLS loadings, which are chosen to be maximally correlated. Several variants exist in the literature, differing in the calculation of $\boldsymbol{T}$ [21, 22]. However, similar to PCR, we can define PLS remodelling components and remodelling scores as

$$\boldsymbol{Y}_{PLSscore} = \frac{\boldsymbol{X}\boldsymbol{\beta}'_{PLS}}{|\boldsymbol{\beta}'_{PLS}|} = \boldsymbol{X}\boldsymbol{\beta}_{PLS} \quad \text{(A.8)}$$

As for PCR, the estimated $\boldsymbol{Y}$ can be derived from the scores by scaling by $|\boldsymbol{\beta}'_{PLS}|$ and adding the mean.

Orthogonal Remodelling Components

The orthogonalization process given in (3) can be applied to the results of PCR or PLS regression. PLS regression is always more efficient than PCA regression, in that fewer terms

are required to capture the variance of the response variable. However, if all PCA components are included in the PCR, and all latent factors in the PLS, the two methods are equivalent. One-factor PLS (ie M=1 in the PLS regression) has particular properties which may make it attractive in some applications. For example one-factor PLS has been shown to be equivalent to rescaled ridge regression as the ridge parameter tends to infinity [22].

For K>1, ie more than one response variable included in Y, the PLS regression finds latent factors which explain the most covariance between the ***X*** and ***Y*** matrices simultaneously. This was not considered for the current work because the resulting regression coefficients are not orthogonal.

References

1. Sutton MGSJ, Sharpe N: **Left Ventricular Remodeling After Myocardial Infarction: Pathophysiology and Therapy.** *Circulation* 2000, **101:**2981-2988.
2. Gjesdal O, Bluemke DA, Lima JA: **Cardiac remodeling at the population level[mdash]risk factors, screening, and outcomes.** *Nat Rev Cardiol* 2011, **8:**673-685.
3. Lieb W, Gona P, Larson MG, Aragam J, Zile MR, Cheng S, Benjamin EJ, Vasan RS: **The Natural History of Left Ventricular Geometry in the CommunityClinical Correlates and Prognostic Significance of Change in LV Geometric Pattern.** *JACC: Cardiovascular Imaging* 2014, **7:**870-878.
4. Zile MR, Gaasch WH, Patel K, Aban IB, Ahmed A: **Adverse left ventricular remodeling in community-dwelling older adults predicts incident heart failure and mortality.** *JACC: Heart Failure* 2014.
5. Wong SP, French JK, Lydon A-M, Manda SOM, Gao W, Ashton NG, White HD: **Relation of left ventricular sphericity to 10-year survival after acute myocardial infarction.** *The American journal of cardiology* 2004, **94:**1270-1275.
6. White HD, Norris RM, Brown MA, Brandt PW, Whitlock RM, Wild CJ: **Left ventricular end-systolic volume as the major determinant of survival after recovery from myocardial infarction.** *Circulation* 1987, **76:**44-51.
7. Di Donato M, Dabic P, Castelvecchio S, Santambrogio C, Brankovic J, Collarini L, Joussef T, Frigiola A, Buckberg G, Menicanti L, the RG: **Left ventricular geometry in normal and post-anterior myocardial infarction patients: sphericity index and 'new' conicity index comparisons.** *European Journal of Cardio-Thoracic Surgery* 2006, **29:**S225-S230.
8. Konstam MA, Kramer DG, Patel AR, Maron MS, Udelson JE: **Left Ventricular Remodeling in Heart FailureCurrent Concepts in Clinical Significance and Assessment.** *JACC: Cardiovascular Imaging* 2011, **4:**98-108.
9. Anand I, McMurray J, Cohn JN, Konstam MA, Notter T, Quitzau K, Ruschitzka F, Lüscher TF: **Long-term effects of darusentan on left-ventricular remodelling and clinical outcomes in the Endothelin A Receptor Antagonist Trial in Heart Failure (EARTH): randomised, double-blind, placebo-controlled trial.** *The Lancet* 2004, **364:**347-354.
10. Kramer CM, Lima JA, Reichek N, Ferrari VA, Llaneras MR, Palmon LC, Yeh IT, Tallant B, Axel L: **Regional differences in function within noninfarcted myocardium during left ventricular remodeling.** *Circulation* 1993, **88:**1279-1288.
11. Jolliffe I: *Principal component analysis.* Wiley Online Library; 2005.
12. Zhang X, Cowan BR, Bluemke DA, Finn JP, Fonseca CG, Kadish AH, Lee DC, Lima JAC, Suinesiaputra A, Young AA, Medrano-Gracia P: **Atlas-Based Quantification of Cardiac Remodeling Due to Myocardial Infarction.** *PLoS ONE* 2014, **9:**e110243.
13. Medrano-Gracia P, Cowan BR, Ambale-Venkatesh B, Bluemke DA, Eng J, Finn JP, Fonseca CG, Lima JAC, Suinesiaputra A, Young AA: **Left ventricular shape variation in asymptomatic populations: the multi-ethnic study of atherosclerosis.** *Journal of Cardiovascular Magnetic Resonance* 2014, **16:**56.
14. Remme EW, Young AA, Augenstein KF, Cowan B, Hunter PJ: **Extraction and quantification of left ventricular deformation modes.** *IEEE Trans Biomed Eng* 2004, **51:**1923-1931.
15. Fonseca CG, Backhaus M, Bluemke DA, Britten RD, Do Chung J, Cowan BR, Dinov ID, Finn JP, Hunter PJ, Kadish AH: **The Cardiac Atlas Project—an imaging database for computational modeling and statistical atlases of the heart.** *Bioinformatics* 2011, **27:**2288-2295.
16. Medrano-Gracia P, Cowan BR, Bluemke DA, Finn JP, Kadish AH, Lee DC, Lima JAC, Suinesiaputra A, Young AA: **Atlas-based analysis of cardiac shape and function: correction of regional shape bias due to imaging protocol for population studies.** *Journal of Cardiovascular Magnetic Resonance* 2013, **15:**80.

17. Allgower EL, Schmidt PH: **Computing Volumes of Polyhedra.** *Mathematics of Computation* 1986, **46:**171-174.
18. Schulz-Menger J, Bluemke DA, Bremerich J, Flamm SD, Fogel MA, Friedrich MG, Kim RJ, von Knobelsdorff-Brenkenhoff F, Kramer CM, Pennell DJ, et al: **Standardized image interpretation and post processing in cardiovascular magnetic resonance: Society for Cardiovascular Magnetic Resonance (SCMR) board of trustees task force on standardized post processing.** *J Cardiovasc Magn Reson* 2013, **15:**35.
19. Li L, Shigematsu Y, Hamada M, Hiwada K: **Relative Wall Thickness Is an Independent Predictor of Left Ventricular Systolic and Diastolic Dysfunctions in Essential Hypertension.** *Hypertension Research* 2001, **24:**493-499.
20. Izumo M, Lancellotti P, Suzuki K, Kou S, Shimozato T, Hayashi A, Akashi YJ, Osada N, Omiya K, Nobuoka S, et al: *Three-dimensional echocardiographic assessments of exercise-induced changes in left ventricular shape and dyssynchrony in patients with dynamic functional mitral regurgitation.* 2009.
21. Geladi P, Kowalski BR: **Partial least-squares regression: a tutorial.** *Analytica chimica acta* 1986, **185:**1-17.
22. de Jong S, Phatak A: **Partial least squares regression.** *Recent advances in total least squares techniques and errors-in-variables modeling* 1997**:**311-338.
23. Gómez-Carracedo MP, Andrade JM, Rutledge DN, Faber NM: **Selecting the optimum number of partial least squares components for the calibration of attenuated total reflectance-mid-infrared spectra of undesigned kerosene samples.** *Analytica Chimica Acta* 2007, **585:**253-265.
24. Zhang X, Ambale-Venkatesh B, Bluemke D, Cowan B, Finn JP, Hundley W, Kadish A, Lee D, Lima JC, Suinesiaputra A, et al: **Orthogonal Shape Modes Describing Clinical Indices of Remodeling.** In *Functional Imaging and Modeling of the Heart. Volume* 9126. Edited by van Assen H, Bovendeerd P, Delhaas T: Springer International Publishing; 2015: 273-281: *Lecture Notes in Computer Science*].
25. Mevik B-H, Wehrens R: **The pls Package: Principal Component and Partial Least Squares Regression in R.** *J Stat Softw* 2007, **18:**1--23.
26. DeLong ER, DeLong DM, Clarke-Pearson DL: **Comparing the areas under two or more correlated receiver operating characteristic curves: a nonparametric approach.** *Biometrics* 1988, **44:**837-845.
27. Robin X, Turck N, Hainard A, Tiberti N, Lisacek F, Sanchez JC, Muller M: **pROC: an open-source package for R and S+ to analyze and compare ROC curves.** *BMC Bioinformatics* 2011, **12:**77.
28. Lekadir K, Albà X, Pereañez M, Frangi AF: **Statistical Shape Modeling Using Partial Least Squares: Application to the Assessment of Myocardial Infarction.** In *Statistical Atlases and Computational Models of the Heart Imaging and Modelling Challenges: 6th International Workshop, STACOM 2015, Held in Conjunction with MICCAI 2015, Munich, Germany, October 9, 2015, Revised Selected Papers.* Edited by Camara O, Mansi T, Pop M, Rhode K, Sermesant M, Young A. Cham: Springer International Publishing; 2016: 130-139
29. Zhang X, Ambale-Venkatesh B, Bluemke D, Cowan B, Finn J, Kadish A, Lee D, Lima J, Hundley W, Suinesiaputra A, et al: **Information maximizing component analysis of left ventricular remodeling due to myocardial infarction.** *Journal of Translational Medicine* 2015, **13:**343.
30. Bild DE, Bluemke DA, Burke GL, Detrano R, Diez Roux AV, Folsom AR, Greenland P, Jacob DR, Jr., Kronmal R, Liu K, et al: **Multi-Ethnic Study of Atherosclerosis: objectives and design.** *Am J Epidemiol* 2002, **156:**871-881.
31. Petersen SE, Matthews PM, Bamberg F, Bluemke DA, Francis JM, Friedrich MG, Leeson P, Nagel E, Plein S, Rademakers FE, et al: **Imaging in population science: cardiovascular magnetic resonance in 100,000 participants of UK Biobank - rationale, challenges and approaches.** *J Cardiovasc Magn Reson* 2013, **15:**46.

Tables

Table 1 Demographics and clinical remodelling indices for asymptomatic subjects and patients with myocardial infarction (mean ± SD). MI=Myocardial infarction; BMI=Body mass index; SBP=Systolic blood pressure; DBP=diastolic blood pressure; EDVI= end diastolic volume index; RWT=relative wall thickness; EF= ejection fraction; LS=longitudinal shortening. R1.43

Variable	Unit	Asymptomatic	MI cases	p-value
Sex	F/M	1034/975	60/238	<0.01
Age	years	61.47±10.15	62.76±10.76	0.043
Height	cm	165.98±9.99	173.82±9.77	<0.001
Weight	kg	76.75±16.50	90.06+14.14	<0.001
BMI		27.77±5.09	29.73+5.57	<0.001
SBP	mmHg	126.28±21.98	126.36±17.50	>0.05
DBP	mmHg	71.49±10.33	73.26±9.82	0.006
Diabetes history	%	13.11	35.67	<0.001
Smoking status	%	12.51	11.33	>0.05
EDVI		67.83±13.29	96.53±25.03	<0.001
Sphericity		0.38±0.08	0.41±0.09	<0.001
RWT	%	39.71±9.49	35.21±8.38	<0.001
Conicity		0.74±0.08	0.70±0.08	<0.001
EF		0.63±0.07	0.41±0.11	<0.001
LS		0.13±0.04	0.08±0.03	<0.001

Table 2 Correlation coefficients between the clinical indices and the PLS remodelling component scores (M=1). EDVI= end diastolic volume index; RWT=relative wall thickness; EF= ejection fraction; LS=longitudinal shortening.

	EDVI score	Sphericity score	EF score	RWT score	Conicity score	LS score
EDVI	0.82	0	0	0	0	0
Sphericity	0.03	0.83	0	0	0	0
EF	-0.75	0.03	0.61	0	0	0
RWT	-0.20	-0.16	-0.04	0.53	0	0
Conicity	-0.14	-0.28	0.30	0.21	0.72	0
LS	-0.45	0.03	0.61	-0.17	0.20	0.53

Table 3 Correlation coefficients between the clinical indices and the PLS remodelling component scores (M=10). EDVI= end diastolic volume index; RWT=relative wall thickness; EF= ejection fraction; LS=longitudinal shortening.

	EDVI score	Sphericity score	EF score	RWT score	Conicity score	LS score
EDVI	0.94	0.27	-0.34	-0.64	-0.13	-0.31
Sphericity	0.30	0.97	-0.15	-0.16	-0.25	-0.13
EF	-0.41	-0.28	0.90	0.22	0.25	-0.02
RWT	-0.65	-0.12	0.26	0.99	0.25	0.53
Conicity	-0.13	-0.22	0.38	0.25	0.97	0.24
LS	-0.32	-0.13	0.02	0.56	0.25	0.98

Table 4 Correlation coefficients between the clinical indices and the first 6 PCA shape components. EDVI= end diastolic volume index; RWT=relative wall thickness; EF= ejection fraction; LS=longitudinal shortening. R1.49

	PC 1	PC 2	PC 3	PC 4	PC 5	PC 6
EDVI	0.80	-0.01	-0.74	-0.18	-0.13	-0.45
Sphericity	-0.26	-0.80	0.19	0.19	0.30	0.06
EF	-0.01	0.09	-0.11	0.03	-0.09	-0.20
RWT	0.10	0.24	-0.21	-0.25	-0.25	-0.18
Conicity	0.10	0.13	-0.15	-0.11	-0.15	-0.14
LS	0.21	0.02	0.03	-0.15	0.50	0.37

Table 5 Correlation coefficients among the clinical indices. EDVI= end diastolic volume index; RWT=relative wall thickness; EF= ejection fraction; LS=longitudinal shortening.

	EDVI	Sphericity	EF	RWT	Conicity	LS
EDVI	1	0.28	-0.60	-0.37	-0.11	-0.29
Sphericity	0.28	1	-0.11	-0.28	-0.22	-0.13
EF	-0.60	-0.11	1	0.18	0.26	0.57
RWT	-0.37	-0.28	0.18	1	0.32	0.00
Conicity	-0.11	-0.22	0.26	0.32	1	0.26
LS	-0.29	-0.13	0.57	0.00	0.26	1

Table 6 Correlation coefficients among the PLS remodelling scores (M=1). EDVI= end diastolic volume index; RWT=relative wall thickness; EF= ejection fraction; LS=longitudinal shortening.

	EDVI score	Sphericity score	EF score	RWT score	Conicity score	LS score
EDVI score	1	-0.29	-0.15	0.22	-0.15	-0.08
Sphericity score	-0.29	1	0.001	-0.04	0.01	0.22
EF score	-0.15	0.001	1	0.09	0.09	0.47
RWT score	0.22	-0.04	0.09	1	-0.08	0.002
Conicity score	-0.15	0.01	0.09	-0.08	1	0.16
LS score	-0.08	0.22	0.47	0.002	0.16	1

Table 7 Correlation coefficients among the PLS remodelling scores (M=10). EDVI= end diastolic volume index; RWT=relative wall thickness; EF= ejection fraction; LS=longitudinal shortening.

	EDVI score	Sphericity score	EF score	RWT score	Conicity score	LS score
EDVI score	1	0.29	-0.68	-0.37	-0.15	-0.34
Sphericity score	0.29	1	-0.17	-0.15	-0.25	-0.14
EF score	-0.68	-0.17	1	0.27	0.25	0.53
RWT score	-0.37	-0.15	0.27	1	0.31	-0.01
Conicity score	-0.15	-0.25	0.25	0.31	1	0.24
LS score	-0.34	-0.14	0.53	-0.01	0.24	1

Table 8 Four logistic regressions for myocardial infarction. EDVI= end diastolic volume R2.14
index; RWT=relative wall thickness; EF= ejection fraction; LS=longitudinal shortening.

Variable	Coefficient	Standard error	p value	Standardized Coefficient	Odds Ratio(OR)	OR 95% Confidence Interval	
Model 1: PCA shape components + Baseline variables							
**PC 1**	**2.644**	**0.177**	**<.0001**	**1.455**	**14.066**	**9.942**	**19.901**
**PC 2**	**-0.605**	**0.102**	**<.0001**	**-0.334**	**0.546**	**0.447**	**0.666**
PC 3	0.071	0.112	0.524	0.039	1.074	0.863	1.336
**PC 4**	**2.031**	**0.153**	**<.0001**	**1.111**	**7.625**	**5.652**	**10.287**
**PC 5**	**0.391**	**0.106**	**<.0001**	**0.215**	**1.478**	**1.200**	**1.821**
PC 6	-0.113	0.119	0.342	-0.062	0.893	0.708	1.127
Model 2: Clinical indices + Baseline variables							
**EDVI**	**0.041**	**0.008**	**<.0001**	**0.412**	**1.042**	**1.027**	**1.058**
Sphericity	0.002	0.014	0.870	0.010	1.002	0.975	1.030
**EF**	**-0.164**	**0.015**	**<.0001**	**-0.966**	**0.849**	**0.825**	**0.874**
RWT	0.002	0.014	0.875	0.012	1.002	0.975	1.030
**Conicity**	**-0.037**	**0.016**	**0.018**	**-0.161**	**0.963**	**0.934**	**0.994**
**LS**	**-0.148**	**0.037**	**<.0001**	**-0.325**	**0.862**	**0.802**	**0.927**
Model 3: PLS remodelling scores (M=1) + Baseline variables							
**EDVI score**	**2.859**	**0.191**	**<.0001**	**1.574**	**17.444**	**11.997**	**25.365**
**Sphericity score**	**0.895**	**0.125**	**<.0001**	**0.492**	**2.446**	**1.915**	**3.124**
**EF score**	**-1.540**	**0.148**	**<.0001**	**-0.846**	**0.214**	**0.160**	**0.287**
**RWT score**	**-1.289**	**0.146**	**<.0001**	**-0.710**	**0.275**	**0.207**	**0.367**
**Conicity score**	**0.331**	**0.124**	**0.007**	**0.181**	**1.392**	**1.093**	**1.774**
LS score	-0.041	0.140	0.769	-0.023	0.960	0.729	1.263
Model 4: PLS remodelling scores (M=10) + Baseline variables							
**EDVI score**	**0.823**	**0.161**	**<.0001**	**0.454**	**2.277**	**1.661**	**3.120**
Sphericity score	-0.189	0.114	0.098	-0.103	0.828	0.662	1.036
**EF score**	**-1.843**	**0.180**	**<.0001**	**-1.016**	**0.158**	**0.111**	**0.225**
RWT score	0.087	0.128	0.495	0.048	1.091	0.849	1.403
**Conicity score**	**-0.393**	**0.122**	**0.001**	**-0.216**	**0.675**	**0.531**	**0.858**
**LS score**	**-0.665**	**0.141**	**<.0001**	**-0.365**	**0.514**	**0.390**	**0.678**

All the models are adjusted for age, gender, BMI, DBP, smoking status and diabetes history. Bold rows indicate $p<0.05$.

Table 9 Comparison of the four logistic regression models. AIC = Akaike information criterion ; BIC =Bayesian information criterion; AUC =Area under the ROC curve. Smaller Deviance, AIC and BIC, and larger AUC, are indicative of better goodness-of-fit. Bold row indicates best performance. R1.47

	Deviance	AIC	BIC	AUC
Baseline	1560	1574	1615	0.7415
Indices	710	727	802	0.9594
PCA scores	607	633	708	0.9725
**PLS scores (M=1)**	**569**	**595**	**669**	**0.9739**
PLS scores (M=10)	683	709	784	0.9598

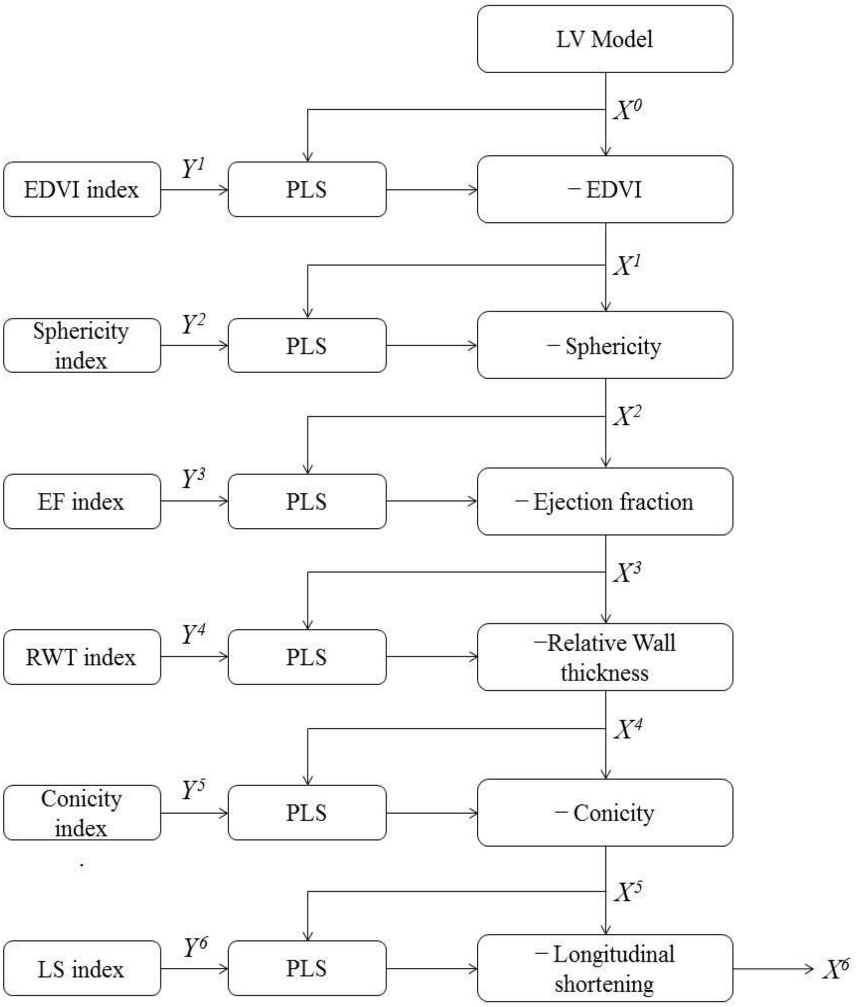

Figure 1 Data processing flow chart. LV=left ventricle; X = shape space; Y = response variable; PLS = partial least squares; EDVI= end diastolic volume index; RWT=relative wall thickness; EF= ejection fraction; LS=longitudinal shortening.

 Click here to download Figure Figure 2.docx

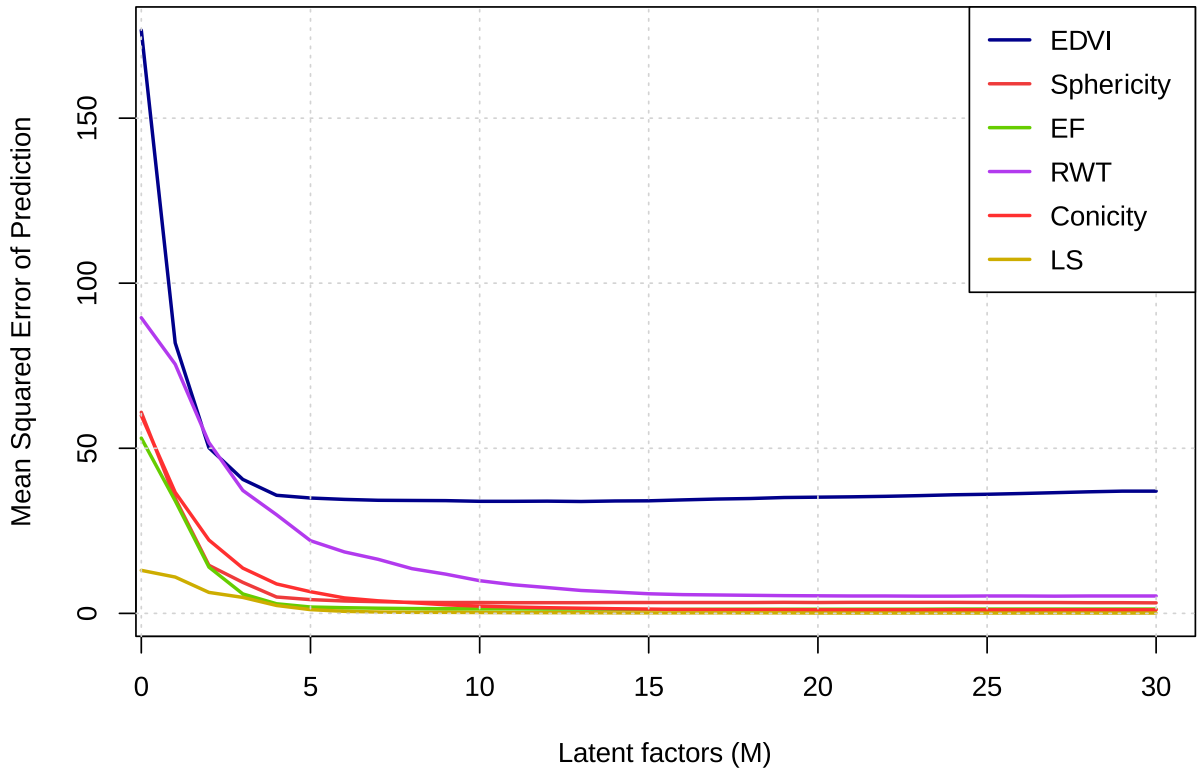

Figure 2. Mean squared error predictions of PLS regression coefficients using different number of latent factors (M). 10-fold cross validations were applied. EDVI= end diastolic volume index; RWT=relative wall thickness; EF= ejection fraction; LS=longitudinal shortening.

 Click here to download Figure Figure 3.docx

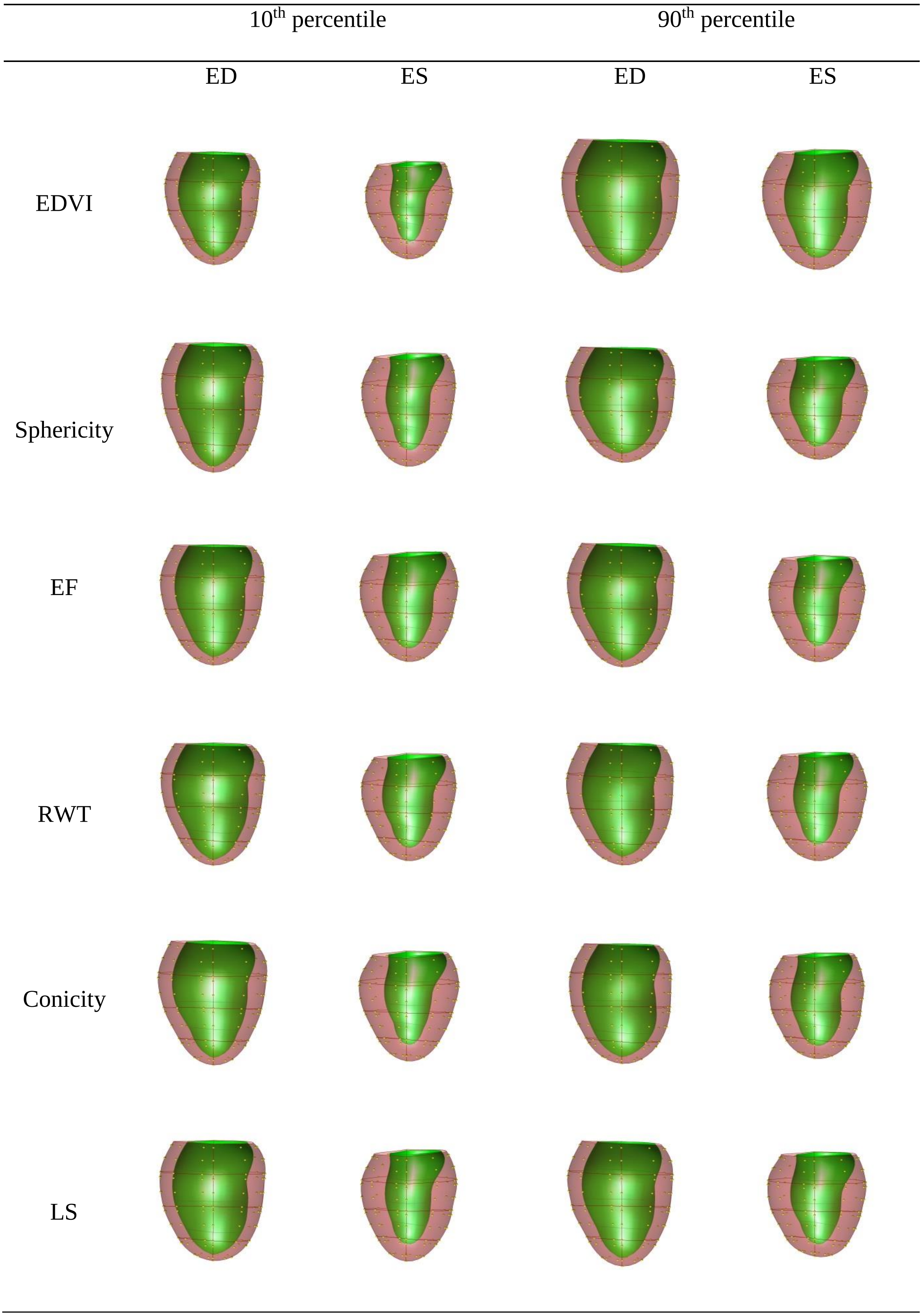

Figure 3. Plot of the PLS clinical components (*M* =1). EDVI= end diastolic volume index; RWT=relative wall thickness; EF= ejection fraction; LS=longitudinal shortening. ED = end-diastole; ES = end-systole. Full animations of each clinical component are shown http://www.cardiacatlas.org/tools/lv-shape-orthogonal-clinical-modes/.

Figure 4

Click here to download Figure Figure 4.docx

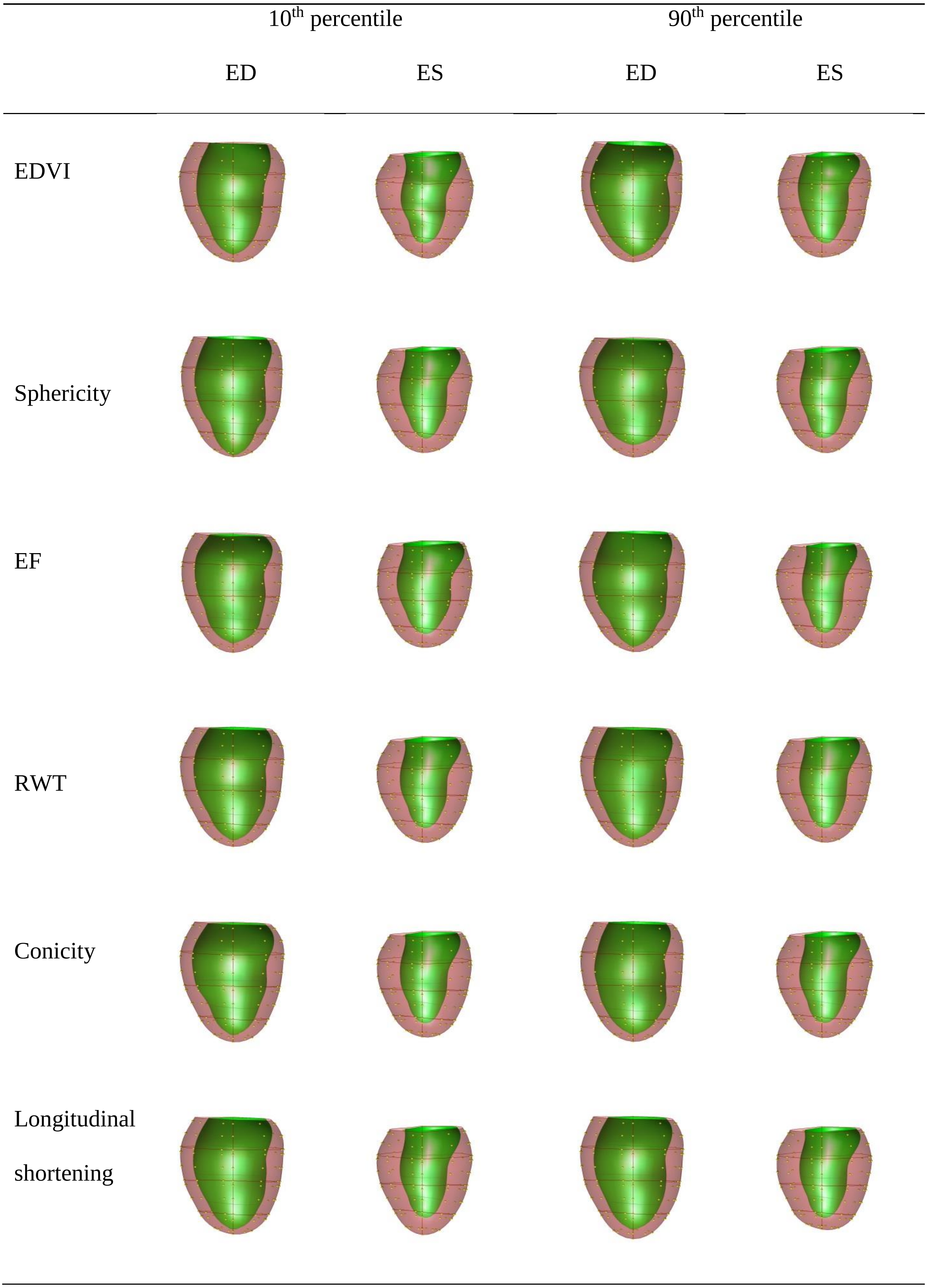

Figure 4. Plot of the PLS clinical components (*M*=10). EDVI= end diastolic volume index; RWT=relative wall thickness; EF= ejection fraction; LS=longitudinal shortening. ED = end-systole; ES = end-diastole.

Figure 5 Click here to download Figure Figure 5.docx

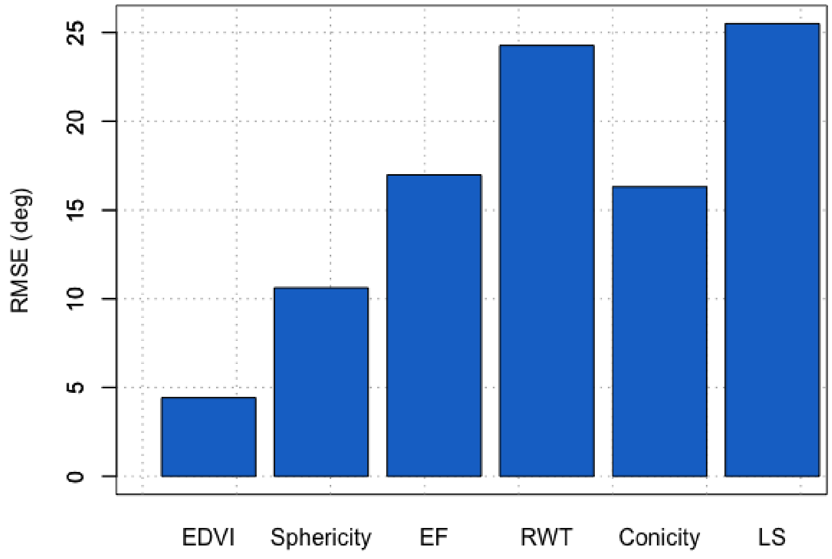

(a) Root mean squared errors between randomly sampled balanced data sets (300 ASYMP and 300 MI) and full data set (1991 ASYMP and 300 MI). Average of 50 trials.

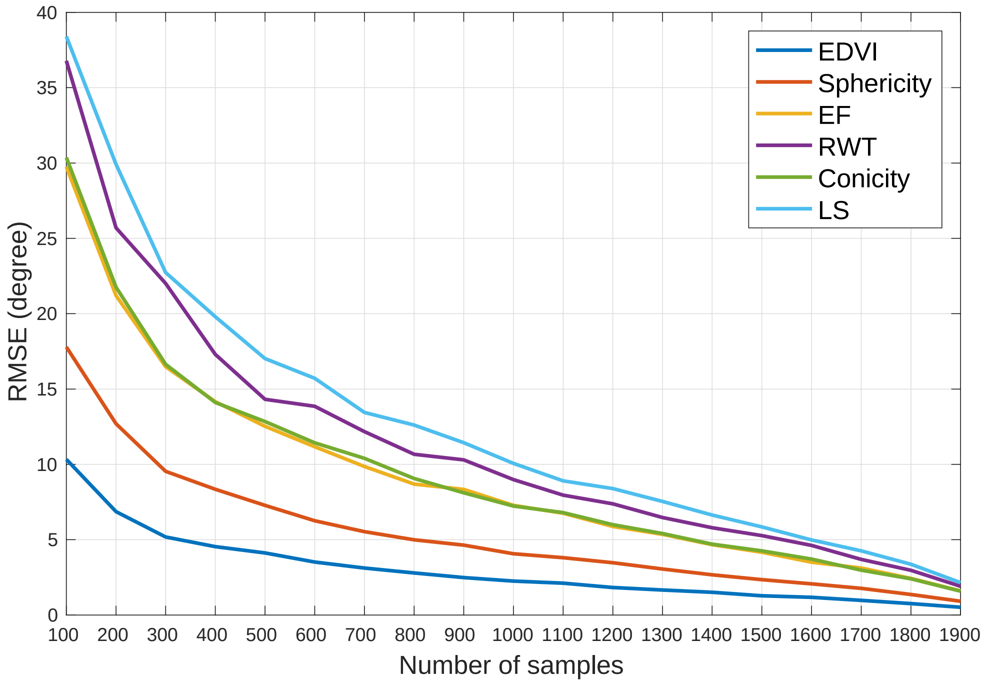

(b) Root mean squared errors varying number of asymptomatic subjects compared with the full data set (1,991 samples). Average of 50 trials.

Figure 5. Root mean squared error (RMSE) in terms of angle differences between remodelling components. EDVI= end diastolic volume index; RWT=relative wall thickness; EF= ejection fraction; LS=longitudinal shortening.

Click here to download Figure Figure 6.docx

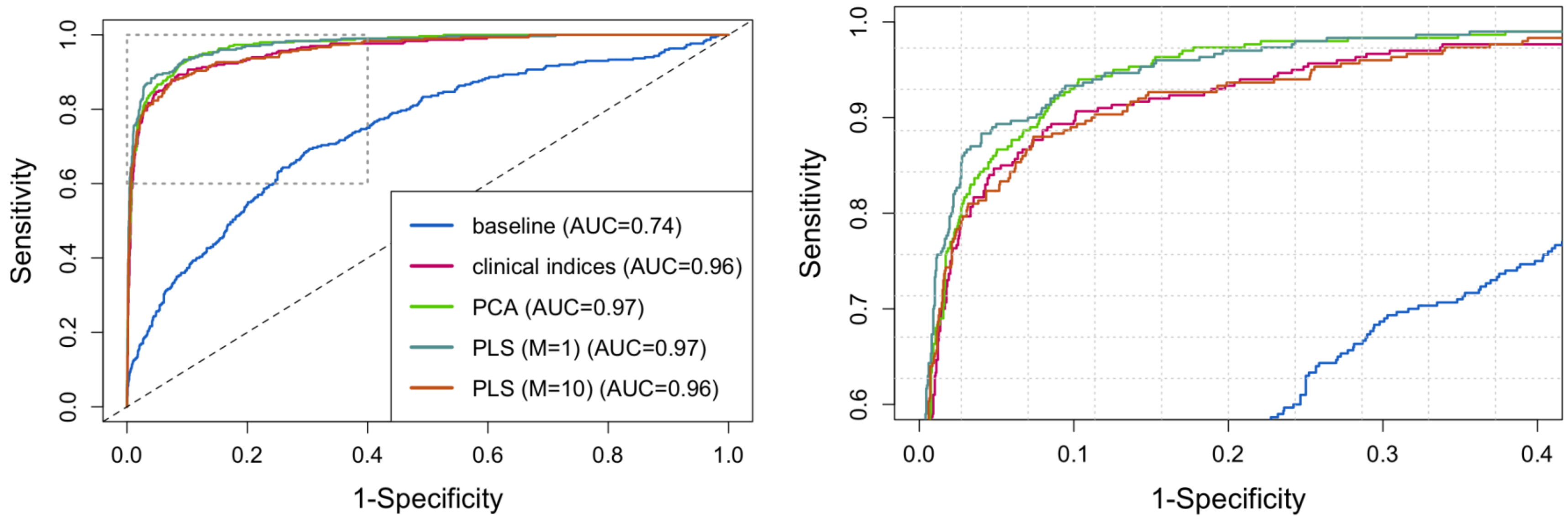

Figure 6. ROC curves for the five logistic regression models. The right figure shows a zoomed-in view to demonstrate the differences between the four models. ROC= reciever operating curve; PCA = principal component analysis; PLS = partial least squares.

Figure 7 Click here to download Figure Figure 7.docx

Asymptomatic		MI Patients	
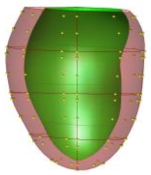	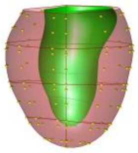	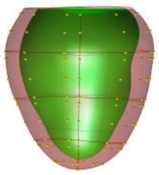	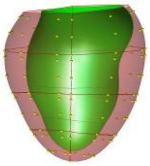
ED	ES	ED	ES

Figure 7. Visualization of shape changes between asymptomatic volunteers and MI patients, using the combined PLS (*M*=1) components. Plots show the shapes associated with the median logistic regression score for the asymptomatic and MI patient groups respectively. MI patients show larger ventricles, less ejection, and thinner walls. MI= myocardial infarction; ED = end-diastole; ES = end-systole.

Dear Nicole
Please find our revision of this paper attached. We appreciate the large effort of the reviewers and we believe the manuscript has improved considerably as a consequence. Here is a detailed response to the reviewers. In the revision, we have marked up the changes for clarity and included a reference number (e.g. R1.1 for reviewer 1, point 1, etc) by each. Since there are a lot of changes, reviewers can search for each point to find where the manuscript has changed.
Regards
Alistair

Reviewer #1: General comments:
I appreciate the large effort from this group to share data and code to help advance progress in the research community. It is also nice to see analysis of large populations.

Overall, I find the manuscript well written and concise. However, some of the methods and motivation are still unclear to me, and due to this I have some major concerns with the methodology and results, as summarized and further detailed below.

R1.1

My main concern with this work is with the methods. Some of the results are not consistent with my experience with PLS (and SIMPLS). Based on looking at the code, it seems that the 'pc_scores' that are computed in 'GenerateOrthogonalModes.m' are actually the prediction of Y and not the 'scores' T. I believe this could be the reason why there are unusual results for the variance of the 10-component model plotted in Fig. 4, because the incorrect scores were used (the PCTVAR output of plsregress should be what is plotted).

We have redefined the terminology to distinguish clearly between “shape components” (i.e. PCA shape components or PLS XLOADINGS returned by MATLAB’s plsregress function) and our “remodelling components” which we define to be the normalized vector in shape space calculated from the regression coefficients. Similarly we distinguish between “shape scores” (i.e. XSCORES returned by plsregress), response scores (YSCORES from plsregress), and our “remodelling scores” which we define to be the projection of the shapes onto the remodelling component. We have included an Appendix to clarify this formulation, using both PCA regression and PLS regression to illustrate the method. Using centered data X and response vector Y, we show that the normalized regression coefficients can be thought of as a vector in shape space (column space of X) such that the projection of the shape onto this vector best explains the response. We call this vector the “remodeling component” by analogy to PCA shape components, which are also vectors in shape space. The difference is that the remodelling component is more directly related to the response variable (i.e. clinical remodelling index in our application). In the appendix, we first derive PCA shape components

and PCA scores. We then show how PCA regression can be used to calculate remodelling components (by analogy to shape components) and remodelling scores (analogous to PCA scores). The remodelling components derived from PCA regression are linear combinations of the PCA shape components. Then we show how PLS regression can be used to calculate (different) remodelling components. Here the remodelling components are derived from both XLOADING and YLOADING in plsregress. For a given number of latent factors in PLS, and the same number of principal components in PCA, remodelling components derived from PLS regression always explain more of Y than remodelling components derived from PCA regression.

The orthogonalization part of our algorithm can then be applied to either PCA or PLS remodelling components, by subtracting the projection of the shapes onto the remodelling component (giving a residual data matrix), in sequence. This procedure gives an orthogonal set of remodelling components, each related to their associated clinical indices, but forming an orthogonal basis for the shape space. By analogy to PCA shape components, these orthogonal remodelling components can be used as a shape decomposition, but unlike PCA shape components, the orthogonal remodelling components have clear clinical interpretation in terms of clinical remodelling indices.

We have also changed the names of some variables in the code to be consistent with this terminology.

For discussion on Figure 4 see point 1.31 below

R1.2

In addition, there is a strong emphasis placed on the computed latent variables being "de-correlated". In my experience, when one computes PLS for a given factor, the first component will maximise the covariance between X and Y, but not 100%, meaning that subsequent shapes will also have some correlation with other Y - e.g. EDVi score has -0.75 correlation with EF, so this shape does not seem 'de-correlating' at all (if I understand what the authors mean by 'de-correlating'. In fact, usually ~10 components still capture some correlation with Y. Removing the first component that was computed to maximise covariance with e.g. EDV will remove some amount of EDV-related shape, but not ALL of it, which is what seems to be implied from the phrasing used in the manuscript. Therefore, despite the fact that the model with 10 latent variables yielded lower performance, it seems more "de-correlating" than the model with 1 latent variable, because the shape features related to the first variable have been more "completely" removed. However, my intuition is that removing the first 10 EDV-related shapes probably removes most of the variability of the shape from the population, since within those shapes there are some features that are also related to the other variables. So, I would think that a 1-component method is more suitable with this approach.

We have revised the terminology to clarify that our orthogonalization method

using one-factor PLS regression gives “less correlated” remodelling scores than using multi-factor PLS (new Tables 6 and 7). Also it leads to “less correlation” between the remodelling scores and their associated remodelling indices (Tables 2 and 3), and “zero correlation” between the scores and the indices associated with previously removed remodelling components. Initially, it was not obvious to us that a single latent factor would lead to less correlated remodelling scores, but we have tested this behavior in several datasets and it appears to be a fundamental result, due to the fact that one-factor PLS is closer to PCA. The deJong paper also mentions a result linking one-factor PLS with ridge regression (included in the Appendix).

We have added a section in the motivation on why it is sometimes desirable to have less correlated remodelling scores, for example if the scores relate to underlying processes, then low correlation between scores implies that the processes have different effects within the population.

R1.3

Regarding the comparison of methods and results, I don't find a convincing improvement of using PLS as opposed to PCA, in terms of accuracy or prediction. I do, however, agree that for interpretability of the results there is added gain of using this method. Therefore, I believe the idea of using PLS is valid, but the motivation for using it needs to be shifted in the paper.

Yes, it is the clinical interpretation of the components that is the main advantage of our method. We have now emphasized this in the Discussion. However, both PLS regression and PCA regression can be used to derive remodelling components. If sufficient principal components are used in PCA regression, and sufficient latent factors in PLS regression, they will give similar accuracy of prediction (and usually similar regression coefficients and therefore remodelling components). However this results in remodelling scores which are more highly correlated. In the logistic regression experiments, we show that one-factor PLS derived remodelling scores are as effective as PCA shape scores in characterizing remodelling in patients, but the advantage of the PLS remodelling components is the interpretation of each component corresponding with its clinical index.

Detailed comments:
Abstract:

R1.4

I am not convinced that a "novel method" is proposed, as stated in the abstract. Perhaps I have misunderstood the methods but they seem to be the same as previously proposed methods using the method described in [24] and applying to the data described and previously analysed in [13]. In my opinion, this work is the application of existing methods to a data-set and should be stated as such.

The method described in [24] used a different method to derive remodelling components. For each clinical index, this previous paper defined a subset of

cases outside two standard deviations of the mean, i.e. those that display very high and very low values of the clinical index. The remodelling mode was then derived from these cases, ignoring the majority of cases between two standard deviations of the mean. The problem with this method is that it relied on extremes of the distribution of the clinical index. This may lead to difficulties in the interpretation of the remodelling component. The novel contributions of the current paper are i) calculation of remodelling components directly from regression coefficients, ii) use of the entire distribution of the clinical index to formulate the remodelling component, and iii) reduction of correlation among resulting remodelling scores, using PLS regressions with a single latent factor. These points have been added to the motivation section.

The dataset used in this work is available from the Cardiac Atlas Project, and has been used in a number of studies including [13] (now ref 12). This is the advantage of having widely-available datasets for algorithm development.

R1.5

What is meant by "a single PLS hidden variable"? I'm perhaps not familiar with this terminology, but is this referring to a single PLS latent variable or single PLS component?

We have changed this to “latent factor” throughout. This refers to the number of components in the PLS decompositions, i.e. the NCOMP parameter of plsregress function for the Matlab version and plsr function for the R version. There are several names for this in the literature, e.g. latent component, latent factor, latent variable, and hidden variable, etc. We chose latent factor to distinguish these from shape components or remodelling components, etc.

R1.6

I also didn't exactly understand what is meant by a "decorrelation between scores". Is this referring to the orthogonalisation of the scores or reduction in the correlation of scores?

See R1.2

Introduction:

R1.7

Is there a difference between "LV volume index" and "LV volume", or is this referring to indexed LV volume? (line 55).

End-diastolic volume index (EDVI) is the EDV divided by body surface area, defined in the Data Description section on Clinical Remodelling Indices.

R1.8

It could be useful for the reader to define what is an orthogonal decomposition

of shape (line 64).

This is now defined in the Appendix. Also we have added more motivation of the usefulness of an orthogonal decomposition in the Background section, which includes the definition of orthogonality.

R1.9

Line 79 - I think it may be more correct to state that PCA components are not designed to be related to clinical factors (though this can be the case). Clinical interpretation is not so much difficult, as it is suboptimal (in fact it is easy using PCR).

We have changed this to read: "However, PCA shape components are not designed to be related to any particular clinical remodelling index, and the clinical interpretation of PCA shape components is often difficult. Previous work showed that, LV PCA shape components did not have clear clinical interpretation beyond the first two [12]. This is a common problem with PCA shape components, since they are designed to efficiently characterize shape variation without regard to possible underlying mechanisms of disease processes."

R1.10

Line 91 - as mentioned above, the term "PLS hidden variable" is unclear to me, could the authors clarify what exactly is meant by this (i.e. what is "hidden")?

Changed to "latent factor", see R1.5.

R1.11

Last sentence page 4 - is this to say that there is no possible relationship between a clinical index and a previous shape? This phrasing "complete decorrelation" seems a bit strong to me.

See R1.2

Methods:

R1.12

General question: I'm curious to know why the authors didn't use the PLS regression coefficients directly since that is what PLS was mainly developed for (e.g. following the tutorial in Matlab on PLSR and PCR). Can the authors mention why they chose logistic regression instead? Was a comparison performed? Did it improve the results? Would we expect a logistic relationship over a linear one? Please clarify.

The logistic regression was used to evaluate the ability of the remodelling components to characterize shape changes due to myocardial infarction. PLS

could be used for this task (and was used for this purpose by Lekadir *et al* in [28]). However we decided to use the more common logistic regression since it is the standard method used in many previous clinical research studies and is it simple to calculate relative strengths of associations using odds ratios. These comments have been added to the *Characterization of myocardial infarction* section.

R1.13

General comment: It would be useful to clarify for the reader (especially those not familiar with latent variable models), what the component, loading, and scores are (i.e. component = loading x score)

These are explained in the Appendix.

R1.14

Line 103 - typo? should it be "heart failure or atrial fibrillation"?

Thanks, fixed.

R1.15

Line 112 - presumably Simpson's rule was applied? A citation here for clarity would be useful.

Actually numerical integration of the polygon formed by the surface points. Citation added.

R1.16

Line 154 - perhaps deflation could be defined here. Deflation is typically used in original PLS algorithms but not SIMPLS, thus it could be nice to differentiate between standard 'deflation' and the orthogonalisation process used here.

We have avoided use of deflation in this context. We have used “residual data matrix” instead.

R1.17

- *N_latent was described before being introduced (page 6).*
- *I think the equation for maximising the covariance between T and U should be added here, and it should be mentioned that this constraint is what distinguishes PLS from, for example, PCA (i.e. this is how the shape modes are computed to maximise the variance in Y).*
- *The formula for B should be provided.*
- *Y_residuals is not defined.*

These concepts are now explained in the Appendix. The relationships between T, U and B are not easy to write in closed form and actually change with particular implementations of PLS, so we have simplified this section.

R1.18

Line 153 - "this step ensures orthogonality" with respect to what? Presumably with respect to B but this is not explicitly stated.

This is now explained in the section next to equation 3.

R1.19

Line 162 - the term "PLS component" is introduced here to refer to the normalised regression coefficients B_i. Please consider another term to avoid confusion e.g. with 'component' as is used in PCA.

See R1.1.

R1.20

Page 8 - why was 10 chosen as the upper limit for the number of latent variables?

We have included a plot of the mean squared prediction error with a 10-fold cross validation (Figure 2). We have added the following to the "*Number of latent factors"* section: "Standard 10-fold cross-validation was performed to test estimation error for multi-factor PLS, showing that 10 latent factors accounted for most of the mean squared error in estimating ***Y*** (Figure 2)."

R1.21

Page 8 - The authors claim that there is no standard method to choose the number of latent variables. Cross-validation could typically be used for this, as mentioned in the Matlab tutorial for PLSR and PCR. For such an investigation it would be nice to compute and plot the leave-one-out or split-half errors for the number of latent variables = 1:299 (number of subjects - 1), and then just the optimal errors could be reported.

See R1.20

R1.22

Line 172 - it could be useful to mention why X^k+1 is orthogonal to B^k.

This has been made more explicit next to equation 3.

R1.23

Line 183 - details on the logistic regression technique and how this was performed could be added (stepwise forward logistic regression? SPSS?).

This is described in the new section about Statistical Analysis.

R1.24

Line 186 - BMI and SBP should be defined here.

Thanks. We have made an explicit definition of these terms, including in each table caption.

R1.25

Line 187 - it would be nice to mention why these were chosen as the baseline variables and why baseline variables were included.

In the original paper, we used covariates commonly used in the literature as baseline variables in the logistic regression models. However, we have now rationalized the choice of baseline variables to those in Table 1 with significant differences between asymptomatic and MI groups. Smoking was also included since this is known to have a significant effect on the heart. The logistic regression experiments were updated accordingly.

R1.26

Line 188 - Why was a 6 component PCA model used? According to [13] this model only represents ~75% of the shape variance in the population.

We used six PCA components in the logistic regression analysis because we only used six remodelling components. Incorporating more components is expected to give better results. However, these results show that with the same number of components, orthogonal remodelling components perform as well as PCA (and the original indices), but with the advantage that the remodelling components have clear clinical interpretation, while maintaining the property of orthogonality.

R1.27

Line 202 - is ESV used without indexing? If not, LVESVi should be used. If yes, why was EDV indexed and not ESV?

We have deleted this, since ESV was not included in Table 1, or in the clinical remodelling indices. This is because ESVI can be inferred from EDVI and EF.

Analyses

R1.28

Line 199 - Please add the statistical significance threshold ($p < 0.05$), or to avoid repetition, just state once at the beginning of this section that statistical significance was set at $p<0.05$.

This has been included in the section about Statistical Analysis.

R1.29

For reproducibility purposes it could help the reader to mention which software (if any) was used to perform the statistical analyses

This has been included in the sections on *Implementation* and *Statistical Analysis*.

R1.30

Line 222 - could the authors elaborate on this sentence, I didn't get what is meant by 'retaining correlation with the index', and why this would be a bad thing

This sentence has been removed for to avoid confusion.

R1.31

Line 226 - I am very surprised to see that only 15% of the shape variance in the population was captured by 6 components from the N=10 model. Again, perhaps I have misunderstood, but my understanding based on the description of the methods is that the 10-component model should have 10-components for EDV, 10 for sphericity, and so on, so there should actually be 10 x 6 = 60 components for this model, and therefore I would expect a much larger amount of the variance to be captured in such a model. Could the authors clarify why this is not the case, or please correct me if I am wrong about the methods.

Figure 4 in the previous manuscript version was calculated as the variance of the remodelling scores, divided by the total variance in the data matrix (trace(X^TX)/(N-1)). This was done because we are using the regression coefficients as the remodelling component, not the PLS components themselves. This result follows from the fact that a single latent factor results in a remodelling component that is influenced by variance in X as well as Y. However this is peripheral result and, for clarity, we have removed this figure in the revised manuscript to avoid confusion with variance explained returned from PLS regression algorithm which can be either for predictor or response variables.

R1.32

Line 246 - presumably 'LR' stands for logistic regression? Could you add this to the text and figures

We have removed the acronym for logistic regression for clarity.

R1.33

Line 246 - why was the median chosen? Please mention briefly here.

Median shapes were estimated since this is more robust to outliers in general. However in this case mean shapes give similar results.

R1.34

Line 250 - how are the baseline variables adjusted? Does this significant change the shapes? (This question is more out of curiosity than actually needing clarification)

This means that the baseline variables were included in the logistic regression models as covariates. LR models are often termed “adjusted” by these covariates.

Discussion

R1.35

Line 266 - as mentioned previously, I would rather state that an orthogonal PLS framework was applied, without implying that there are new methods proposed in the present work. Again, if this is not the case, please clearly describe the contributions of the present work and distinguish how this method differs from other orthogonal PLS methods.

See R1.4 for an explanation of the novel contributions of the paper.

R1.36

Line 273 - orthogonality was described here, but should also be mentioned at the beginning of the methods section.

This is now defined in the second paragraph of the *Background* section.

R1.37

Line 274 - I got a bit lost here with the terminology, are the "PLS shape components" referring to loading x score or are you referring to the loading (which I guess is the case because PLS loadings are orthogonal)? And presumably "PLS shape component score" is referring just to the scores (which are not necessarily orthogonal for PLS)? Here there is also the mention of the term 'decorrelated', should that be 'orthogonal'?

We have rationalized the terminology- see R1.1 and R1.2.

R1.38

Line 284 - there is again the use of 'decorrelation' and I just now think I understand what is meant by this. Perhaps "reduction/decrease in correlation" is clearer?

See R1.2.

R1.39

Line 285 - I'm honestly very surprised to see "total decorrelation" (and again, I would suggest using "zero correlation" rather than "decorrelation") between the PLS scores and clinical indices. Indeed this suggests that the 1-component model is able to remove any relationship with EDVi (for the second component), and so on.

We have changed this to zero correlation as suggested.

Results

R1.40

In all results (and tables, figures) it would be useful to clarify when experiments are including both populations and when it is MI only, sometimes I got confused by that.

All experiments and Tables show results including all cases (both asymptomatic and MI patients), unless explicitly stated.

R1.41

I'm not sure how to interpret the results. Are the authors looking for the most predictive model? In that case I would expect to see a more thorough analysis of the number of latent variables (using cross-validation).

The logistic regression experiments were performed to examine the ability of the orthogonal remodelling components to characterize shape changes between patients with MI and asymptomatic volunteers, compared with the same number of PCA components, or the original clinical indices themselves. The interpretation of the results is that orthogonal remodelling components are able to characterize differences between groups with similar metrics to PCA or the original indices, but with the added advantage of having a clear clinical interpretation and maintaining orthogonality. We use the AIC etc as metrics of "goodness" in this context with respect to traditional PCA shape components. The question of how many latent factors is a separate issue, and we show that one-factor PLS remodelling components perform better than multi-factor PLS in the logistic regression experiments in all metrics (Table 9). We have also included a cross-validation to show that 10 factors is a reasonable choice for the multi-factor case, in terms of prediction of the response variables, but this is another issue again.

R1.42

Do the authors have some reasoning for why LS score was significant with the 1-component model and not the 10-component model, and vice versa for conicity?

This was a typo, thanks. The results have changed somewhat with the revised logistic regression analyses (DBP was include as a covariate rather than SBP). The one-factor model shows different significant scores from the multi-component model. We believe this is due to the increased multi-colinearity in the multi-factor model.

Tables

R1.43

In all tables it would be useful to include the abbreviations

We have included all abbreviations in all table captions.

R1.44

The tables are in general very content-heavy, and it's not easy to see what the take-home message is from each table. Some additional annotations or descriptions in the legend would help guide the reader to interpret these results. For example, the statistically significant components in Table 8 could be highlighted for easy readability, rather than using an asterix.

Comments on the interpretation of results have been added to the Results text where the Tables are cited. We have used bold in Table 8 as suggested.

R1.45

Are Tables 2-7 showing results for the MI population only or are these combined results for both populations (please specify in the legends).

Unless otherwise specified all Tables show results including all cases combined. This is now made explicit at the beginning of the Results section.

R1.46

In Table 8 it would be useful to include some descriptions of what are "good" values in terms of the coefficient, error, OR and CI.

These have standard interpretation in the clinical literature, and depend on the units of measurement. In general, higher means more influence on the dependent variable.

R1.47

Table 9 is a nice summary of the results and easy to interpret. Line 195 could

be repeated here to remind the reader what is preferred for each measure (e.g. >AIC = better)

Done.

R1.48

Table 5 and 6 - it is not clear what is meant by 'PLS clinical mode scores' and how this is different from 'PLS component scores'.

This has been rationalized, see R 1.1

R1.49

Table 7 should be moved to follow Tables 2,3 for easier readability.

Table 7 has been shifted to become Table 4.

Figures

R1.50

In all figures it would be helpful to include the abbreviations

Done.

R1.51

Figure 1 is nice and clear. If possible, it could be useful to include on the left-hand side an image depicting each measure or the formula for computing each measure, and on the right-hand side the corresponding modes at +1SD. X6 could be pointing downwards for consistency

We thought this would clutter the figure and make it less readable. This information is repeated in Figure 3 and in the text.

R1.52

I don't find Figure 2 and Figure 3 very informative in the sense that I don't know what I should conclude from these images. Perhaps some annotation could help as guidance. It would be nice to have some interpretation and comparison of the modes in Figure 2 and 3 to the modes in Figure 14 of [13] in terms of highlighting for the reader regions of interest or interesting behavior that is visible from these modes (i.e. what should we, as readers, take from these Figures?)

These figures visualize the remodelling components associated with the clinical indices. These visualizations are useful in understanding the effect of each component on shape. This explanation has been added to the text near first mention of the figures. Animations of these remodelling components from the smallest and to the highest percentiles can clearly visualize how these components are associated with the clinical indices. The animations for the single latent factor are shown interactively on the Cardiac Atlas Project

website: http://www.cardiacatlas.org/tools/lv-shape-orthogonal-clinical-modes/. We added this link to the figure caption.

R1.53

The labels on the x-axis of Fig. 4 are a bit misleading. I would rather put 'PC1' directly below the blue column, and EDVI PC below the red/green columns (since PC1 in PCA is not related to EDVI, or am I mistaken?).

This figure has been removed. See R1.31.

R1.54

Figure 4 - I am very confused by these results, especially for the first component. To my understanding, in both the 1-component and 10-component models, PLS was performed with the same X shape features and EDVI as the Y variable. There is no tuning of SIMPLS to force all of the variance to be in the N-components, therefore the variance of the first component should be equal, regardless of the number of components that was chosen. The number of chosen components changes the accuracy of the regression, but not the components themselves. Therefore, the variance of the first component should be much higher than what is reported for the 10-component model. While there would be large differences in the subsequent components (because there is much fewer variance in the other components for the 10-component model because so much of the shape has already been removed from X^k), the first component should be identical to the 1-component model (i.e. 50%). Please clarify why this is not the case.

Hopefully, this it should now be clear that Fig 4 was plotting variance in remodelling scores. However, to avoid confusion, we have removed this Figure (see R1.31).

R1.55

Figure 5 - The improvement from baseline alone is clear (and expected) but I don't see a dramatic improvement based on the figures for the shape-based models and using clinical indices alone. Moreover, there isn't a clear improvement above PCA. Figure 5- could the authors add AUC (as reported on line 239) to the figure?

The ROC curves are now in Figure 6. We have added the AUC values in the legend. We tested whether the AUC values of single and multi latent factor PLS models are significantly different from the PCA and clinical index models. The test showed that AUC of the single latent factor PLS model is significantly greater than using clinical indices alone, but not different to the PCA model. However, the multi latent factor PLS was significantly smaller than PCA, but it was not significantly different than clinical index model. The interpretation of this Figure is that the single latent factor (M=1) orthogonal remodelling components give similar performance to PCA, but with the added advantage of clear clinical interpretation of the components. These observations have

been added to the Discussion section.

R1.56

Figure 6 is interesting. Perhaps the author could consider adding some annotation to guide the reader about the shape differences (e.g. there seems to be less systolic contraction in the MI patients) and a summary of what to conclude in the legend (even a repetition of line 249 would be helpful here).

Done.

Tools:

R1.57

For the sake of this journal (being focused towards open-source tools),I would suggest that the authors use R (https://cran.r-project.org/web/packages/pls/index.html) instead of Mxatlab, to avoid the need for users to purchase a Matlab license. Using the plsregress function also requires a license for the Statistics and Machine Learning Toolbox.

I am not familiar with Giga science, but based on the website it is stated that all research objects are published (data, software tools, and workflows). In order to reproduce the results from this study (or indeed to apply the methods to new data), the community would need to have access to the image processing tools that were used to extract the models. PCA (or similarly PLS) applied to data that has already been extracted and parameterised is straightforward using existing software (or indeed using built-in Matlab, python, or R functions). While it is a useful resource to have access to the images and the models extracted from these images, the biggest challenge we face in the field is in creating the models to be able to perform the analysis.

We have included code in R in the GitHub repository, which is linked from http://www.cardiacatlas.org/tools/lv-shape-orthogonal-clinical-modes/ webpage.

Code for creating the shape models is provided at http://www.cardiacatlas.org/tools/. This code is offered as is where is, and we have not been able to ensure that the dependencies are available.

Novelty:

R1.58

As mentioned previously, to my knowledge this technique has already been described in [24] and there is inadequate referencing to previous techniques. Orthogonalisation using the Gram-Schmidt method has been discussed earlier, for example Izenman, A.J., 2008. Modern multivariate statistical techniques (Vol. 1). New York: Springer, page 570), and for PLS specifically: de Jong, S., Wise, B.M. and Ricker, N.L., 2001. Canonical partial least squares and continuum power regression. Journal of Chemometrics, 15(2), pp.85-100. Moreover, the Matlab code for canonical (i.e. orthogonal) SIMPLS

is provided in this paper.

See R1.4. The De Jong 2001 paper shows how to derive PLS regression from a canonical decomposition, which is not the same as the orthogonal remodelling components derived in our paper.

Code:

R1.59

It isn't clear to me why the regression coefficients ('Beta') are normalised in 'GenerateOrthogonalModes' and subsequently why the scores and loadings from the plsregress function are not used directly. Could the authors explicitly mention why this normalisation is important.

See R1.2. Normalized regression coefficients (without the intercept) give rise to a unit vector in shape space which can be used as a component similar to shape components in PCA.

R1.60

*As mentioned previously, from what I understand from the code, the 'pc_scores' are computed as X*B (data matrix X times the regression coefficients). However, this is the model of Y, not the computation of the scores. The scores T would usually be computed by projecting X onto the loadings P.*

See R1.2. Remodelling scores can be used to calculate estimates of Y but are also scores associated with remodelling components.

Reviewer #2:

R2.1

General: The strength of this paper is the novel mathematical process of making decoupled geometrical modes, while still correlating with clinical indices, and the main limitations is that the study is cross sectional and as such limited understanding can be gained on what really drives the remodeling. The paper is missing a limitation section where the lack of cross sectional data is highlighted and the need for such future research is discussed.

We have included a Limitations section which includes the cross-sectional nature of the dataset, and applications to other datasets.

R2.2

Abstract: A novel method for deriving orthogonal shape components directly from any set of clinical indices. The ***word any is a strong word*** *given the mathematical depth of the paper. For instance, the clinical indices need to be reasonably well uncorrelated for the operation to be meaningful and produce shape components that do correlate with the chosen clinical indices.*

We agree, although what constitutes “reasonably uncorrelated” is difficult to define and is beyond the scope of the current paper. This is likely to depend

on the application and might be a matter of trial and error. We have chosen remodelling indices which are common and moderately independent (e.g. we did not include ESV since EDV and EF had larger variance). The abstract has been revised to read “We developed a novel method for deriving orthogonal *remodelling components* directly from any (moderately independent) set of clinical remodelling indices.”

R2.3

Abstract. Why is not infarct size one of the clinical indices? Likely, it must be stronger than for instance longitudinal shortening to determine remodeling?

We did not include structural indices such as % infarct mass or infarct transmurality or age in this work, since we wanted to focus on geometric remodelling indices which have been well established in the clinical literature. These indices are also available from several imaging modalities such as 3D echo and CT. While more information is becoming available on the interesting effects of infarct size and transmurality, this requires explanation of specific methodological techniques and is left for future work. We have included these comments in the Limitations section.

R2.4

Page 3, Line 67. In this section you may lose some of the potential readers of the paper. I do understand and acknowledge that it greatly simplifies that any given metric tensor does not have off diagonal elements and is orthonormal ideally. However, is this really a practical limitation as in order to compute measures such as arc lengths and areas it simple to reconstruct the original shape of the patient and compute them directly in the Euclidean space? Well it is more computationally intensive, but it is more convenience rather than anything else?

We have removed this sentence for simplicity. We have also included more motivation of the utility of orthogonal components (see R1.2; R3.5).

R2.5

Page 5, line 114. The definition of relative wall thickness is rather strange, I presume that this is the form where previous researchers got significant correlations for prognostics for this parameter, but it would be good to have some more rationale on why this rather bizarre formulation, and why not for instance absolute mean wall thickness in mm (or even minimal wall thickness from thinned after myocardial infarction etc).

The echo community has used this definition for many years since it is easy to measure from an M-mode parasternal view. Many papers have used this as a prognostic measure, and to quantify concentric vs eccentric remodelling. All the remodelling indices in this paper were defined as ratios to correct for scale in some sense. We have included more rationale for the selection of clinical remodelling indices in the Data Description section.

R2.6

Page 6, line 119. Some more details would be good here. Is it the basal AV plane movement divided by the straight distance to apex or is it divided by the curve length? Central basal point is this the middle point of the mitral valve?

This sentence has been modified to: "Longitudinal shortening was calculated as the difference of the distance between the centroid of the most basal ring of points to the most apical point at end-systole divided by the distance at end-diastole."

R2.7

Page 7, Line 156. How was the next component to be removed from the shape space determined? Greatest variance in what respect?

Remodelling components were removed in order of variance of the remodelling scores. This is a measure of the shape variance explained by each index. There could be several methods for determining the order of the indices, and this requires further research. This has been added to the *Orthogonal Remodelling Components* section and the *Limitations* section.

R2.8

Page 11, line 252. This paragraph is somewhat important as I understand it in terms of possible application of the technology. This section could perhaps be better explained and expanded as it deals with how the shape decomposition can be used to derive new knowledge.

We have added the following to the Potential Implications section: "Although the remodelling components were generated from a largely asymptomatic population in this work, we showed how they describe the shape changes undergone in myocardial infarction relatively well. We also showed how the amount of each remodelling component could be quantified in association with the presence of clinical disease, highlighting significant contributions of ventricular size, sphericity and relative wall thickness. These methods enable new knowledge to be derived from medical imaging examinations on the underlying mechanisms driving the adaptation of the heart in response to disease."

R2.9

Please when introducing new abbreviations such as LR help write them out in the text. Is it correct that LR in this context it is logistic regression?

We have removed this abbreviation for clarity.

R2.10

Page 14, line 306. The concept of tracking patients over time with shape decompositions should be highlighted better as this is a rather new concept at least to clinicians and how then such changes can be better understood given orthogonal bases. Please expand somewhat if possible.

We have added the following to the Potential implications section: "The resulting remodelling scores can be used to track the progression of

remodelling over time, against reference populations. This would enable automatic computation of z-scores giving precise information on how the patient's heart compares against the reference population (in this case the MESA cohort)."

R2.11

Page 14, potential implications. Myocardial infarction is a rather broad category in terms of location, and transmurality of the infarct. Furthermore, nowhere throughout the paper it is discussed other causes of remodeling such as valvular disease. As I understood from the description of the normals they did not have valvular disease, but it is rather likely that the infarct patients had such comorbidities.

We have added the following to the Limitations section: "While more information is becoming available on the interesting effects of infarct size and transmurality, this is left for future work. Also, many patients have comorbidities such as valvular disease, which was not examined in the current study."

R2.12

Page 21. Table 1. What is the "old" of the myocardial infarction, i.e how long was it between myocardial infarction and imaging. This may be highly important since that if all are fresh infarctions (< months), then rather little remodeling may have occurred such as limited wall thinning in the infarcted area etc. It is very acute then you have myocardial edema etc as well.

Most patients had stable long term myocardial infarction (none of the patients were acute). We have added this to the Patient Data section.

R2.13

*Page 22, Table 2, it is maybe worth commenting on in the text that LS and RWT achieves rather low correlations compared to their clinical indices. This is even visible in Figure 2, where the 90th percentile of LS does not really show much influence on longitudinal shortening. In fact, as I understand it as the correlation is about 0.5, then this shape mode do only explain 25% (0.5*0.5=0.25) of longitudinal shortening is this correct? How meaningful are really correlations below say 0.7(=> 50% explaining power)?*

Yes, the Pearson correlation coefficients can be low and still be significant due to the large numbers of cases. The question of clinical meaning is an open area of research. Treatments that give a small improvement in remodelling may lead to large cost savings.

R2.14

Page 25, Table 8. What is meant with the baseline model? I find the baseline model poorly described in the paper, please provide more details.

For clarity these have been changed to "baseline variables" throughout. In the original paper, we used covariates commonly used in the literature as baseline variables in the logistic regression models. However, we have now

rationalized the choice of baseline variables to those in Table 1 with significant differences between asymptomatic and MI groups. Smoking was also included since this has a significant effect on the heart. The logistic regression experiments were updated accordingly. The baseline variables are listed in the Characterization of Myocardial Infarction section. These are age, sex, body mass index, diastolic blood pressure, smoking status and diabetes history.

R2.15

Figure 1. Is the order of the indices a design choice or is it based on data? Please expand the legend. See also comment 6.

The order of the indices is important, and we chose the order of the amount of shape variance explained by each remodelling component. This was calculated from the variance of the remodelling scores. See R2.7

R2.16

Is it not strange that given the order EDVI, Sphericity, EF, RWT, Conicity, LS index that the correlations in Table 2 are not dropping in that order, or is this not necessary and rather reflects underlying correlation (or lack thereof) of the clinical indices. If possible, please expand on this.

We believe that the interdependence between clinical indices is a determinant of the decreasing diagonal correlations in Table 2. Thus, RWT and LS are related to indices previously removed by the orthogonalization process (RWT is related to EDVI and sphericity, LS is related to EF). They generally decrease with more components, but they don't need to be monotonic. This has been added to the Results section.

R2.17

Figure 4, is it possible to choice grayscale or colors that works when printed on a grey scale printer.

This figure has been removed, since it was secondary to the main message of the paper (see R1.31).

R2.18

Figure 5, the legend does not describe what is really tested (the decompositions) power to tell if a given patient has in infarction or not? Correct? What is here meant with baseline?

The ROC curves (now shown in Figure 6) measure the ability of the logistic regression model to characterize presence of disease, based on the remodelling components and the baseline variables. Significance tests have now been added. The baseline variables were included because they were different between cohorts and may act as covariates. This is now explained in the *Characterization of myocardial infarction* section.

R2.19

What is the stability of the suggest method? You used the SIMPLS algorithm as implemented by Mathworks, would this change with another algorithm? Are there fundamental differences in possible solutions? Either perform some experiments or discuss this theoretically.

We implemented the computations in R and compared the remodelling components obtained with SIMPLS with kernel, wide kernel and classical orthogonal scores algorithms, and the results were very similar in the regression coefficients obtained.

R2.20

The other factor that would influence the choice of subject population. Here you have 300 infarct patients and some 2000 "normals". Would you get to the same decomposition if you used another set of infarct patients and normal as well as another ratio between normals and patients? This could be tested by taking a sub-population of the input data and perform the computations and compare how these two decompositions coincide in some suitable measure. This would significantly strengthen the paper as the paper describes a rather generic approach to shape decompositions.

We ran a series of experiments with 300 patients and 300 asymptomatic volunteers, with 50 random samples (trials). The root mean squared errors (RMSE) between the resulting remodelling components and those found using all cases (expressed as an angle in degrees calculated from the dot product between the vectors) are shown in Figure 5a. Although the first remodelling component is similar, increasing differences can be seen in the other components. This was expected since the characteristics of the cases included in the training set have an influence on the results. We also looked at the remodelling components generated from the asymptomatic cases alone, increasing the size in the sample from 100 to 1900 (50 trials each). The RMSE with respect to the full 1991 asymptomatic dataset are shown in Figure 5b. This graph shows that we need about 1100 cases to get below 10 degrees in all components. The choice of training data depends on the application. In this paper we used all the available data to generate the remodelling components, since we were primarily interested in the proof of concept. In future work a balanced dataset of more than 1000 cases in each group would be ideal. This would enable calculation of the differences between “asymptomatic remodelling” and “symptomatic remodelling”, which would be of considerable interest in terms of physiological driving factors. These results and comments have been added to the Results and Discussion sections.

Reviewer #3: This paper presents an approach to extract new shape indices from asymptomatic and infarcted ventricles such that they are orthogonal and have high prediction capability. The paper is well written and can be of interest to the statistical cardiac modeling community.

Comments/Questions:

R3.1

PLS has already been used for myocardial infarction classification by Lekadir et al. in STACOM 2015. The authors should cite this paper and describe the differences between the two works.

We have added the following to the Discussion: “Previous studies have also used PLS to derive information on cardiac remodelling [28]. Lekadir et al [28] used PLS to characterize myocardial infarction using class labels as the response variable and the data matrix as the predictor variables. They found that running the regression with a range of latent factors and combining the estimations with a median operator could obtain better performance. In the current paper, logistic regression was used (instead of PLS in [28]) with the class labels as the response variable, because this is a commonly used clinical tool to examine associations with disease, and it is simple to calculate relative effects of the components on the response variable as odds ratios. The current paper also differs from [28] in the use of PLS to derive orthogonal remodelling components and the finding that a single latent factor reduces correlations in the resulting remodelling scores.”

R3.2

What is the difference between calculating the PLS indices based on the clinical indices (EDVI, sphericity, etc) instead of directly using the class labels (asymptomatic vs. Infarcted) as in Lekadir et al. STACOM 2015? The authors should compare the extracted PLS scores through the two methods and see if there are indeed differences.

See 3.1. The focus of the current paper was to derive orthogonal remodelling components based on clinical remodelling indices. We found PLS to be useful in this goal. For the examination of relative effects of these remodelling components on the presence of disease we preferred to use the more common logistic regression analysis.

R3.3

The authors used an imbalance dataset to train the PLS models (300 abnormal vs. about 2000 healthy cases), which may affect the significance of the new shape indices. It would be good to verify if data imbalance affects or not the extraction of the new shape indices. I suggest that the authors run the same experiments with the 300 infarcted cases and 300 randomly selected asymptomatic cases and compare the results.

See R2.20. The choice of training data depends on the application. In this paper we used all the available data to generate the remodelling components.

We have included experiments showing that different components are generated using different training data.

R3.4

It would have been interesting to have a method that finds automatically the best order in the calculation of the PLS score, may be using some statistical criteria, instead of the ad hoc order used in the manuscript (i.e. EDVI, sphericity, EF, etc). What happens if you start with wall thickness for example, which is more directly linked to myocardial infarction?

Yes, this would be a useful area of future research. We have included this in the Limitations section.

R3.5

What is the clinical meaning of the extracted PLS indices? How can they be used by clinicians? Can you show some figures illustrating the variation induced by these indices and their clinical meaning? How do these indices describe better remodeling or infarction than standard clinical indices?

We have included more motivation in the Background section and also expanded the Potential implications section. The main advantage of the remodelling scores generated by the proposed method is that they have clear clinical interpretation with respect to their corresponding clinical indices, as well as being an orthogonal decomposition of shape space.
