# Supplementary material for: Orthogonal decomposition of left ventricular remodeling in myocardial infarction
Source: Gigascience. 2017 Feb 6;6(3):1–15. doi: 10.1093/gigascience/gix005 (PMC5791439; doi:10.1093/gigascience/gix005)
Supplement: GIGA-D-16-00049_Revision_2.pdf [file gix005_giga-d-16-00049_revision_2.pdf]

Manuscript

Click here to download Manuscript Orthogonal PLS - GigaScience - Clean.docx

Orthogonal Decomposition of Left Ventricular Remodelling in Myocardial Infarction

Xingyu Zhang[1], MHS, Pau Medrano-Gracia[1], PhD, Bharath Ambale-Venkatesh[2], PhD, David A Bluemke[3], MD, Brett R Cowan[1], MBChB, J Paul Finn[4], MD, Alan H Kadish[5], MD, Daniel C Lee[5], MD, Joao AC Lima[2], MD, Alistair A Young[1], PhD, Avan Suinesiaputra[1], PhD.

[1] Department of Anatomy and Medical Imaging, University of Auckland, Auckland, New Zealand,

[2] The Donald W. Reynolds Cardiovascular Clinical Research Center, The Johns Hopkins University, Baltimore, USA.

[3] National Institute of Biomedical Imaging and Bioengineering, Bethesda, Maryland, USA,

[4] Department of Radiology, UCLA, Los Angeles, USA.

[5] Feinberg Cardiovascular Research Institute, Northwestern University Feinberg School of Medicine, Chicago, USA

Abstract

Background: Left ventricular size and shape is important for quantifying cardiac remodelling in response to cardiovascular disease. Geometric *remodelling indices* have been shown to have prognostic value in predicting adverse events in the clinical literature, but these often describe interrelated shape changes. We developed a novel method for deriving orthogonal *remodelling components* directly from any (moderately independent) set of clinical remodelling indices.

Results: Six clinical remodelling indices (end-diastolic volume index, sphericity, relative wall thickness, ejection fraction, apical conicity and longitudinal shortening) were evaluated using cardiac magnetic resonance images of 300 patients with myocardial infarction, and 1,991 asymptomatic subjects, obtained from the Cardiac Atlas Project. Partial least squares (PLS) regression of left ventricular shape models resulted in *remodelling components* that were optimally associated with each remodelling index. A Gram–Schmidt orthogonalization process, by which remodelling components were successively removed from the shape space in the order of shape variance explained, resulted in a set of orthonormal remodelling components. *Remodelling scores* could then be calculated which quantify the amount of each remodelling component present in each case. A one-factor PLS regression led to more de-coupling between scores from the different remodelling components across the entire cohort, and zero correlation between clinical indices and subsequent scores.

Conclusions: The PLS orthogonal remodelling components had similar power to describe differences between myocardial infarction patients and asymptomatic subjects as principal component analysis, but were better associated with well-understood clinical indices of cardiac remodelling. The data and analyses are available from www.cardiacatlas.org.

Keywords: cardiac remodelling, magnetic resonance imaging, shape components, partial least squares regression.

Background

Left ventricular (LV) remodelling refers to the process by which the heart adapts its size, shape and function in response to disease processes, or under the influence of mechanical, neurohormonal and genetic factors [1]. Remodelling can be compensatory, for example increased concentric hypertrophy in hypertension, or adverse, for example increased end-systolic volume after myocardial infarction. Adverse LV remodelling characteristics after myocardial infarction provide important diagnostic and prognostic information for the therapeutic management of disease progression [2-5]. Clinical studies have identified quantitative geometric parameters (termed *clinical remodelling indices* in this paper) that describe recognised clinical patterns of remodelling with prognostic value for predicting adverse events. For example, increased LV end-diastolic volume index (EDVI) has been shown to be an important predictor of mortality after myocardial infarction [6]. Increased LV sphericity has also been linked with decreased survival [5]. Relative LV wall thickness [1] and apical conicity [7] are also important indices of adverse remodelling after myocardial infarction. Functional parameters such as ejection fraction (EF), which is the most common index of cardiac performance in clinical practice, are also heavily influenced by the degree of LV remodelling [8, 9]. LV longitudinal shortening is another sensitive marker of LV functional remodelling [10].

Although these clinical remodelling indices have validated prognostic value, they are often coupled so that it is difficult to separate the relative effects on heart shape. For example, end-diastolic volume is often correlated with EF in patients with myocardial infarction. It is therefore difficult to tease out the relative effects of dilatation (structural) from contraction (functional). In computational shape analysis, it is desirable to characterize the space of possible heart shapes in terms of orthogonal shape components. A shape component is a unit vector in shape space, and orthogonal components have zero dot product between different

components. An orthogonal decomposition of heart shape, in which each component is related to a remodelling index with clear clinical importance, would assist clinical interpretation of the relative effects of different physiological processes underlying the development of disease. In addition, such an orthogonal decomposition would enable computational analysis of each component of remodelling present in various forms of heart disease. In particular, an orthogonal basis for shape enables robust calculation of the contribution of each component independently to the overall shape. Also, regressions using orthogonal shape components as independent variables do not suffer from the problem of multicolinearity. Thus, when analysing the combined effects of different remodelling characteristics, it is preferred to have an orthogonal basis in a linear space.

Principal component analysis (PCA) [11] is a powerful and widely used shape analysis technique that provides an orthogonal linear shape basis. In previous work, PCA analysis of LV geometry has achieved more powerful descriptions of LV shape, and their relationships with risk factors, than traditional mass and volume analysis [12]. In a large population study, the first and second PCA LV shape components were associated with LV size and sphericity respectively [13]. However, PCA shape components are not designed to be related to any particular clinical remodelling index, and the clinical interpretation of PCA shape components is often difficult. Previous work has shown that LV PCA shape components do not have clear clinical interpretation beyond the first two [12]. This is a common problem with PCA shape components, since they are designed to efficiently characterize shape variation without regard to possible underlying mechanisms of disease processes. Remme *et al.* [14] developed a method to decompose shape changes into modes with clear clinical interpretation. However, these modes were not orthogonal.

Decomposition of the shapes into orthogonal components enables calculation of scores as projections of each patient's shape onto the corresponding component (see Appendix). These

scores quantify the amount of each shape component present in the patient's heart. One advantage of PCA shape components is that the resulting scores have zero correlation across the population (see Appendix). This is desirable in some applications, i.e. if the scores can be related to underlying processes, then low correlation between scores implies that the processes have different effects within the population.

Previously, orthogonal remodelling components were generated from clinical remodelling indices using an ad hoc approach [24]. For each clinical index, a subset of cases was chosen outside two standard deviations from the mean, i.e. those with very high and very low values of the clinical index. The remodelling component was then derived from these cases, by fitting a line between the two groups. The problem with this method is that it relies on extremes of the distribution of the clinical index and ignores the majority of cases. This may lead to difficulties in the interpretation of the remodelling component. Therefore, the current paper sought to provide the following novel contributions: i) calculation of remodelling components directly from regression coefficients, ii) use of the entire distribution of the clinical index to formulate the remodelling component, and iii) reduction of correlation among resulting remodelling component scores.

In this paper, we used partial least squares (PLS) regression to sequentially construct an orthogonal shape decomposition that is optimally related to clinical remodelling indices. Clinical remodelling indices of EDVI, sphericity, EF, relative wall thickness, conicity and longitudinal shortening, known from the literature to have important prognostic information in the management of myocardial infarction, were used to create corresponding orthogonal shape components. By using a single PLS latent factor per clinical index, the resulting component scores were less correlated with each other, and had zero correlation with those clinical indices previously removed.

Data Description

Patient Data

LV shape models of 300 patients with myocardial infarction and 1,991 asymptomatic study subjects were obtained through the Cardiac Atlas Project [15]. The cohort data have been described previously [12] [16] and are available from the Cardiac Atlas Project (http://www.cardiacatlas.org). Briefly, myocardial infarction patients (n=300, age 31−86, mean age 63, 20% women) had clinical history of myocardial infarction with EF>35% and infarct mass >10% of LV myocardial mass. All had stable myocardial infarction (i.e. no acute cases). Asymptomatic subjects (n=1991, age 45−84, mean age 61, 52% women) did not have physician-diagnosed heart attack, angina, stroke, heart failure or atrial fibrillation, and had not undergone procedures related to cardiovascular disease, at the time of recruitment [12] [16].

Finite element shape models were customized to cardiac MRI exams in each case using a standardized procedure [12]. The shape models were evenly sampled on the epicardial and endocardial surfaces at sufficient resolution to capture all shape features, which resulted in 1,682 Cartesian (x, y, z) points in homologous anatomical locations for each LV model.

Clinical Remodelling Indices

Clinical remodelling indices included EDVI, EF, relative wall thickness, sphericity, apical conicity and longitudinal shortening. Volumes were calculated by the summation of surface triangle volumes [17]. LV mass was calculated by subtracting endocardial from epicardial volumes multiplied by 1.05 g/ml [18]. EDVI was calculated as endocardial surface volume at end-diastole (EDV) divided by body surface area. EF was calculated as (EDV-ESV)/EDV, where ESV is the endocardial surface volume at end-systole. Relative wall thickness was defined as twice the posterior wall thickness divided by the end-diastolic diameter [19] at

mid-ventricle. Sphericity was calculated as the EDV divided by the volume of a sphere with a diameter corresponding to the major axis at end-diastole in LV long axis view [20]. Apical conicity was calculated as the ratio of the apical diameter (defined as the diameter of the endocardium one third above the apex) over the basal diameter [7] at end-diastole. Longitudinal shortening was calculated as the difference of the distance between the centroid of the most basal ring of points to the most apical point at end-systole divided by the distance at end-diastole. These indices were not intended as a comprehensive list and were limited to geometric indices (i.e. ratios which correct for size in some sense), which have either been studied for many years (e.g. relative wall thickness as a measure of concentric versus eccentric hypertrophy), or can be readily calculated from several different imaging modalities (e.g. 3D echocardiography, MRI, or CT). Attempts were made to only include indices that are moderately independent (e.g. end-systolic volume index was not included since it can be derived from end-diastolic volume index and EF).

Remodelling Components

In this paper, we use partial least squares (PLS) regression [21, 22] to explain each response variable (remodelling index) $\boldsymbol{Y} \in \mathbb{R}^{N\times 1}$ with a linear combination of predictor variables (LV surface points) $\boldsymbol{X} \in \mathbb{R}^{N\times P}$, so that

$$\boldsymbol{Y} = \boldsymbol{X}\boldsymbol{\beta}' + \boldsymbol{E}_Y \quad (1)$$

where $\boldsymbol{\beta}' \in \mathbb{R}^{P\times 1}$ is a vector of regression coefficients and $\boldsymbol{E}_Y$ is the residual vector. In this paper, the dimensions N and P denote the number of cases and the number of shape features (3D surface point coordinates) respectively.

Details of the PLS regression method in comparison with principal component regression are given in the Appendix. PLS regression calculates the regression coefficients $\boldsymbol{\beta}'$ as a linear

combination of M latent factors, where M<P. The latent factors are chosen to maximize the covariance between response and predictor variables.

In this paper, we use centered $\boldsymbol{Y}$ and $\boldsymbol{X}$ so that the intercept is zero. We define the normalized vector of regression coefficients (ignoring the intercept term) as the "remodelling component" associated with the corresponding remodelling index $\boldsymbol{Y}$. By analogy with PCA shape components, the remodelling component is a unit length vector in shape space (column space of $\boldsymbol{X}$). We define "remodelling scores" by analogy with PCA scores, as the projection of each case onto the remodelling component:

$$\boldsymbol{Y}_{score} = \boldsymbol{X\beta} \tag{2}$$

where $\boldsymbol{\beta}$ is the normalized regression coefficients. The estimated remodelling indices can be calculated from $\boldsymbol{Y}_{score}$ by scaling by the norm of $\boldsymbol{\beta}'$ and adding the mean index.

Orthogonal Remodelling Components

Orthogonal remodelling components are calculated following the flow chart in Figure 1. First, the remodelling index with the highest variance is chosen (EDVI). The corresponding remodelling component is calculated by PLS regression. Then a residual data matrix is generated by subtracting the projections of all cases onto the remodelling component:

$$\boldsymbol{X}^{(\mathrm{i}+1)} = \boldsymbol{X}^{\mathrm{i}} - \boldsymbol{X}^{\mathrm{i}}\boldsymbol{\beta}^{\mathrm{i}}\left(\boldsymbol{\beta}^{\mathrm{i}}\right)^{\mathrm{T}} \tag{3}$$

for i=1,…,K, where K is the number of indices. The residual data matrix is then used in the next iteration to calculate the next remodelling component, associated with the remodelling index with the next highest variance in the data set (in this case the second index is sphericity). This process is repeated for all K=6 remodelling indices (Figure 1). The resulting orthonormal remodelling components $[\boldsymbol{\beta}^1, \boldsymbol{\beta}^2, \dots, \boldsymbol{\beta}^K]$, form an orthogonal basis for a linear

sub-space of $\boldsymbol{X}$. Each $\boldsymbol{\beta}^{(i+1)}$ is orthogonal to the preceding $\boldsymbol{\beta}^{i}$ because the residual data matrix $\boldsymbol{X}^{(i+1)}$ is orthogonal to $\boldsymbol{\beta}^{i}$.

With this approach, the order of the response variables is important. We ordered the remodelling indices based on their variance in remodelling scores over the population. This is a measure of the shape variance explained by each index. The order of remodelling indices was: 1) EDVI, 2) sphericity, 3) EF, 4) relative wall thickness, 5) conicity and 6) longitudinal shortening.

Number of latent factors

Selection of the number of latent factors M has a fundamental effect on the resulting remodelling components. In the current context, there is no standard method to choose the number of latent factors. In the context of prediction, cross-validation is commonly used to examine estimation error in the response variable [23]. We compared remodelling components and scores calculated from one-factor PLS (M=1) to multi-factor PLS up to M=30 (see Figure 2). Standard 10-fold cross-validation was performed to test estimation error, showing that the mean squared error in estimating $\boldsymbol{Y}$ did not substantially improve after 10 latent factors. In terms of remodelling components, results for M>10 were similar to M=10. Experiments for 1<M<10 gave intermediate results. Therefore, in the following, we only compared two regression models: one-factor PLS (M=1) and multi-factor PLS (M=10).

Characterization of myocardial infarction

We demonstrate the clinical applicability of our proposed shape decomposition method by examining how these clinically motivated remodelling components were associated with myocardial infarction, compared to the clinical indices themselves, or PCA shape components. Logistic regression models were used to evaluate the discriminatory power of

the orthogonal remodelling components to characterize LV remodelling due to myocardial infarction. Logistic regression is a common clinical tool for examining relative effects on disease, and relative strengths of associations with disease can be quantified using odds ratios. Confounding factors (age, sex, body mass index, diastolic blood pressure, smoking status and diabetes history) were included in each regression model as baseline variables (covariates), since there were significantly different between groups in Table 1. This was done to control for the effects of these confounding factors in each of the logistic regression models. Four logistic regression models were examined. Model 1 consisted of the baseline variables and the first 6 PCA component scores. This was used as a reference for comparison. Model 2 consisted of the baseline variables and the six clinical remodelling indices. Model 3 included the baseline variables and the orthogonal remodelling component scores derived from one-factor PLS. Model 4 included the baseline variables and the orthogonal remodelling component scores derived from multi-factor PLS. In each case the presence or absence of symptomatic disease was defined by the dependant variable as 1 or 0 respectively.

Implementation

Codes were implemented in Matlab (Mathwork, Natick, MA) and R (The R Foundation, Vienna, Austria) programming languages, and are available from the Cardiac Atlas Project website[1]. The Matlab implementation requires the plsregress function from the Statistics and Machine Learning Toolbox. The R implementation requires the pls package [25]. We used SIMPLS algorithm [22] to compute the PLS regression in both versions due to its fast calculation. We compared the PLS regression coefficients using different methods provided by the pls package from R, i.e. kernel, wide kernel and classical orthogonal scores algorithms, and the results were very similar in the regression coefficients obtained.

---

[1] http://www.cardiacatlas.org/tools/lv-shape-orthogonal-clinical-modes/

Statistical analyses

Root mean square (RMS) errors in the angle between remodelling component unit vectors were used to quantify the differences arising from different training data sets: 1) asymptomatic cases from 100 to 1900, vs all asymptomatic cases, and 2) balanced data set (300 asymptomatic and 300 myocardial infarction) vs the full data set (1991 asymptomatic and 300 myocardial infarction).

For the logistic regression, the independent variables (components and baseline variables) were included simultaneously and the models were computed using SAS. A p value of <0.05 was considered significant. Four commonly-used measures were used to quantify the goodness-of-fit of the regression models: Deviance, Akaike information criterion (AIC), Bayesian information criterion (BIC) and the area under the receiver operating characteristic curve (AUC) [12]. Smaller Deviance, AIC and BIC, and larger AUC, are indicative of better goodness-of-fit. Statistical tests to determine whether the AUC of a model is significantly greater or less than another model were performed using one-sided paired non-parametric tests for AUC values [26], implemented in the pROC package [27]. A p value of <0.05 was considered as statistically higher or smaller AUC value.

Results

Unless otherwise stated all experiments were performed including all cases (asymptomatic and MI patients). Participant characteristics are summarised in Table 1. Some demographic characteristics were significantly different between the asymptomatic subjects and the myocardial infarction cases, including gender ratio, age, height, weight, blood pressure, and diabetes history. Clinical LV remodelling indices were also significantly different, as expected. The myocardial infarction patients had larger LV EDVI, increased sphericity,

thicker walls, less conicity, smaller EF and reduced longitudinal shortening than the asymptomatic subjects.

The orthogonal PLS components corresponding to EDVI, sphericity, EF, relative wall thickness, conicity and longitudinal shortening, are visualized in Figure 3 (M=1) and Figure 4 (M=10). These visualizations are useful in understanding the effect of each component on shape.

Linear correlation coefficients (Pearson) were calculated between the clinical indices and the component scores in the combined population. Correlation coefficients between PLS remodelling scores and clinical indices are reported in Table 2 for M=1 and in Table 3 for M=10. A single latent factor resulted in zero correlation between the remodelling scores and the indices corresponding to all the components previously removed in the Gram-Schmidt procedure (Table 2). Using more latent factors resulted in better correlation between each remodelling score and its corresponding index (diagonal elements are higher in Table 3 than in Table 2). Correlation coefficients between clinical indices and scores of the first six PCA components of the original dataset are shown in Table 4 for comparison.

The correlation coefficients among the clinical indices are shown in Table 5. These show strong correlations between several clinical indices. The decreasing diagonal correlations in Tables 2 and 3 are likely due to this interdependence between clinical indices. Thus, RWT and LS are related to indices previously removed by the orthogonalization process (RWT is related to EDVI and sphericity, LS is related to EF, etc).

Correlations between the PLS remodelling scores are shown in Table 6 for M=1 and in Table 7 for M=10. The minimum correlation between remodelling scores was achieved with M=1 (Table 6).

A series of experiments was performed to compare remodelling components between the full data set (1991 asymptomatic + 300 myocardial infarction) with symmetric datasets, i.e. 300 asymptomatic and 300 MI patients) with 50 trials of randomly selected asymptomatic subsets. In this case, similar remodelling components are reflected by the same unit $\boldsymbol{\beta}$ vectors, which can be measured by angle differences (derived from the dot product) between two $\boldsymbol{\beta}$ vectors. Figure 5a shows the root mean square errors (RMSE) of $\boldsymbol{\beta}$ vector differences between the subset and the full models. Only the first component (EDVI) showed less than 5 degrees difference, but increasing differences in other components were observed. This was expected since the characteristics of the cases included in the training set have an influence on the results.

Considering only the asymptomatic cases, we investigated the differences in the remodelling components with different number of samples. Figure 5b shows the RMS errors of randomly sampled cases (50 trials each) with respect to the full 1991 cases. At least 1100 cases were needed to get below 10 degrees difference with the full cohort in all components.

The results of logistic regression models to characterize remodelling associated with myocardial infarction using the orthogonal remodelling scores are shown in Table 8. For the one-factor PLS remodelling scores, the odds ratio of EDVI, sphericity, EF, wall thickness, and conicity, indicate that myocardial infarction patients tend to have larger and more spherical LV shapes with thinner walls, and a less conical shape. The multi-factor PLS remodelling scores showed somewhat different results, with EDVI, EF, Conicity and longitudinal shortening scores being significant. This may be due to the increased multi-colinearity between remodelling scores in the multi-factor case.

Table 9 shows the comparisons of the regression models. All four regression models showed significant improvement compared with the baseline variables alone. The logistic regression

based on one-factor PLS orthogonal remodelling scores showed the best Deviance, AIC and BIC and AUC. The AUC (Figure 6) for the one-factor remodelling scores was significantly greater than the multi-factor remodelling scores, and the original clinical indices, but was not significantly different from the PCA model.

The standardized coefficients of the logistic regression model were used to create a linear combination of the PLS (M=1) components generating a combined remodelling score, called the logistic regression score, separating the two groups. The F logistic regression scores (Model 3) for all cases were calculated and the median shapes were calculated by projecting the coefficients of the PLS components estimated in the logistic regression model back on the population shape space. These are plotted in Figure 7. This graphically shows the shape changes which best distinguish the two groups with baseline variables adjusted, showing that LV remodelling due to myocardial infarction is associated with larger volume, more spherical shape, and thinner wall thickness. Since the logistic regression coefficients refer to contributions from remodelling components, the amount of each remodelling component contributing to the logistic regression score could be quantified. This gives an intuitive explanation of the logistic regression score in terms of remodelling components associated with clinical remodelling indices.

Discussion

Patients with myocardial infarction exhibit significant shape changes with respect to the normal population, due to cardiac remodelling. An atlas-based analysis of cardiac remodelling has previously shown better characterization of remodelling due to myocardial infarction than traditional mass and volume analysis in large data sets [12]. The framework consisted of three steps: (1) fitting a finite element model to the LV MR images, (2) shape component extraction from the aligned shapes, and (3) quantification of the association

between the components and disease using logistic regression. Although PCA provides orthogonal shape components, which describe the maximum amount of variation for the fewest number of components, these components typically do not correspond with clinical indices of cardiac remodelling. To avoid this problem, and give the components a clear clinical interpretation, while maintaining the advantages of orthogonality, we developed a method to generate orthogonal shape components from any set of clinical indices using PLS.

In this paper, we generated a linear orthogonal shape basis from the full finite element shape parameters. Clinical indices, such as EDVI, sphericity, EF, relative wall thickness, conicity and longitudinal shortening, were derived from the finite element shape model. Similar to PCA, the shape components derived from PLS regression are orthogonal. In PCA, the resulting component scores also have zero correlation across the population cohort, but this is not the case with PLS. Table 7 shows that PLS component scores with M=10 were significantly correlated, similar to the original clinical indices in Table 5. This is expected since M=10 results in strong correlations between scores and indices (Table 3). PLS components both using M=10 and M=1 obtain effective shape representation for each clinical index, as evidenced by the correlation coefficients with the clinical indices (diagonal terms in Tables 2 and 3), compared to the first six components of PCA (Table 4).

We found that the correlations between the scores of different indices for PLS with M=1 become smaller than the original indices and scores of PLS with M=10. For example, the correlation between EDVI and EF was originally -0.60 (Table 5), then became -0.68 from PLS with M=10 (Table 7); however it was -0.15 from PLS with M=1 (Table 6). Not only did a single latent factor result in the least correlation between component scores (Table 6), but it also resulted in zero correlation between component scores and previously removed indices (upper triangle of Table 2). This result is a feature of one-factor PLS applied in this context. One-factor PLS computes a single latent factor which maximizes the cross-correlation

between $\boldsymbol{X}$ and $\boldsymbol{Y}$. The resulting remodelling component is a vector in the same direction as this single latent factor (in fact $\boldsymbol{\beta} \propto \boldsymbol{X}^T\boldsymbol{Y}$). Subtracting this component from the shape space leads to zero correlation between the residual shapes and $\boldsymbol{Y}$. For multi-factor PLS, the resulting remodelling component is a combination of all the latent factors, and no longer has this property.

These orthogonal components derived from traditional remodelling indices may be used to partition shape into contributions from each component, independent of the others. Correlation analysis shows that these clinically derived components have high correspondence with traditional remodelling indices (diagonals in Tables 2 and 3), either virtually following the clinical indices' original correlation (Table 5) in M=10 (Table 3), or by sacrificing some of the diagonal correlations in exchange for decoupling with previous indices in M=1 (Table 2). Remodelling scores at M=10 are more correlated with the original clinical indices than M=1 but at the expense of their ability to explain variance in the original shape space. It can therefore be argued that M=10 generates more 'specific' shapes with lesser representative power.

Previous studies have also used PLS to derive information on cardiac remodelling [28]. Lekadir et al. [28] used PLS to characterize myocardial infarction using class labels as the response variable and the data matrix as the predictor variables. They found that running the regression with a range of latent factors and combining the estimations with a median operator could obtain better performance. In the current paper, logistic regression was used (instead of PLS in [28]) with the class labels as the response variable, because this is a commonly used clinical tool to examine associations with disease, and it is simple to calculate relative effects of the components on the response variable as odds ratios. The current paper also differs from [28] in the use of PLS to derive orthogonal remodelling

components and the finding that a single latent factor reduces correlations in the resulting remodelling scores.

The results also show that clinically derived components quantitatively characterise remodelling associated with myocardial infarction with similar power as PCA components. Three logistic regression models based on the clinical indices, PCA components and orthogonal remodelling components derived from clinical indices were all similar in terms of goodness of fit. Significance tests on areas under the ROC curves (AUC) revealed that the one-factor PLS model showed significantly greater AUC compared with the multi-factor PLS model and the clinical indices model, but not significantly different from the PCA model. Hence the single latent factor remodelling components characterised myocardial infarction similarly to PCA, while having the added advantage of having clear clinical interpretation with respect to their corresponding clinical indices, as well as being an orthogonal decomposition of shape space.

Coefficients of the remodelling components estimated in the logistic regression model were projected back on the population shape space. Figure 7 visualises the shape changes characterizing presence of disease. This combined component can be used for tracking individual patients over time in future studies, by quantifying the degree to which their LV shapes compare with the remodelling spectrum.

In this study, we included all of the available cases (1,991 asymptomatic and 300 myocardial infarction), since we were primarily interested in the proof of concept. Having a balanced data set is preferable to enable the analysis of differences between “asymptomatic remodelling” and “symptomatic remodelling”, which would be of considerable interest in terms of physiological driving factors. However, Figure 5b indicates that over 1000 cases would be required for robust identification of remodelling components. Also, physiological

functions between different pathological groups can be quite different. For example, comparing the remodelling components of 1991 asymptomatic subjects only with remodelling components of 1991 asymptomatic + 300 myocardial infarction revealed differences of 9.1 degrees in EDVI, 6.4 degrees in sphericity, 15.1 degrees in EF, 7.0 degrees in RWT, 9.5 degrees in conicity and 8.4 degrees in longitudinal shortening. Hence, the myocardial infarction patients, which were only 24% from all samples, had a significant influence on all the remodelling components.

Supervised dimension reduction techniques such as information maximising component analysis and linear discriminate analysis have also been used to extract a single remodelling component which can best characterize myocardial infarction using surface sampling [29]. In the current study, the shape components of each clinical index were obtained first and then combined using logistic regression. The shape changes due to myocardial infarction obtained by this logistic regression model can therefore be more easily explained as a combination of well-understood shape components, through the logistic regression coefficients.

This method can be applied to any set of (moderately independent) clinical measures, enabling visualization and quantification of the corresponding shape components, thereby further exploiting shape information in a clinically meaningful fashion.

Limitations

The cross-sectional nature of these data limits the understanding that can be gained on the physiological factors underlying remodelling processes. However, the methods developed in this work can be applied to future studies to track patients over time, or to epidemiological studies such as the Multi-Ethnic Study of Atherosclerosis [30] and the UK Biobank [31]. We also limited the clinical remodelling indices examined in this paper to those geometric indices which have been well established in the clinical literature. These indices are also readily

available from several imaging modalities such as 3D echo and CT. The order the indices are included in the basis has an effect on the resulting remodelling components. While we used the variance of the corresponding remodelling scores (a measure of shape variance explained), other methods are possible and this requires further research. Finally, we did not include structural information on the location and size of the infarct. While more information is becoming available on the interesting effects of infarct size and transmurality, this is left for future work. Also, many patients have comorbidities such as valvular disease, which was not examined in the current study.

Potential implications

An orthogonal decomposition of shape in relation to remodelling indices of known prognostic value will enable multi-dimensional characterization of the ways in which the heart adapts with the progression of disease, e.g. after myocardial infarction. The remodelling components were able to characterize disease as well as standard methods, with the added advantages of having clear clinical interpretation with respect to their corresponding clinical indices, as well as being an orthogonal decomposition of shape space. The resulting remodelling scores can be used to track the progression of remodelling over time, against reference populations. This would enable automatic computation of z-scores giving precise information on how the patient's heart compares against the reference population. Although the remodelling components were generated from a largely asymptomatic population in this work, we showed how they describe the shape changes undergone in myocardial infarction relatively well. We also showed how the amount of each remodelling component could be quantified in association with the presence of clinical disease, highlighting significant contributions of ventricular size, sphericity and relative wall thickness. These methods enable new knowledge to be derived from medical imaging examinations on the underlying mechanisms driving the

adaptation of the heart in response to disease. Future work can also examine how the remodelling scores are related to future adverse events, e.g. using clinical outcomes.

Availability of supporting data and materials

All data and results are available from www.cardiacatlas.org. The data are not publically available due to IRB restrictions on the contributing studies; however, data are made available on approval of a research application submitted under the Cardiac Atlas Project data sharing policy (www.cardiacatlas.org). Data further supporting this work are available in the *GigaScience* repository, GigaDB [32].

Declarations

Abbreviations and Acronyms

Left ventricular =LV，Ejection Fraction = EF, Principal Component Analysis = PCA, Partial Least Squares = PLS, End-diastolic Volume Index (EDVI), Myocardial infarction =MI，Ejection fraction =EF，Longitudinal shortening =LS， Relative wall thickness = RWT, Systolic Blood Pressure=SBP, Diastolic blood pressure=DBP

Ethics approval and consent to participate

This study was approved by the local institutional review boards (Johns Hopkins University School of Medicine NA_00031350; Northwestern University CR1_STU00000078; New Zealand Multi-region Ethics Committee MEC/08/04/052) and all participants gave written informed consent.

Consent for Publication

Not applicable

Competing interests

The authors declare that they have no competing interests.

Funding

This project was supported by award numbers R01HL087773 and R01HL121754 from the National Heart, Lung, and Blood Institute. David A. Bluemke is supported by the NIH intramural research program. Xingyu Zhang was supported by the China Scholarship Council.

Authors' contributions

All authors were involved in the design of the study, interpretation of the data, drafting and revision of the manuscript, and final approval of the submitted manuscript. XZ, PM-G, and AS performed the statistical analyses.

Authors' Information

XZ is a biostatistician. PM-G is a biostatistician and expert in bioinformatics. BA-V is a bioengineer and expert in medical image analysis. DB is a radiologist and Director of Radiology and Imaging Sciences at the National Institute of Biomedical Imaging and Bioengineering. BR is a clinical engineer and an expert in cardiac MRI. JPF is a radiologist and Director of Magnetic Resonance Research at UCLA. AK, DL and JL are cardiologists. AY is a bioengineer and PI of the Cardiac Atlas Project and head of Department of Anatomy and Medical Imaging at the University of Auckland. AS is an expert in atlas-based medical image analysis.

Appendix

Principal Component Regression

Let $\boldsymbol{X} \in \mathbb{R}^{N\times P}$be a data matrix of predictor variables where each row is a case (shape vector) and each column a shape feature (in our case [x y z] coordinates of sampled points). There are N cases and P shape features. We first "column center" the data by subtracting the mean across cases.

Principal Component Analysis (PCA) decomposes $\boldsymbol{X}$ into an othonormal matrix $\boldsymbol{\Phi} \in \mathbb{R}^{P\times M}$ containing eigenvectors of the covariance matrix $\boldsymbol{X}^{\mathrm{T}}\boldsymbol{X}$. The columns of $\boldsymbol{\Phi}$ define "shape components". M is the number of shape components used to approximate $\boldsymbol{X}$, typically $M<P$, by

$$\boldsymbol{X}_{est} = \boldsymbol{T}\boldsymbol{\Phi}^{T} \tag{A.1}$$

$$\boldsymbol{T} = \boldsymbol{X}\boldsymbol{\Phi} \tag{A.2}$$

where $\boldsymbol{T} \in \mathbb{R}^{N\times M}$ is a matrix of "scores". Each case is thus approximated by a linear combination of shape components. The weights of the combination (rows of $\boldsymbol{T}$) are the amount of each shape component present in that case, and are calculated by projecting each shape vector onto the shape component.

In principal component regression (PCR), the response or dependent variable $\boldsymbol{Y}$ (at present we consider a single response variable being a centered remodeling index such as EDVI) is regressed against the principal component scores (scores being used as predictor variables):

$$\boldsymbol{Y}_{est} = \boldsymbol{T}\boldsymbol{B}_{PCR} \tag{A.3}$$

where $\boldsymbol{B}_{PCR}$ is a vector of regression coefficients.

The advantage of this method is that the regression coefficients do not suffer from the well-known multicolinearity problem, in which the regression coefficients can be ill-defined if the independent variables are correlated, leading to instability in future predictions. Note that in PCA the resulting scores $\boldsymbol{T}$ are orthogonal, so the resulting scores have zero correlation within the dataset between different component scores.

PCR Remodeling Component:

The PCR can be written as

$$\boldsymbol{Y}_{est} = \boldsymbol{T}\boldsymbol{B}_{PCR} = \boldsymbol{X}\boldsymbol{\Phi}\boldsymbol{B}_{PCR} = \boldsymbol{X}\boldsymbol{\beta}'_{PCR} \tag{A.4}$$

Here $\boldsymbol{X}$ are the predictor variables and the regression coefficients are calculated from the PCR as $\boldsymbol{\beta}'_{PCR} = \boldsymbol{\Phi}\boldsymbol{B}_{PCR}$. This vector of regression coefficients can be thought of as the linear combination of shape components that best predict the response variable. We define a "PCR remodeling component" $\boldsymbol{\beta}_{PCR}$by normalizing $\boldsymbol{\beta}'_{PCR}$ (note the data and response are centered so we exclude the zero intercept). The PCR remodelling scores are defined as follows:

$$\boldsymbol{Y}_{PCRscore} = \frac{\boldsymbol{X}\boldsymbol{\beta}'_{PCR}}{|\boldsymbol{\beta}'_{PCR}|} = \boldsymbol{X}\boldsymbol{\beta}_{PCR} \tag{A.5}$$

The remodelling score for each case is then a projection (inner product) of the shape vector on the remodelling component. The remodelling component is defined by analogy to PCA shape components as a unit length direction in shape space. Remodelling scores are defined by analogy to shape scores in PCA; we can get the estimated remodelling index from $\boldsymbol{Y}_{PCRscore}$ by scaling by the norm of $\boldsymbol{\beta}'_{PCR}$ and adding the mean.

Partial Least Squares Regression

A problem with PCR is that the independent variables are chosen by their ability to explain variance in $\boldsymbol{X}$, not $\boldsymbol{Y}$. Partial least squares (PLS) regression solves this problem by finding the

“latent factors” that best explain the covariance between $\boldsymbol{Y}$ and $\boldsymbol{X}$. These are ranked from largest to smallest covariance, so the first factor explains the most covariance, the second factor for the second largest covariance, and so on.

PLS finds a linear decomposition of $\boldsymbol{X}$ and $\boldsymbol{Y}$ such that

$$\boldsymbol{X} = \boldsymbol{T}\boldsymbol{\Psi}^T + \boldsymbol{E}_X \tag{A.6}$$

$$\boldsymbol{Y} = \boldsymbol{U}\boldsymbol{\Omega}^T + \boldsymbol{E}_Y \tag{A.7}$$

where $\boldsymbol{T} \in \mathbb{R}^{N\times M}$ and $\boldsymbol{U} \in \mathbb{R}^{N\times M}$ are PLS scores for predictor and response variables, respectively. Similarly, $\boldsymbol{\Psi} \in \mathbb{R}^{P\times M}$ and $\boldsymbol{\Omega} \in \mathbb{R}^{K\times M}$ (K=1 for a single response variable) are the PLS loadings for the predictor and response variables. Unlike PCR, $\boldsymbol{\Psi}$ and $\boldsymbol{\Omega}$ are not orthogonal and not normalized. The parameter $M \leq P$ is the number of latent factors, typically determined by examining the percentage variance explained in $\boldsymbol{Y}$.

PLS derives the $\boldsymbol{\beta}$ regression coefficients as linear combinations of the latent factors, which are chosen to maximize correlation between response and predictor variables. Several variants exist in the literature, differing in the calculation of $\boldsymbol{T}$ [21, 22]. However, similar to PCR, we can define PLS remodelling components and remodelling scores as

$$\boldsymbol{Y}_{PLSscore} = \frac{\boldsymbol{X}\boldsymbol{\beta}'_{PLS}}{|\boldsymbol{\beta}'_{PLS}|} = \boldsymbol{X}\boldsymbol{\beta}_{PLS} \tag{A.8}$$

As for PCR, the estimated $\boldsymbol{Y}$ can be derived from the scores by scaling by $|\boldsymbol{\beta}'_{PLS}|$ and adding the mean.

Orthogonal Remodelling Components

The orthogonalization process given in (3) can be applied to the results of PCR or PLS regression. PLS regression is always more efficient than PCA regression, in that fewer terms

are required to capture the variance of the response variable. However, if all PCA components are included in the PCR, and all latent factors in the PLS, the two methods are equivalent. One-factor PLS (ie M=1 in the PLS regression) has particular properties which may make it attractive in some applications. For example one-factor PLS has been shown to be equivalent to rescaled ridge regression as the ridge parameter tends to infinity [22].

For K>1, ie more than one response variable included in Y, the PLS regression finds latent factors which explain the most covariance between the ***X*** and ***Y*** matrices simultaneously. This was not considered for the current work because the resulting regression coefficients are not orthogonal.

References

1. Sutton MGSJ, Sharpe N: **Left Ventricular Remodeling After Myocardial Infarction: Pathophysiology and Therapy.** *Circulation* 2000, **101:**2981-2988.
2. Gjesdal O, Bluemke DA, Lima JA: **Cardiac remodeling at the population level[mdash]risk factors, screening, and outcomes.** *Nat Rev Cardiol* 2011, **8:**673-685.
3. Lieb W, Gona P, Larson MG, Aragam J, Zile MR, Cheng S, Benjamin EJ, Vasan RS: **The Natural History of Left Ventricular Geometry in the CommunityClinical Correlates and Prognostic Significance of Change in LV Geometric Pattern.** *JACC: Cardiovascular Imaging* 2014, **7:**870-878.
4. Zile MR, Gaasch WH, Patel K, Aban IB, Ahmed A: **Adverse left ventricular remodeling in community-dwelling older adults predicts incident heart failure and mortality.** *JACC: Heart Failure* 2014.
5. Wong SP, French JK, Lydon A-M, Manda SOM, Gao W, Ashton NG, White HD: **Relation of left ventricular sphericity to 10-year survival after acute myocardial infarction.** *The American journal of cardiology* 2004, **94:**1270-1275.
6. White HD, Norris RM, Brown MA, Brandt PW, Whitlock RM, Wild CJ: **Left ventricular end-systolic volume as the major determinant of survival after recovery from myocardial infarction.** *Circulation* 1987, **76:**44-51.
7. Di Donato M, Dabic P, Castelvecchio S, Santambrogio C, Brankovic J, Collarini L, Joussef T, Frigiola A, Buckberg G, Menicanti L, the RG: **Left ventricular geometry in normal and post-anterior myocardial infarction patients: sphericity index and 'new' conicity index comparisons.** *European Journal of Cardio-Thoracic Surgery* 2006, **29:**S225-S230.
8. Konstam MA, Kramer DG, Patel AR, Maron MS, Udelson JE: **Left Ventricular Remodeling in Heart FailureCurrent Concepts in Clinical Significance and Assessment.** *JACC: Cardiovascular Imaging* 2011, **4:**98-108.
9. Anand I, McMurray J, Cohn JN, Konstam MA, Notter T, Quitzau K, Ruschitzka F, Lüscher TF: **Long-term effects of darusentan on left-ventricular remodelling and clinical outcomes in the Endothelin A Receptor Antagonist Trial in Heart Failure (EARTH): randomised, double-blind, placebo-controlled trial.** *The Lancet* 2004, **364:**347-354.
10. Kramer CM, Lima JA, Reichek N, Ferrari VA, Llaneras MR, Palmon LC, Yeh IT, Tallant B, Axel L: **Regional differences in function within noninfarcted myocardium during left ventricular remodeling.** *Circulation* 1993, **88:**1279-1288.
11. Jolliffe I: *Principal component analysis.* Wiley Online Library; 2005.
12. Zhang X, Cowan BR, Bluemke DA, Finn JP, Fonseca CG, Kadish AH, Lee DC, Lima JAC, Suinesiaputra A, Young AA, Medrano-Gracia P: **Atlas-Based Quantification of Cardiac Remodeling Due to Myocardial Infarction.** *PLoS ONE* 2014, **9:**e110243.
13. Medrano-Gracia P, Cowan BR, Ambale-Venkatesh B, Bluemke DA, Eng J, Finn JP, Fonseca CG, Lima JAC, Suinesiaputra A, Young AA: **Left ventricular shape variation in asymptomatic populations: the multi-ethnic study of atherosclerosis.** *Journal of Cardiovascular Magnetic Resonance* 2014, **16:**56.
14. Remme EW, Young AA, Augenstein KF, Cowan B, Hunter PJ: **Extraction and quantification of left ventricular deformation modes.** *IEEE Trans Biomed Eng* 2004, **51:**1923-1931.
15. Fonseca CG, Backhaus M, Bluemke DA, Britten RD, Do Chung J, Cowan BR, Dinov ID, Finn JP, Hunter PJ, Kadish AH: **The Cardiac Atlas Project—an imaging database for computational modeling and statistical atlases of the heart.** *Bioinformatics* 2011, **27:**2288-2295.
16. Medrano-Gracia P, Cowan BR, Bluemke DA, Finn JP, Kadish AH, Lee DC, Lima JAC, Suinesiaputra A, Young AA: **Atlas-based analysis of cardiac shape and function: correction of regional shape bias due to imaging protocol for population studies.** *Journal of Cardiovascular Magnetic Resonance* 2013, **15:**80.

17. Allgower EL, Schmidt PH: **Computing Volumes of Polyhedra.** *Mathematics of Computation* 1986, **46:**171-174.
18. Schulz-Menger J, Bluemke DA, Bremerich J, Flamm SD, Fogel MA, Friedrich MG, Kim RJ, von Knobelsdorff-Brenkenhoff F, Kramer CM, Pennell DJ, et al: **Standardized image interpretation and post processing in cardiovascular magnetic resonance: Society for Cardiovascular Magnetic Resonance (SCMR) board of trustees task force on standardized post processing.** *J Cardiovasc Magn Reson* 2013, **15:**35.
19. Li L, Shigematsu Y, Hamada M, Hiwada K: **Relative Wall Thickness Is an Independent Predictor of Left Ventricular Systolic and Diastolic Dysfunctions in Essential Hypertension.** *Hypertension Research* 2001, **24:**493-499.
20. Izumo M, Lancellotti P, Suzuki K, Kou S, Shimozato T, Hayashi A, Akashi YJ, Osada N, Omiya K, Nobuoka S, et al: *Three-dimensional echocardiographic assessments of exercise-induced changes in left ventricular shape and dyssynchrony in patients with dynamic functional mitral regurgitation.* 2009.
21. Geladi P, Kowalski BR: **Partial least-squares regression: a tutorial.** *Analytica chimica acta* 1986, **185:**1-17.
22. de Jong S, Phatak A: **Partial least squares regression.** *Recent advances in total least squares techniques and errors-in-variables modeling* 1997:311-338.
23. Gómez-Carracedo MP, Andrade JM, Rutledge DN, Faber NM: **Selecting the optimum number of partial least squares components for the calibration of attenuated total reflectance-mid-infrared spectra of undesigned kerosene samples.** *Analytica Chimica Acta* 2007, **585:**253-265.
24. Zhang X, Ambale-Venkatesh B, Bluemke D, Cowan B, Finn JP, Hundley W, Kadish A, Lee D, Lima JC, Suinesiaputra A, et al: **Orthogonal Shape Modes Describing Clinical Indices of Remodeling.** In *Functional Imaging and Modeling of the Heart. Volume* 9126. Edited by van Assen H, Bovendeerd P, Delhaas T: Springer International Publishing; 2015: 273-281: *Lecture Notes in Computer Science*].
25. Mevik B-H, Wehrens R: **The pls Package: Principal Component and Partial Least Squares Regression in R.** *J Stat Softw* 2007, **18:**1--23.
26. DeLong ER, DeLong DM, Clarke-Pearson DL: **Comparing the areas under two or more correlated receiver operating characteristic curves: a nonparametric approach.** *Biometrics* 1988, **44:**837-845.
27. Robin X, Turck N, Hainard A, Tiberti N, Lisacek F, Sanchez JC, Muller M: **pROC: an open-source package for R and S+ to analyze and compare ROC curves.** *BMC Bioinformatics* 2011, **12:**77.
28. Lekadir K, Albà X, Pereañez M, Frangi AF: **Statistical Shape Modeling Using Partial Least Squares: Application to the Assessment of Myocardial Infarction.** In *Statistical Atlases and Computational Models of the Heart Imaging and Modelling Challenges: 6th International Workshop, STACOM 2015, Held in Conjunction with MICCAI 2015, Munich, Germany, October 9, 2015, Revised Selected Papers.* Edited by Camara O, Mansi T, Pop M, Rhode K, Sermesant M, Young A. Cham: Springer International Publishing; 2016: 130-139
29. Zhang X, Ambale-Venkatesh B, Bluemke D, Cowan B, Finn J, Kadish A, Lee D, Lima J, Hundley W, Suinesiaputra A, et al: **Information maximizing component analysis of left ventricular remodeling due to myocardial infarction.** *Journal of Translational Medicine* 2015, **13:**343.
30. Bild DE, Bluemke DA, Burke GL, Detrano R, Diez Roux AV, Folsom AR, Greenland P, Jacob DR, Jr., Kronmal R, Liu K, et al: **Multi-Ethnic Study of Atherosclerosis: objectives and design.** *Am J Epidemiol* 2002, **156:**871-881.
31. Petersen SE, Matthews PM, Bamberg F, Bluemke DA, Francis JM, Friedrich MG, Leeson P, Nagel E, Plein S, Rademakers FE, et al: **Imaging in population science: cardiovascular magnetic resonance in 100,000 participants of UK Biobank - rationale, challenges and approaches.** *J Cardiovasc Magn Reson* 2013, **15:**46.

32. Zhang X, Medrano-Gracia P, Ambale-Venkatesh B, Bluemke DA, Cowan BR, Finn JP, Kadish AH, Lee DC, Lima JA, Young AA, Suinesiaputra A: Supporting data for "Orthogonal Decomposition of Left Ventricular Remodelling in Myocardial Infarction" GigaScience Database. 2017. http://dx.doi.org/10.5524/100273

Tables

Table 1 Demographics and clinical remodelling indices for asymptomatic subjects and patients with myocardial infarction (mean ± SD). MI=Myocardial infarction; BMI=Body mass index; SBP=Systolic blood pressure; DBP=diastolic blood pressure; EDVI= end diastolic volume index; RWT=relative wall thickness; EF= ejection fraction; LS=longitudinal shortening.

Variable	Unit	Asymptomatic	MI cases	p-value
Sex	F/M	1034/975	60/238	<0.01
Age	years	61.47±10.15	62.76±10.76	0.043
Height	cm	165.98±9.99	173.82±9.77	<0.001
Weight	kg	76.75±16.50	90.06+14.14	<0.001
BMI		27.77±5.09	29.73+5.57	<0.001
SBP	mmHg	126.28±21.98	126.36±17.50	>0.05
DBP	mmHg	71.49±10.33	73.26±9.82	0.006
Diabetes history	%	13.11	35.67	<0.001
Smoking status	%	12.51	11.33	>0.05
EDVI		67.83±13.29	96.53±25.03	<0.001
Sphericity		0.38±0.08	0.41±0.09	<0.001
RWT	%	39.71±9.49	35.21±8.38	<0.001
Conicity		0.74±0.08	0.70±0.08	<0.001
EF		0.63±0.07	0.41±0.11	<0.001
LS		0.13±0.04	0.08±0.03	<0.001

Table 2 Correlation coefficients between the clinical indices and the PLS remodelling component scores (M=1). EDVI= end diastolic volume index; RWT=relative wall thickness; EF= ejection fraction; LS=longitudinal shortening.

	EDVI score	Sphericity score	EF score	RWT score	Conicity score	LS score
EDVI	0.82	0	0	0	0	0
Sphericity	0.03	0.83	0	0	0	0
EF	-0.75	0.03	0.61	0	0	0
RWT	-0.20	-0.16	-0.04	0.53	0	0
Conicity	-0.14	-0.28	0.30	0.21	0.72	0
LS	-0.45	0.03	0.61	-0.17	0.20	0.53

Table 3 Correlation coefficients between the clinical indices and the PLS remodelling component scores (M=10). EDVI= end diastolic volume index; RWT=relative wall thickness; EF= ejection fraction; LS=longitudinal shortening.

	EDVI score	Sphericity score	EF score	RWT score	Conicity score	LS score
EDVI	0.94	0.27	-0.34	-0.64	-0.13	-0.31
Sphericity	0.30	0.97	-0.15	-0.16	-0.25	-0.13
EF	-0.41	-0.28	0.90	0.22	0.25	-0.02
RWT	-0.65	-0.12	0.26	0.99	0.25	0.53
Conicity	-0.13	-0.22	0.38	0.25	0.97	0.24
LS	-0.32	-0.13	0.02	0.56	0.25	0.98

Table 4 Correlation coefficients between the clinical indices and the first 6 PCA shape components. EDVI= end diastolic volume index; RWT=relative wall thickness; EF= ejection fraction; LS=longitudinal shortening.

	PC 1	PC 2	PC 3	PC 4	PC 5	PC 6
EDVI	0.80	-0.01	-0.74	-0.18	-0.13	-0.45
Sphericity	-0.26	-0.80	0.19	0.19	0.30	0.06
EF	-0.01	0.09	-0.11	0.03	-0.09	-0.20
RWT	0.10	0.24	-0.21	-0.25	-0.25	-0.18
Conicity	0.10	0.13	-0.15	-0.11	-0.15	-0.14
LS	0.21	0.02	0.03	-0.15	0.50	0.37

Table 5 Correlation coefficients among the clinical indices. EDVI= end diastolic volume index; RWT=relative wall thickness; EF= ejection fraction; LS=longitudinal shortening.

	EDVI	Sphericity	EF	RWT	Conicity	LS
EDVI	1	0.28	-0.60	-0.37	-0.11	-0.29
Sphericity	0.28	1	-0.11	-0.28	-0.22	-0.13
EF	-0.60	-0.11	1	0.18	0.26	0.57
RWT	-0.37	-0.28	0.18	1	0.32	0.00
Conicity	-0.11	-0.22	0.26	0.32	1	0.26
LS	-0.29	-0.13	0.57	0.00	0.26	1

Table 6 Correlation coefficients among the PLS remodelling scores (M=1). EDVI= end diastolic volume index; RWT=relative wall thickness; EF= ejection fraction; LS=longitudinal shortening.

	EDVI score	Sphericity score	EF score	RWT score	Conicity score	LS score
EDVI score	1	-0.29	-0.15	0.22	-0.15	-0.08
Sphericity score	-0.29	1	0.001	-0.04	0.01	0.22
EF score	-0.15	0.001	1	0.09	0.09	0.47
RWT score	0.22	-0.04	0.09	1	-0.08	0.002
Conicity score	-0.15	0.01	0.09	-0.08	1	0.16
LS score	-0.08	0.22	0.47	0.002	0.16	1

Table 7 Correlation coefficients among the PLS remodelling scores (M=10). EDVI= end diastolic volume index; RWT=relative wall thickness; EF= ejection fraction; LS=longitudinal shortening.

	EDVI score	Sphericity score	EF score	RWT score	Conicity score	LS score
EDVI score	1	0.29	-0.68	-0.37	-0.15	-0.34
Sphericity score	0.29	1	-0.17	-0.15	-0.25	-0.14
EF score	-0.68	-0.17	1	0.27	0.25	0.53
RWT score	-0.37	-0.15	0.27	1	0.31	-0.01
Conicity score	-0.15	-0.25	0.25	0.31	1	0.24
LS score	-0.34	-0.14	0.53	-0.01	0.24	1

Table 8 Four logistic regressions for myocardial infarction. EDVI= end diastolic volume index; RWT=relative wall thickness; EF= ejection fraction; LS=longitudinal shortening.

Variable	Coefficient	Standard error	p value	Standardized Coefficient	Odds Ratio(OR)	OR 95% Confidence Interval	
Model 1: PCA shape components + Baseline variables							
**PC 1**	**2.644**	**0.177**	**<.0001**	**1.455**	**14.066**	**9.942**	**19.901**
**PC 2**	**-0.605**	**0.102**	**<.0001**	**-0.334**	**0.546**	**0.447**	**0.666**
PC 3	0.071	0.112	0.524	0.039	1.074	0.863	1.336
**PC 4**	**2.031**	**0.153**	**<.0001**	**1.111**	**7.625**	**5.652**	**10.287**
**PC 5**	**0.391**	**0.106**	**<.0001**	**0.215**	**1.478**	**1.200**	**1.821**
PC 6	-0.113	0.119	0.342	-0.062	0.893	0.708	1.127
Model 2: Clinical indices + Baseline variables							
**EDVI**	**0.041**	**0.008**	**<.0001**	**0.412**	**1.042**	**1.027**	**1.058**
Sphericity	0.002	0.014	0.870	0.010	1.002	0.975	1.030
**EF**	**-0.164**	**0.015**	**<.0001**	**-0.966**	**0.849**	**0.825**	**0.874**
RWT	0.002	0.014	0.875	0.012	1.002	0.975	1.030
**Conicity**	**-0.037**	**0.016**	**0.018**	**-0.161**	**0.963**	**0.934**	**0.994**
**LS**	**-0.148**	**0.037**	**<.0001**	**-0.325**	**0.862**	**0.802**	**0.927**
Model 3: PLS remodelling scores (M=1) + Baseline variables							
**EDVI score**	**2.859**	**0.191**	**<.0001**	**1.574**	**17.444**	**11.997**	**25.365**
**Sphericity score**	**0.895**	**0.125**	**<.0001**	**0.492**	**2.446**	**1.915**	**3.124**
**EF score**	**-1.540**	**0.148**	**<.0001**	**-0.846**	**0.214**	**0.160**	**0.287**
**RWT score**	**-1.289**	**0.146**	**<.0001**	**-0.710**	**0.275**	**0.207**	**0.367**
**Conicity score**	**0.331**	**0.124**	**0.007**	**0.181**	**1.392**	**1.093**	**1.774**
LS score	-0.041	0.140	0.769	-0.023	0.960	0.729	1.263
Model 4: PLS remodelling scores (M=10) + Baseline variables							
**EDVI score**	**0.823**	**0.161**	**<.0001**	**0.454**	**2.277**	**1.661**	**3.120**
Sphericity score	-0.189	0.114	0.098	-0.103	0.828	0.662	1.036
**EF score**	**-1.843**	**0.180**	**<.0001**	**-1.016**	**0.158**	**0.111**	**0.225**
RWT score	0.087	0.128	0.495	0.048	1.091	0.849	1.403
**Conicity score**	**-0.393**	**0.122**	**0.001**	**-0.216**	**0.675**	**0.531**	**0.858**
**LS score**	**-0.665**	**0.141**	**<.0001**	**-0.365**	**0.514**	**0.390**	**0.678**

All the models are adjusted for age, gender, BMI, DBP, smoking status and diabetes history. Bold rows indicate p<0.05.

Table 9 Comparison of the four logistic regression models. AIC = Akaike information criterion ; BIC =Bayesian information criterion; AUC =Area under the ROC curve. Smaller Deviance, AIC and BIC, and larger AUC, are indicative of better goodness-of-fit. Bold row indicates best performance.

	Deviance	AIC	BIC	AUC
Baseline	1560	1574	1615	0.7415
Indices	710	727	802	0.9594
PCA scores	607	633	708	0.9725
**PLS scores (M=1)**	**569**	**595**	**669**	**0.9739**
PLS scores (M=10)	683	709	784	0.9598

Figure 1 Click here to download Figure Figure 1 .docx

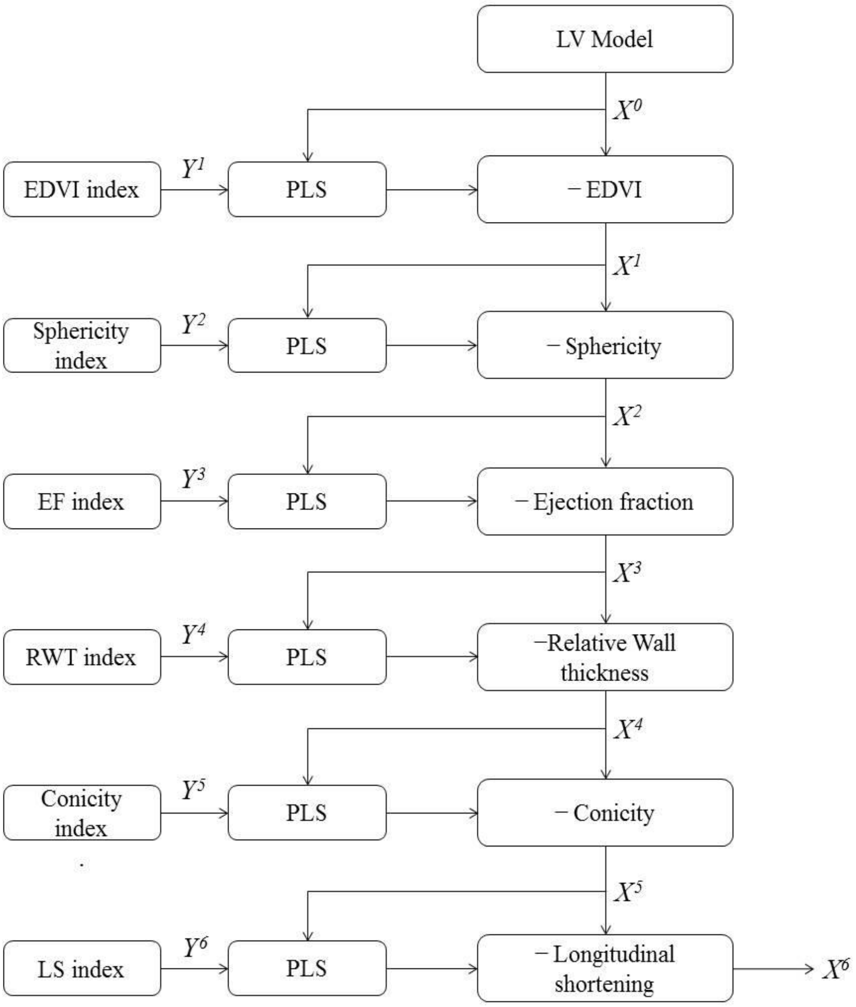

Figure 1 Data processing flow chart. LV=left ventricle; X = shape space; Y = response variable; PLS = partial least squares; EDVI= end diastolic volume index; RWT=relative wall thickness; EF= ejection fraction; LS=longitudinal shortening.

Figure 2 Click here to download Figure Figure 2.docx

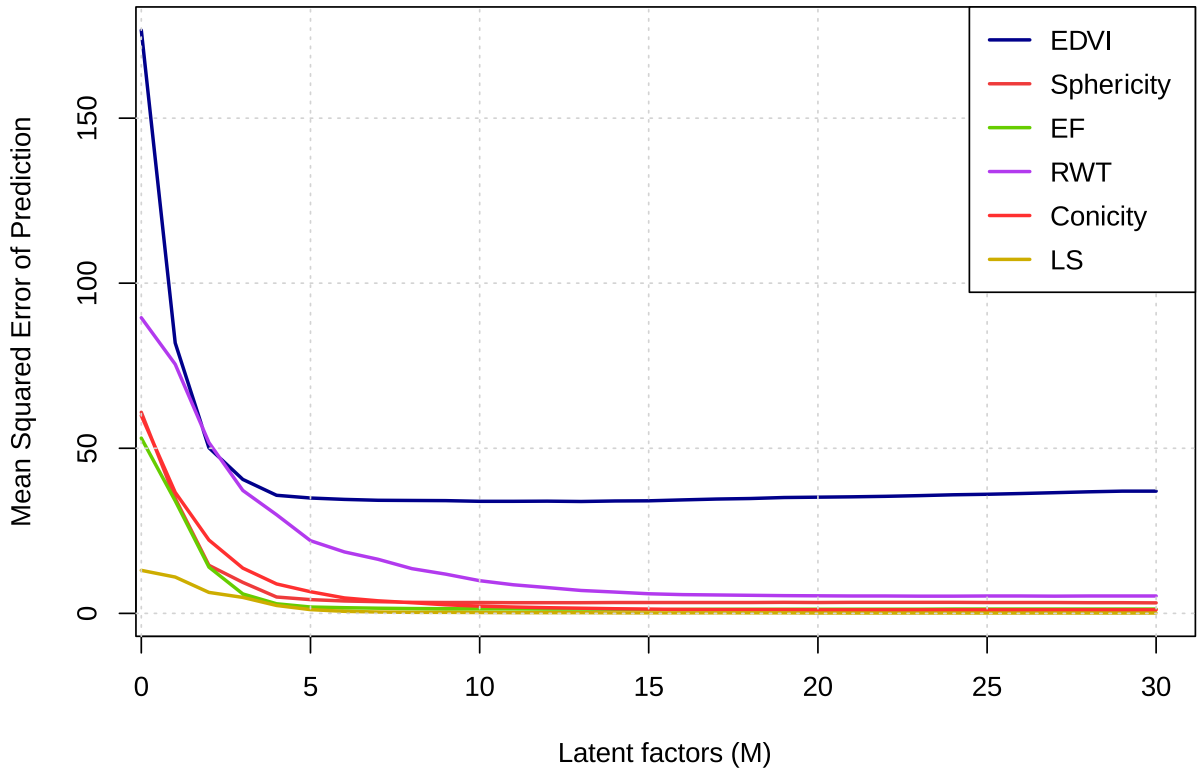

Figure 2. Mean squared error predictions of PLS regression coefficients using different number of latent factors (M). 10-fold cross validations were applied. EDVI= end diastolic volume index; RWT=relative wall thickness; EF= ejection fraction; LS=longitudinal shortening.

Figure 3 Click here to download Figure Figure 3.docx

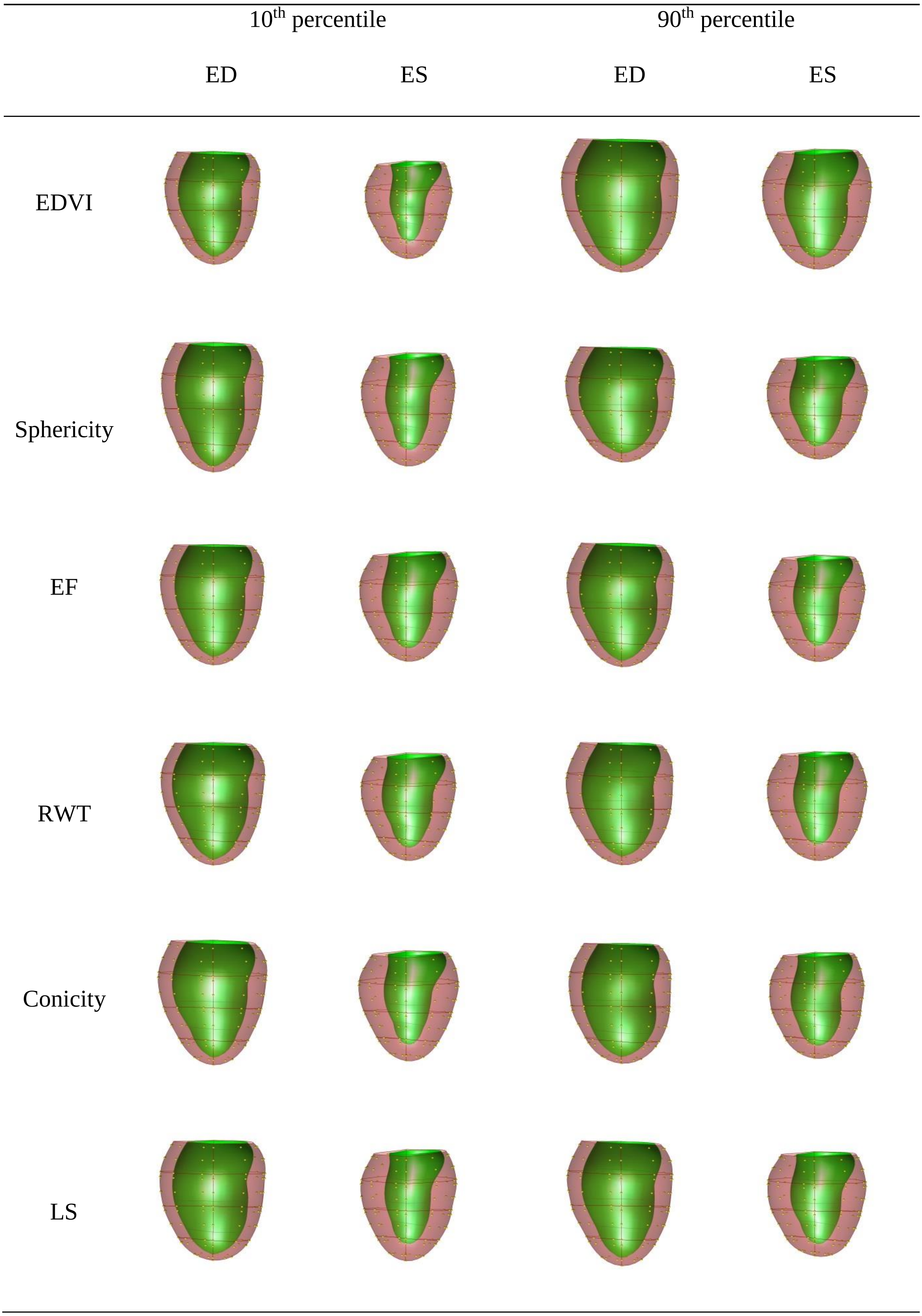

Figure 3. Plot of the PLS clinical components (M =1). Viewpoint is from the posterior with the septum on the left. EDVI= end diastolic volume index; RWT=relative wall thickness; EF= ejection fraction; LS=longitudinal shortening. ED = end-diastole; ES = end-systole. Full animations of each clinical component are shown http://www.cardiacatlas.org/tools/lv-shape-orthogonal-clinical-modes/.

Figure 4
Click here to download Figure Figure 4.docx

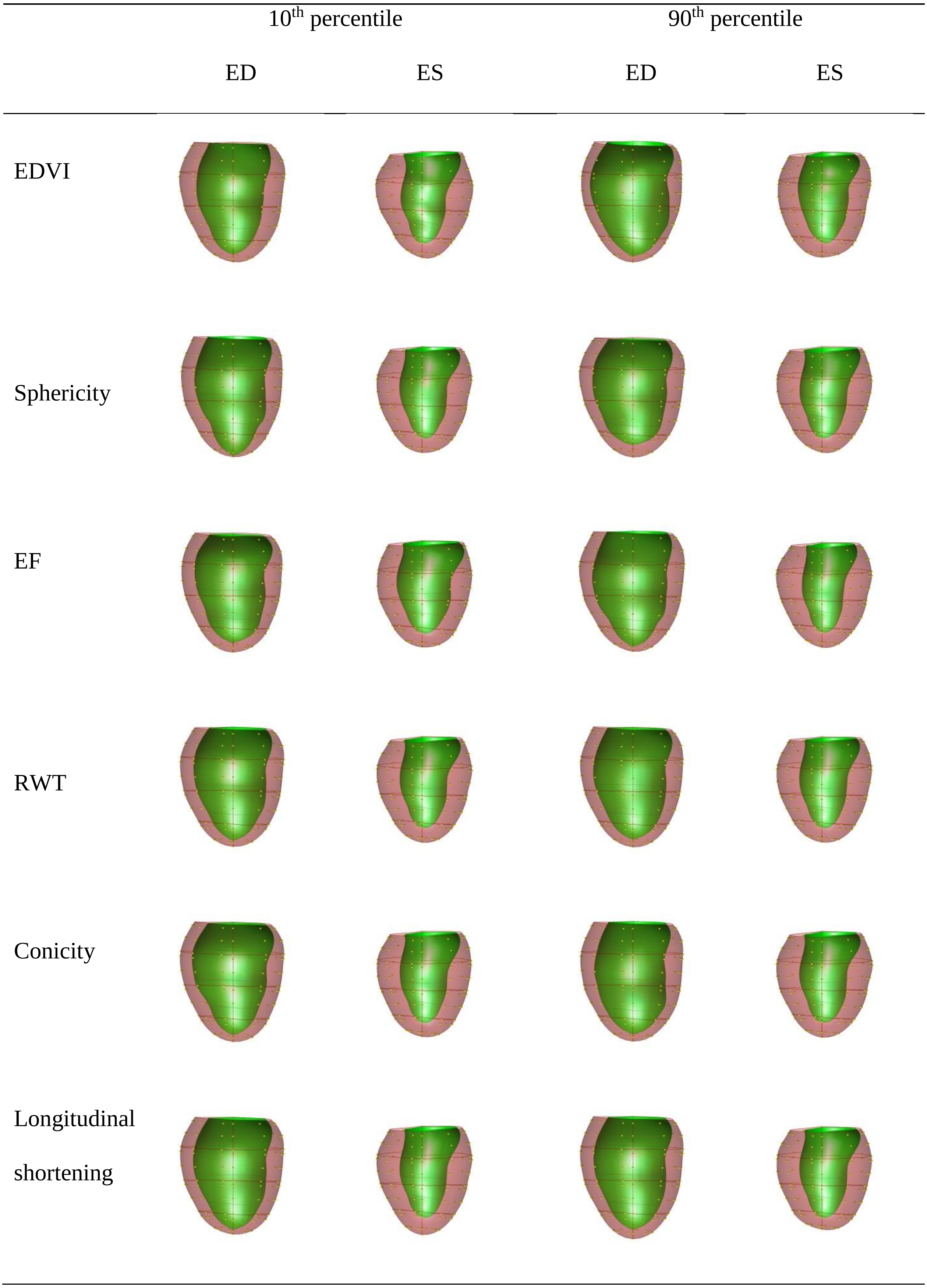

Figure 4. Plot of the PLS clinical components (*M*=10). Viewpoint is from the posterior with the septum on the left. EDVI= end diastolic volume index; RWT=relative wall thickness; EF= ejection fraction; LS=longitudinal shortening. ED = end-systole; ES = end-diastole.

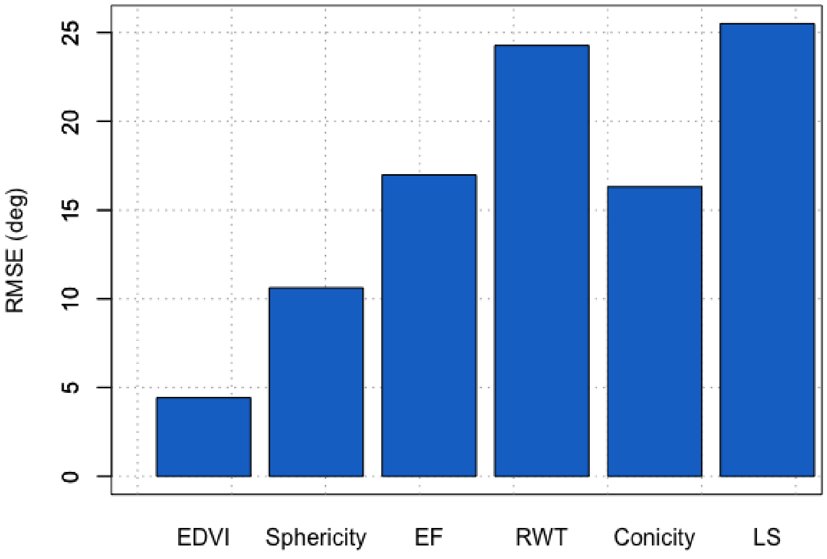

(a) Root mean squared errors between randomly sampled balanced data sets (300 ASYMP and 300 MI) and full data set (1991 ASYMP and 300 MI). Average of 50 trials.

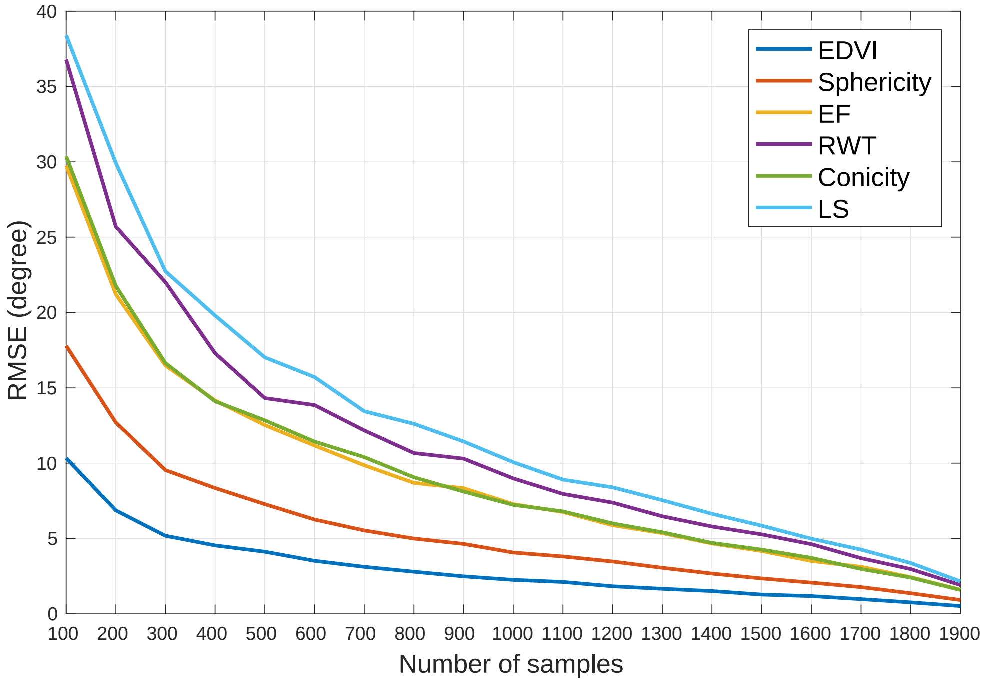

(b) Root mean squared errors varying number of asymptomatic subjects compared with the full data set (1,991 samples). Average of 50 trials.

Figure 5. Root mean squared error (RMSE) in terms of angle differences between remodelling components. EDVI= end diastolic volume index; RWT=relative wall thickness; EF= ejection fraction; LS=longitudinal shortening.

Click here to download Figure Figure 6.docx

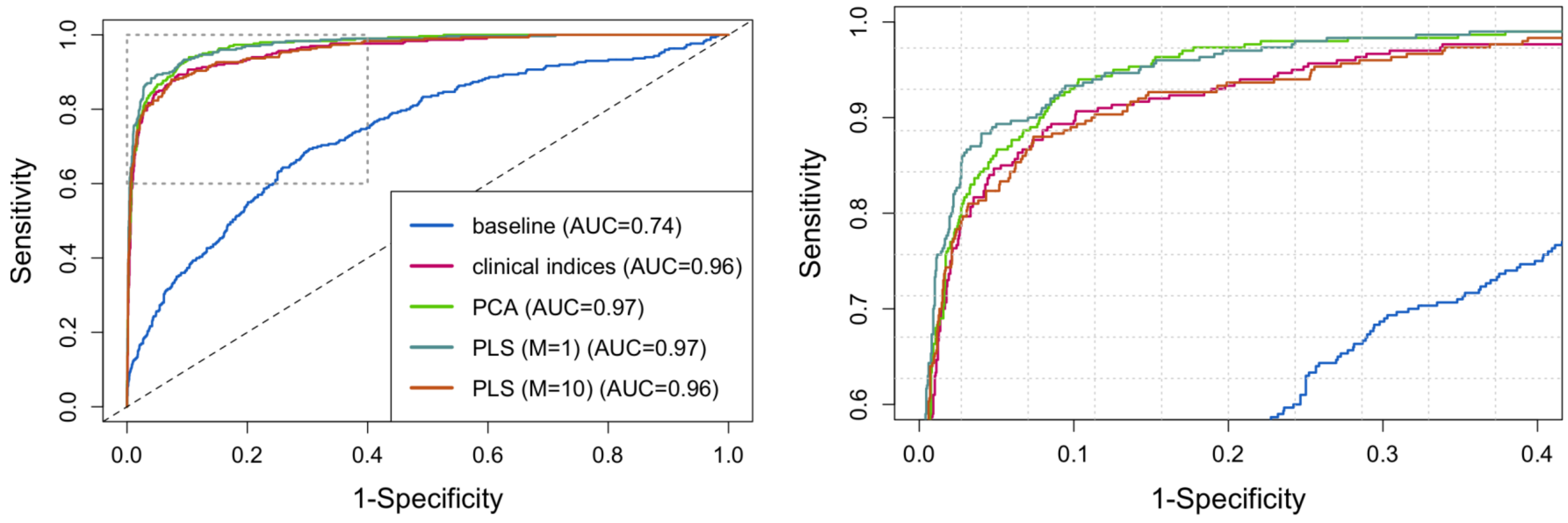

Figure 6. ROC curves for the five logistic regression models. The right figure shows a zoomed-in view to demonstrate the differences between the four models. ROC= reciever operating curve; PCA = principal component analysis; PLS = partial least squares.

Figure 7 Click here to download Figure Figure 7.docx

Asymptomatic		MI Patients	
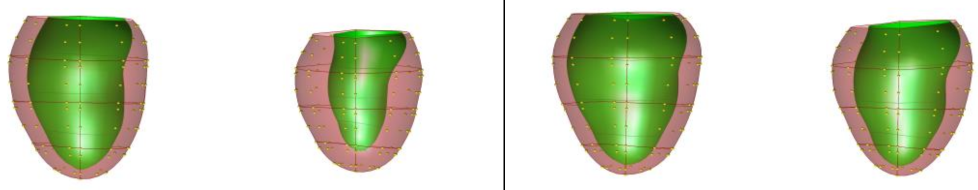			
ED	ES	ED	ES

Figure 7. Visualization of shape changes between asymptomatic volunteers and MI patients, using the combined PLS (*M*=1) components. Viewpoint is from the posterior with the septum on the left. Plots show the shapes associated with the median logistic regression score for the asymptomatic and MI patient groups respectively. MI patients show larger ventricles, less ejection, and thinner walls. MI= myocardial infarction; ED = end-diastole; ES = end-systole.

Response to Reviewers Click here to download Personal Cover PLS Review R2.docx

Reviewer #1: The rebuttal is very thorough and well annotated, which made it easy to track back the comments and modifications. The authors have made significant efforts to address all comments and as a result I believe this work is suitable for publication in this journal.

I have some very minor comments:

R1.1

The revised wording in the abstract (line 39) is still confusing without having read the manuscript and understood the methods. I would suggest the authors change to something like "A one-factor PLS regression led to more de-coupling between the clinical indices with respect to the shapes, where there was no correlation with subsequent remodelling indices". This is the most significant contribution of the work and should be very clear for the reader.

We have changed this sentence to read "A one-factor PLS regression led to more de-coupling between scores from the different remodelling components across the entire cohort, and zero correlation between clinical indices and subsequent scores."

R1.2

Line 205 - the authors should specify what "most" means here

This sentence has been modified to read: "Standard 10-fold cross-validation was performed to test estimation error, showing that the mean squared error in estimating $\boldsymbol{Y}$ did not substantially improve after 10 latent factors."

R1.3

Some discussion is needed to explain why the upper triangle in Table 2 is all zeros, and why this is not the case for the M=10 regression. This is to me a very surprising result and intuitively I don't see why this would be the case, especially for one regression and not the other.

We have added the following to the Discussion: "…resulted in zero correlation between component scores and previously removed indices (upper triangle of Table 2). This result is a feature of one-factor PLS applied in this context. One-factor PLS computes a single latent factor which maximizes the cross-correlation between $\boldsymbol{X}$ and $\boldsymbol{Y}$. The resulting remodelling component is a vector in the same direction as this single latent factor (in fact $\boldsymbol{\beta} \propto \boldsymbol{X}^T\boldsymbol{Y}$). Subtracting this component from the shape space leads to zero correlation between the residual shapes and $\boldsymbol{Y}$. For multi-factor PLS, the resulting remodelling component is a combination of all the latent factors, and no longer has this property."

R1.4

Figs 3,4, and 7 should be annotated with the image views (septal wall, free wall, base, apex)

We have added the following to the figure legends: "Viewpoint is from the

posterior with the septum on the left.”
