# Supplementary material for: Orthogonal decomposition of left ventricular remodeling in myocardial infarction
Source: Gigascience. 2017 Feb 6;6(3):1–15. doi: 10.1093/gigascience/gix005 (PMC5791439; doi:10.1093/gigascience/gix005)
Supplement: Response_to_reviewer_comments_Original_Submission.pdf [file gix005_response_to_reviewer_comments_original_submission.pdf]

Reviewer #1: General comments:
"I appreciate the large effort from this group to share data and code to help advance progress in the research community. It is also nice to see analysis of large populations."

"Overall, I find the manuscript well written and concise. However, some of the methods and motivation are still unclear to me, and due to this I have some major concerns with the methodology and results, as summarized and further detailed below."

R1.1
"My main concern with this work is with the methods. Some of the results are not consistent with my experience with PLS (and SIMPLS). Based on looking at the code, it seems that the 'pc_scores' that are computed in 'GenerateOrthogonalModes.m' are actually the prediction of Y and not the 'scores' T. I believe this could be the reason why there are unusual results for the variance of the 10-component model plotted in Fig. 4, because the incorrect scores were used (the PCTVAR output of plsregress should be what is plotted). "

We have redefined the terminology to distinguish clearly between “shape components” (i.e. PCA shape components or PLS XLOADINGS returned by MATLAB’s plsregress function) and our “remodelling components” which we define to be the normalized vector in shape space calculated from the regression coefficients. Similarly we distinguish between “shape scores” (i.e. XSCORES returned by plsregress), response scores (YSCORES from plsregress), and our “remodelling scores” which we define to be the projection of the shapes onto the remodelling component. We have included an Appendix to clarify this formulation, using both PCA regression and PLS regression to illustrate the method. Using centered data X and response vector Y, we show that the normalized regression coefficients can be thought of as a vector in shape space (column space of X) such that the projection of the shape onto this vector best explains the response. We call this vector the “remodeling component” by analogy to PCA shape components, which are also vectors in shape space. The difference is that the remodelling component is more directly related to the response variable (i.e. clinical remodelling index in our application). In the appendix, we first derive PCA shape components and PCA scores. We then show how PCA regression can be used to calculate remodelling components (by analogy to shape components) and remodelling scores (analogous to PCA scores). The remodelling components derived from PCA regression are linear combinations of the PCA shape components. Then we show how PLS regression can be used to calculate (different) remodelling components. Here the remodelling components are derived from both XLOADING and YLOADING in plsregress. For a given number of latent factors in PLS, and the same number of principal components in PCA, remodelling components derived from PLS regression always explain more of Y than remodelling components derived from PCA regression.

The orthogonalization part of our algorithm can then be applied to either PCA or PLS remodelling components, by subtracting the projection of the shapes onto the remodelling component (giving a residual data matrix), in sequence. This procedure gives an orthogonal set of remodelling components, each related to their associated clinical indices, but forming an orthogonal basis for the shape space. By analogy to PCA shape components, these orthogonal remodelling components can be used as a shape decomposition, but unlike PCA shape components, the orthogonal remodelling components have clear clinical interpretation in terms of clinical remodelling indices.

We have also changed the names of some variables in the code to be consistent with this terminology.

For discussion on Figure 4 see point 1.31 below

R1.2
"In addition, there is a strong emphasis placed on the computed latent variables being "de-correlated". In my experience, when one computes PLS for a given factor, the first component will maximise the covariance

between X and Y, but not 100%, meaning that subsequent shapes will also have some correlation with other Y - e.g. EDVi score has -0.75 correlation with EF, so this shape does not seem 'de-correlating' at all (if I understand what the authors mean by 'de-correlating'. In fact, usually ~10 components still capture some correlation with Y. Removing the first component that was computed to maximise covariance with e.g. EDV will remove some amount of EDV-related shape, but not ALL of it, which is what seems to be implied from the phrasing used in the manuscript. Therefore, despite the fact that the model with 10 latent variables yielded lower performance, it seems more "de-correlating" than the model with 1 latent variable, because the shape features related to the first variable have been more "completely" removed. However, my intuition is that removing the first 10 EDV-related shapes probably removes most of the variability of the shape from the population, since within those shapes there are some features that are also related to the other variables. So, I would think that a 1-component method is more suitable with this approach."

We have revised the terminology to clarify that our orthogonalization method using one-factor PLS regression gives “less correlated” remodelling scores than using multi-factor PLS (new Tables 6 and 7). Also it leads to “less correlation” between the remodelling scores and their associated remodelling indices (Tables 2 and 3), and “zero correlation” between the scores and the indices associated with previously removed remodelling components. Initially, it was not obvious to us that a single latent factor would lead to less correlated remodelling scores, but we have tested this behavior in several datasets and it appears to be a fundamental result, due to the fact that one-factor PLS is closer to PCA. The deJong paper also mentions a result linking one-factor PLS with ridge regression (included in the Appendix).

We have added a section in the motivation on why it is sometimes desirable to have less correlated remodelling scores, for example if the scores relate to underlying processes, then low correlation between scores implies that the processes have different effects within the population.

R1.3
"Regarding the comparison of methods and results, I don't find a convincing improvement of using PLS as opposed to PCA, in terms of accuracy or prediction. I do, however, agree that for interpretability of the results there is added gain of using this method. Therefore, I believe the idea of using PLS is valid, but the motivation for using it needs to be shifted in the paper."

Yes, it is the clinical interpretation of the components that is the main advantage of our method. We have now emphasized this in the Discussion. However, both PLS regression and PCA regression can be used to derive remodelling components. If sufficient principal components are used in PCA regression, and sufficient latent factors in PLS regression, they will give similar accuracy of prediction (and usually similar regression coefficients and therefore remodelling components). However this results in remodelling scores which are more highly correlated. In the logistic regression experiments, we show that one-factor PLS derived remodelling scores are as effective as PCA shape scores in characterizing remodelling in patients, but the advantage of the PLS remodelling components is the interpretation of each component corresponding with its clinical index.

Detailed comments:
Abstract:

R1.4
"I am not convinced that a "novel method" is proposed, as stated in the abstract. Perhaps I have misunderstood the methods but they seem to be the same as previously proposed methods using the method described in [24] and applying to the data described and previously analysed in [13]. In my opinion, this work is the application of existing methods to a data-set and should be stated as such."

The method described in [24] used a different method to derive remodelling components. For each clinical index, this previous paper defined a subset of cases outside two standard deviations of the mean, i.e. those that display very high and very low values of the clinical index. The remodelling mode was then derived

from these cases, ignoring the majority of cases between two standard deviations of the mean. The problem with this method is that it relied on extremes of the distribution of the clinical index. This may lead to difficulties in the interpretation of the remodelling component. The novel contributions of the current paper are i) calculation of remodelling components directly from regression coefficients, ii) use of the entire distribution of the clinical index to formulate the remodelling component, and iii) reduction of correlation among resulting remodelling scores, using PLS regressions with a single latent factor. These points have been added to the motivation section.

The dataset used in this work is available from the Cardiac Atlas Project, and has been used in a number of studies including [13] (now ref 12). This is the advantage of having widely-available datasets for algorithm development.

R1.5
"What is meant by "a single PLS hidden variable"? I'm perhaps not familiar with this terminology, but is this referring to a single PLS latent variable or single PLS component?"

We have changed this to “latent factor” throughout. This refers to the number of components in the PLS decompositions, i.e. the NCOMP parameter of plsregress function for the Matlab version and plsr function for the R version. There are several names for this in the literature, e.g. latent component, latent factor, latent variable, and hidden variable, etc. We chose latent factor to distinguish these from shape components or remodelling components, etc.

R1.6
"I also didn't exactly understand what is meant by a "decorrelation between scores". Is this referring to the orthogonalisation of the scores or reduction in the correlation of scores?"

See R1.2

Introduction:
R1.7
"Is there a difference between "LV volume index" and "LV volume", or is this referring to indexed LV volume? (line 55)."

End-diastolic volume index (EDVI) is the EDV divided by body surface area, defined in the Data Description section on Clinical Remodelling Indices.

R1.8
"It could be useful for the reader to define what is an orthogonal decomposition of shape (line 64)."

This is now defined in the Appendix. Also we have added more motivation of the usefulness of an orthogonal decomposition in the Background section, which includes the definition of orthogonality.

R1.9
"Line 79 - I think it may be more correct to state that PCA components are not designed to be related to clinical factors (though this can be the case). Clinical interpretation is not so much difficult, as it is suboptimal (in fact it is easy using PCR)."

We have changed this to read: “However, PCA shape components are not designed to be related to any

particular clinical remodelling index, and the clinical interpretation of PCA shape components is often difficult. Previous work showed that, LV PCA shape components did not have clear clinical interpretation beyond the first two [12]. This is a common problem with PCA shape components, since they are designed to efficiently characterize shape variation without regard to possible underlying mechanisms of disease processes."

R1.10
"Line 91 - as mentioned above, the term "PLS hidden variable" is unclear to me, could the authors clarify what exactly is meant by this (i.e. what is "hidden")?"

Changed to "latent factor", see R1.5.

R1.11
"Last sentence page 4 - is this to say that there is no possible relationship between a clinical index and a previous shape? This phrasing "complete decorrelation" seems a bit strong to me."

See R1.2

Methods:
R1.12
"General question: I'm curious to know why the authors didn't use the PLS regression coefficients directly since that is what PLS was mainly developed for (e.g. following the tutorial in Matlab on PLSR and PCR). Can the authors mention why they chose logistic regression instead? Was a comparison performed? Did it improve the results? Would we expect a logistic relationship over a linear one? Please clarify."

The logistic regression was used to evaluate the ability of the remodelling components to characterize shape changes due to myocardial infarction. PLS could be used for this task (and was used for this purpose by Lekadir et al in [28]). However we decided to use the more common logistic regression since it is the standard method used in many previous clinical research studies and is it simple to calculate relative strengths of associations using odds ratios. These comments have been added to the Characterization of myocardial infarction section.

R1.13
"General comment: It would be useful to clarify for the reader (especially those not familiar with latent variable models), what the component, loading, and scores are (i.e. component = loading x score)"

These are explained in the Appendix.

R1.14
"Line 103 - typo? should it be "heart failure or atrial fibrillation"?"

Thanks, fixed.

R1.15
"Line 112 - presumably Simpson's rule was applied? A citation here for clarity would be useful."

Actually numerical integration of the polygon formed by the surface points. Citation added.

R1.16
"Line 154 - perhaps deflation could be defined here. Deflation is typically used in original PLS algorithms but not SIMPLS, thus it could be nice to differentiate between standard 'deflation' and the orthogonalisation process used here."

We have avoided use of deflation in this context. We have used “residual data matrix” instead.

R1.17
"N_latent was described before being introduced (page 6). I think the equation for maximising the covariance between T and U should be added here, and it should be mentioned that this constraint is what distinguishes PLS from, for example, PCA (i.e. this is how the shape modes are computed to maximise the variance in Y). The formula for B should be provided. Y_residuals is not defined."

These concepts are now explained in the Appendix. The relationships between T, U and B are not easy to write in closed form and actually change with particular implementations of PLS, so we have simplified this section.

R1.18
"Line 153 - "this step ensures orthogonality" with respect to what?
Presumably with respect to B but this is not explicitly stated."

This is now explained in the section next to equation 3.

R1.19
"Line 162 - the term "PLS component" is introduced here to refer to the normalised regression coefficients B_i. Please consider another term to avoid confusion e.g. with 'component' as is used in PCA."

See R1.1.

R1.20
"Page 8 - why was 10 chosen as the upper limit for the number of latent variables?"

We have included a plot of the mean squared prediction error with a 10-fold cross validation (Figure 2). We have added the following to the “Number of latent factors” section: “Standard 10-fold cross-validation was performed to test estimation error for multi-factor PLS, showing that 10 latent factors accounted for most of the mean squared error in estimating Y (Figure 2).”

R1.21
"Page 8 - The authors claim that there is no standard method to choose the number of latent variables. Cross-validation could typically be used for this, as mentioned in the Matlab tutorial for PLSR and PCR. For such an investigation it would be nice to compute and plot the leave-one-out or split-half errors for the number of latent variables = 1:299 (number of subjects - 1), and then just the optimal errors could be reported."

See R1.20

R1.22
"Line 172 - it could be useful to mention why X^k+1 is orthogonal to B^k."

This has been made more explicit next to equation 3.

R1.23
"Line 183 - details on the logistic regression technique and how this was performed could be added (stepwise forward logistic regression? SPSS?)."

This is described in the new section about Statistical Analysis.

R1.24
"Line 186 - BMI and SBP should be defined here."

Thanks. We have made an explicit definition of these terms, including in each table caption.

R1.25
"Line 187 - it would be nice to mention why these were chosen as the baseline variables and why baseline variables were included."

In the original paper, we used covariates commonly used in the literature as baseline variables in the logistic regression models. However, we have now rationalized the choice of baseline variables to those in Table 1 with significant differences between asymptomatic and MI groups. Smoking was also included since this is known to have a significant effect on the heart. The logistic regression experiments were updated accordingly.

R1.26
"Line 188 - Why was a 6 component PCA model used? According to [13] this model only represents ~75% of the shape variance in the population."

We used six PCA components in the logistic regression analysis because we only used six remodelling components. Incorporating more components is expected to give better results. However, these results show that with the same number of components, orthogonal remodelling components perform as well as PCA (and the original indices), but with the advantage that the remodelling components have clear clinical interpretation, while maintaining the property of orthogonality.

R1.27
"Line 202 - is ESV used without indexing? If not, LVESVi should be used. If yes, why was EDV indexed and not ESV?"

We have deleted this, since ESV was not included in Table 1, or in the clinical remodelling indices. This is because ESVI can be inferred from EDVI and EF.

Analyses
R1.28
"Line 199 - Please add the statistical significance threshold ($p < 0.05$), or to avoid repetition, just state once at the beginning of this section that statistical significance was set at $p<0.05$."

This has been included in the section about Statistical Analysis.

R1.29
"For reproducibility purposes it could help the reader to mention which software (if any) was used to perform the statistical analyses"

This has been included in the sections on Implementation and Statistical Analysis.

R1.30
"Line 222 - could the authors elaborate on this sentence, I didn't get what is meant by 'retaining correlation with the index', and why this would be a bad thing"

This sentence has been removed for to avoid confusion.

R1.31
"Line 226 - I am very surprised to see that only 15% of the shape variance in the population was captured by 6 components from the N=10 model. Again, perhaps I have misunderstood, but my understanding based on the description of the methods is that the 10-component model should have 10-components for EDV, 10 for sphericity, and so on, so there should actually be 10 x 6 = 60 components for this model, and therefore I would expect a much larger amount of the variance to be captured in such a model. Could the authors clarify why this is not the case, or please correct me if I am wrong about the methods. "

Figure 4 in the previous manuscript version was calculated as the variance of the remodelling scores, divided by the total variance in the data matrix (trace(XTX)/(N-1)). This was done because we are using the regression coefficients as the remodelling component, not the PLS components themselves. This result follows from the fact that a single latent factor results in a remodelling component that is influenced by variance in X as well as Y. However this is peripheral result and, for clarity, we have removed this figure in the revised manuscript to avoid confusion with variance explained returned from PLS regression algorithm which can be either for predictor or response variables.

R1.32
"Line 246 - presumably 'LR' stands for logistic regression? Could you add this to the text and figures"

We have removed the acronym for logistic regression for clarity.

R1.33
"Line 246 - why was the median chosen? Please mention briefly here."

Median shapes were estimated since this is more robust to outliers in general. However in this case mean shapes give similar results.

R1.34
"Line 250 - how are the baseline variables adjusted? Does this significant change the shapes? (This question is more out of curiosity than actually needing clarification)"

This means that the baseline variables were included in the logistic regression models as covariates. LR models are often termed “adjusted” by these covariates.

Discussion
R1.35
"Line 266 - as mentioned previously, I would rather state that an orthogonal PLS framework was applied, without implying that there are new methods proposed in the present work. Again, if this is not the case, please clearly describe the contributions of the present work and distinguish how this method differs from other orthogonal PLS methods."

See R1.4 for an explanation of the novel contributions of the paper.

R1.36
"Line 273 - orthogonality was described here, but should also be mentioned at the beginning of the methods section."

This is now defined in the second paragraph of the Background section.

R1.37
"Line 274 - I got a bit lost here with the terminology, are the "PLS shape components" referring to loading x score or are you referring to the loading (which I guess is the case because PLS loadings are orthogonal)? And presumably "PLS shape component score" is referring just to the scores (which are not necessarily orthogonal for PLS)? Here there is also the mention of the term 'decorrelated', should that be 'orthogonal'?"

We have rationalized the terminology- see R1.1 and R1.2.

R1.38
"Line 284 - there is again the use of 'decorrelation' and I just now think I understand what is meant by this. Perhaps "reduction/decrease in correlation" is clearer?"

See R1.2.

R1.39
"Line 285 - I'm honestly very surprised to see "total decorrelation" (and again, I would suggest using "zero correlation" rather than "decorrelation") between the PLS scores and clinical indices. Indeed this suggests that the 1-component model is able to remove any relationship with EDVi (for the second component), and so on."

We have changed this to zero correlation as suggested.

Results
R1.40
"In all results (and tables, figures) it would be useful to clarify when experiments are including both populations and when it is MI only, sometimes I got confused by that."

All experiments and Tables show results including all cases (both asymptomatic and MI patients), unless explicitly stated.

R1.41
"I'm not sure how to interpret the results. Are the authors looking for the most predictive model? In that case I would expect to see a more thorough analysis of the number of latent variables (using cross-validation)."

The logistic regression experiments were performed to examine the ability of the orthogonal remodelling components to characterize shape changes between patients with MI and asymptomatic volunteers, compared with the same number of PCA components, or the original clinical indices themselves. The interpretation of the results is that orthogonal remodelling components are able to characterize differences between groups with similar metrics to PCA or the original indices, but with the added advantage of having a clear clinical interpretation and maintaining orthogonality. We use the AIC etc as metrics of “goodness” in this context with respect to traditional PCA shape components. The question of how many latent factors is a separate issue, and we show that one-factor PLS remodelling components perform better than multi-factor PLS in the logistic regression experiments in all metrics (Table 9). We have also included a cross-validation to show that 10 factors is a reasonable choice for the multi-factor case, in terms of prediction of the response variables, but this is another issue again.

R1.42
"Do the authors have some reasoning for why LS score was significant with the 1-component model and not the 10-component model, and vice versa for conicity?"

This was a typo, thanks. The results have changed somewhat with the revised logistic regression analyses (DBP was include as a covariate rather than SBP). The one-factor model shows different significant scores from the multi-component model. We believe this is due to the increased multi-colinearity in the multi-factor model.

Tables
R1.43
"In all tables it would be useful to include the abbreviations"

We have included all abbreviations in all table captions.

R1.44
"The tables are in general very content-heavy, and it's not easy to see what the take-home message is from each table. Some additional annotations or descriptions in the legend would help guide the reader to interpret these results. For example, the statistically significant components in Table 8 could be highlighted for easy readability, rather than using an asterix."

Comments on the interpretation of results have been added to the Results text where the Tables are cited. We have used bold in Table 8 as suggested.

R1.45
"Are Tables 2-7 showing results for the MI population only or are these combined results for both populations (please specify in the legends)."

Unless otherwise specified all Tables show results including all cases combined. This is now made explicit at the beginning of the Results section.

R1.46

"In Table 8 it would be useful to include some descriptions of what are "good" values in terms of the coefficient, error, OR and CI."

These have standard interpretation in the clinical literature, and depend on the units of measurement. In general, higher means more influence on the dependent variable.

R1.47
"Table 9 is a nice summary of the results and easy to interpret. Line 195 could be repeated here to remind the reader what is preferred for each measure (e.g. >AIC = better)"

Done.

R1.48
"Table 5 and 6 - it is not clear what is meant by 'PLS clinical mode scores' and how this is different from 'PLS component scores'."

This has been rationalized, see R 1.1

R1.49
"Table 7 should be moved to follow Tables 2,3 for easier readability."

Table 7 has been shifted to become Table 4.

Figures
R1.50
"In all figures it would be helpful to include the abbreviations"

Done.

R1.51
"Figure 1 is nice and clear. If possible, it could be useful to include on the left-hand side an image depicting each measure or the formula for computing each measure, and on the right-hand side the corresponding modes at +1SD. X6 could be pointing downwards for consistency"

We thought this would clutter the figure and make it less readable. This information is repeated in Figure 3 and in the text.

R1.52
"I don't find Figure 2 and Figure 3 very informative in the sense that I don't know what I should conclude from these images. Perhaps some annotation could help as guidance. It would be nice to have some interpretation and comparison of the modes in Figure 2 and 3 to the modes in Figure 14 of [13] in terms of highlighting for the reader regions of interest or interesting behavior that is visible from these modes (i.e. what should we, as readers, take from these Figures?)"

These figures visualize the remodelling components associated with the clinical indices. These visualizations are useful in understanding the effect of each component on shape. This explanation has been added to the text near first mention of the figures. Animations of these remodelling components from the smallest and to the highest percentiles can clearly visualize how these components are associated with the clinical indices. The animations for the single latent factor are shown interactively on the Cardiac Atlas Project website: http://www.cardiacatlas.org/tools/lv-shape-orthogonal-clinical-modes/. We added this link to the figure

caption.

R1.53
"The labels on the x-axis of Fig. 4 are a bit misleading. I would rather put 'PC1' directly below the blue column, and EDVI PC below the red/green columns (since PC1 in PCA is not related to EDVI, or am I mistaken?)."

This figure has been removed. See R1.31.

R1.54
"Figure 4 - I am very confused by these results, especially for the first component. To my understanding, in both the 1-component and 10-component models, PLS was performed with the same X shape features and EDVI as the Y variable. There is no tuning of SIMPLS to force all of the variance to be in the N-components, therefore the variance of the first component should be equal, regardless of the number of components that was chosen. The number of chosen components changes the accuracy of the regression, but not the components themselves. Therefore, the variance of the first component should be much higher than what is reported for the 10-component model. While there would be large differences in the subsequent components (because there is much fewer variance in the other components for the 10-component model because so much of the shape has already been removed from X^k), the first component should be identical to the 1-component model (i.e. 50%). Please clarify why this is not the case."

Hopefully, this it should now be clear that Fig 4 was plotting variance in remodelling scores. However, to avoid confusion, we have removed this Figure (see R1.31).

R1.55
"Figure 5 - The improvement from baseline alone is clear (and expected) but I don't see a dramatic improvement based on the figures for the shape-based models and using clinical indices alone. Moreover, there isn't a clear improvement above PCA. Figure 5- could the authors add AUC (as reported on line 239) to the figure?"

The ROC curves are now in Figure 6. We have added the AUC values in the legend. We tested whether the AUC values of single and multi latent factor PLS models are significantly different from the PCA and clinical index models. The test showed that AUC of the single latent factor PLS model is significantly greater than using clinical indices alone, but not different to the PCA model. However, the multi latent factor PLS was significantly smaller than PCA, but it was not significantly different than clinical index model. The interpretation of this Figure is that the single latent factor (M=1) orthogonal remodelling components give similar performance to PCA, but with the added advantage of clear clinical interpretation of the components. These observations have been added to the Discussion section.

R1.56
"Figure 6 is interesting. Perhaps the author could consider adding some annotation to guide the reader about the shape differences (e.g. there seems to be less systolic contraction in the MI patients) and a summary of what to conclude in the legend (even a repetition of line 249 would be helpful here)."

Done.

Tools:
R1.57
"For the sake of this journal (being focused towards open-source tools),I would suggest that the authors use R
(https://cran.r-project.org/web/packages/pls/index.html) instead of Mxatlab, to avoid the need for users to

purchase a Matlab license. Using the plsregress function also requires a license for the Statistics and Machine Learning Toolbox.
I am not familiar with Giga science, but based on the website it is stated that all research objects are published (data, software tools, and workflows). In order to reproduce the results from this study (or indeed to apply the methods to new data), the community would need to have access to the image processing tools that were used to extract the models. PCA (or similarly PLS) applied to data that has already been extracted and parameterised is straightforward using existing software (or indeed using built-in Matlab, python, or R functions). While it is a useful resource to have access to the images and the models extracted from these images, the biggest challenge we face in the field is in creating the models to be able to perform the analysis."

We have included code in R in the GitHub repository, which is linked from http://www.cardiacatlas.org/tools/lv-shape-orthogonal-clinical-modes/ webpage.

Code for creating the shape models is provided at http://www.cardiacatlas.org/tools/. This code is offered as is where is, and we have not been able to ensure that the dependencies are available.

Novelty:
R1.58
"As mentioned previously, to my knowledge this technique has already been described in [24] and there is inadequate referencing to previous techniques. Orthogonalisation using the Gram-Schmidt method has been discussed earlier, for example Izenman, A.J., 2008. Modern multivariate statistical techniques (Vol. 1). New York: Springer, page 570), and for PLS specifically: de Jong, S., Wise, B.M. and Ricker, N.L., 2001. Canonical partial least squares and continuum power regression. Journal of Chemometrics, 15(2), pp.85-100. Moreover, the Matlab code for canonical (i.e. orthogonal) SIMPLS is provided in this paper."

See R1.4. The De Jong 2001 paper shows how to derive PLS regression from a canonical decomposition, which is not the same as the orthogonal remodelling components derived in our paper.

Code:
R1.59
"It isn't clear to me why the regression coefficients ('Beta') are normalised in 'GenerateOrthogonalModes' and subsequently why the scores and loadings from the plsregress function are not used directly. Could the authors explicitly mention why this normalisation is important."

See R1.2. Normalized regression coefficients (without the intercept) give rise to a unit vector in shape space which can be used as a component similar to shape components in PCA.

R1.60
"As mentioned previously, from what I understand from the code, the 'pc_scores' are computed as X*B (data matrix X times the regression coefficients). However, this is the model of Y, not the computation of the scores. The scores T would usually be computed by projecting X onto the loadings P."

See R1.2. Remodelling scores can be used to calculate estimates of Y but are also scores associated with remodelling components.

Reviewer #2:

R2.1
"General: The strength of this paper is the novel mathematical process of making decoupled geometrical modes, while still correlating with clinical indices, and the main limitations is that the study is cross sectional and as such limited understanding can be gained on what really drives the remodeling. The paper is missing a limitation section where the lack of cross sectional data is highlighted and the need for such future

research is discussed."

We have included a Limitations section which includes the cross-sectional nature of the dataset, and applications to other datasets.

R2.2
"Abstract: A novel method for deriving orthogonal shape components directly from any set of clinical indices. The word any is a strong word given the mathematical depth of the paper. For instance, the clinical indices need to be reasonably well uncorrelated for the operation to be meaningful and produce shape components that do correlate with the chosen clinical indices."

We agree, although what constitutes “reasonably uncorrelated” is difficult to define and is beyond the scope of the current paper. This is likely to depend on the application and might be a matter of trial and error. We have chosen remodelling indices which are common and moderately independent (e.g. we did not include ESV since EDV and EF had larger variance). The abstract has been revised to read “We developed a novel method for deriving orthogonal remodelling components directly from any (moderately independent) set of clinical remodelling indices.”

R2.3
"Abstract. Why is not infarct size one of the clinical indices? Likely, it must be stronger than for instance longitudinal shortening to determine remodeling?"

We did not include structural indices such as % infarct mass or infarct transmurality or age in this work, since we wanted to focus on geometric remodelling indices which have been well established in the clinical literature. These indices are also available from several imaging modalities such as 3D echo and CT. While more information is becoming available on the interesting effects of infarct size and transmurality, this requires explanation of specific methodological techniques and is left for future work. We have included these comments in the Limitations section.

R2.4
"Page 3, Line 67. In this section you may lose some of the potential readers of the paper. I do understand and acknowledge that it greatly simplifies that any given metric tensor does not have off diagonal elements and is orthonormal ideally. However, is this really a practical limitation as in order to compute measures such as arc lengths and areas it simple to reconstruct the original shape of the patient and compute them directly in the Euclidean space? Well it is more computationally intensive, but it is more convenience rather than anything else?"

We have removed this sentence for simplicity. We have also included more motivation of the utility of orthogonal components (see R1.2; R3.5).

R2.5
"Page 5, line 114. The definition of relative wall thickness is rather strange, I presume that this is the form where previous researchers got significant correlations for prognostics for this parameter, but it would be good to have some more rationale on why this rather bizarre formulation, and why not for instance absolute mean wall thickness in mm (or even minimal wall thickness from thinned after myocardial infarction etc)."

The echo community has used this definition for many years since it is easy to measure from an M-mode parasternal view. Many papers have used this as a prognostic measure, and to quantify concentric vs eccentric remodelling. All the remodelling indices in this paper were defined as ratios to correct for scale in some sense. We have included more rationale for the selection of clinical remodelling indices in the Data Description section.

R2.6

"Page 6, line 119. Some more details would be good here. Is it the basal AV plane movement divided by the straight distance to apex or is it divided by the curve length? Central basal point is this the middle point of the mitral valve?"

This sentence has been modified to: “Longitudinal shortening was calculated as the difference of the distance between the centroid of the most basal ring of points to the most apical point at end-systole divided by the distance at end-diastole.”

R2.7
"Page 7, Line 156. How was the next component to be removed from the shape space determined? Greatest variance in what respect?"

Remodelling components were removed in order of variance of the remodelling scores. This is a measure of the shape variance explained by each index. There could be several methods for determining the order of the indices, and this requires further research. This has been added to the Orthogonal Remodelling Components section and the Limitations section.

R2.8
"Page 11, line 252. This paragraph is somewhat important as I understand it in terms of possible application of the technology. This section could perhaps be better explained and expanded as it deals with how the shape decomposition can be used to derive new knowledge."

We have added the following to the Potential Implications section: “Although the remodelling components were generated from a largely asymptomatic population in this work, we showed how they describe the shape changes undergone in myocardial infarction relatively well. We also showed how the amount of each remodelling component could be quantified in association with the presence of clinical disease, highlighting significant contributions of ventricular size, sphericity and relative wall thickness. These methods enable new knowledge to be derived from medical imaging examinations on the underlying mechanisms driving the adaptation of the heart in response to disease.”

R2.9
"Please when introducing new abbreviations such as LR help write them out in the text. Is it correct that LR in this context it is logistic regression?"

We have removed this abbreviation for clarity.

R2.10
"Page 14, line 306. The concept of tracking patients over time with shape decompositions should be highlighted better as this is a rather new concept at least to clinicians and how then such changes can be better understood given orthogonal bases. Please expand somewhat if possible."

We have added the following to the Potential implications section: “The resulting remodelling scores can be used to track the progression of remodelling over time, against reference populations. This would enable automatic computation of z-scores giving precise information on how the patient’s heart compares against the reference population (in this case the MESA cohort).”

R2.11
"Page 14, potential implications. Myocardial infarction is a rather broad category in terms of location, and transmurality of the infarct. Furthermore, nowhere throughout the paper it is discussed other causes of remodeling such as valvular disease. As I understood from the description of the normals they did not have valvular disease, but it is rather likely that the infarct patients had such comorbidities."

We have added the following to the Limitations section: “While more information is becoming available on the interesting effects of infarct size and transmurality, this is left for future work. Also, many patients have

comorbidities such as valvular disease, which was not examined in the current study.”

R2.12
"Page 21. Table 1. What is the "old" of the myocardial infarction, i.e how long was it between myocardial infarction and imaging. This may be highly important since that if all are fresh infarctions (< months), then rather little remodeling may have occurred such as limited wall thinning in the infarcted area etc. It is very acute then you have myocardial edema etc as well."

Most patients had stable long term myocardial infarction (none of the patients were acute). We have added this to the Patient Data section.

R2.13
"Page 22, Table 2, it is maybe worth commenting on in the text that LS and RWT achieves rather low correlations compared to their clinical indices. This is even visible in Figure 2, where the 90th percentile of LS does not really show much influence on longitudinal shortening. In fact, as I understand it as the correlation is about 0.5, then this shape mode do only explain 25% (0.5*0.5=0.25) of longitudinal shortening is this correct? How meaningful are really correlations below say 0.7(=> 50% explaining power)?"

Yes, the Pearson correlation coefficients can be low and still be significant due to the large numbers of cases. The question of clinical meaning is an open area of research. Treatments that give a small improvement in remodelling may lead to large cost savings.

R2.14
"Page 25, Table 8. What is meant with the baseline model? I find the baseline model poorly described in the paper, please provide more details."

For clarity these have been changed to “baseline variables” throughout. In the original paper, we used covariates commonly used in the literature as baseline variables in the logistic regression models. However, we have now rationalized the choice of baseline variables to those in Table 1 with significant differences between asymptomatic and MI groups. Smoking was also included since this has a significant effect on the heart. The logistic regression experiments were updated accordingly. The baseline variables are listed in the Characterization of Myocardial Infarction section. These are age, sex, body mass index, diastolic blood pressure, smoking status and diabetes history.

R2.15
"Figure 1. Is the order of the indices a design choice or is it based on data? Please expand the legend. See also comment 6."

The order of the indices is important, and we chose the order of the amount of shape variance explained by each remodelling component. This was calculated from the variance of the remodelling scores. See R2.7

R2.16
"Is it not strange that given the order EDVI, Sphericity, EF, RWT, Conicity, LS index that the correlations in Table 2 are not dropping in that order, or is this not necessary and rather reflects underlying correlation (or lack thereof) of the clinical indices. If possible, please expand on this."

We believe that the interdependence between clinical indices is a determinant of the decreasing diagonal correlations in Table 2. Thus, RWT and LS are related to indices previously removed by the orthogonalization process (RWT is related to EDVI and sphericity, LS is related to EF). They generally decrease with more components, but they don’t need to be monotonic. This has been added to the Results section.

R2.17

"Figure 4, is it possible to choice grayscale or colors that works when printed on a grey scale printer."

This figure has been removed, since it was secondary to the main message of the paper (see R1.31).

R2.18
"Figure 5, the legend does not describe what is really tested (the decompositions) power to tell if a given patient has in infarction or not? Correct? What is here meant with baseline?"

The ROC curves (now shown in Figure 6) measure the ability of the logistic regression model to characterize presence of disease, based on the remodelling components and the baseline variables. Significance tests have now been added. The baseline variables were included because they were different between cohorts and may act as covariates. This is now explained in the Characterization of myocardial infarction section.

R2.19
"What is the stability of the suggest method? You used the SIMPLS algorithm as implemented by Mathworks, would this change with another algorithm? Are there fundamental differences in possible solutions? Either perform some experiments or discuss this theoretically."

We implemented the computations in R and compared the remodelling components obtained with SIMPLS with kernel, wide kernel and classical orthogonal scores algorithms, and the results were very similar in the regression coefficients obtained.

R2.20
"The other factor that would influence the choice of subject population. Here you have 300 infarct patients and some 2000 "normals". Would you get to the same decomposition if you used another set of infarct patients and normal as well as another ratio between normals and patients? This could be tested by taking a sub-population of the input data and perform the computations and compare how these two decompositions coincide in some suitable measure. This would significantly strengthen the paper as the paper describes a rather generic approach to shape decompositions."

We ran a series of experiments with 300 patients and 300 asymptomatic volunteers, with 50 random samples (trials). The root mean squared errors (RMSE) between the resulting remodelling components and those found using all cases (expressed as an angle in degrees calculated from the dot product between the vectors) are shown in Figure 5a. Although the first remodelling component is similar, increasing differences can be seen in the other components. This was expected since the characteristics of the cases included in the training set have an influence on the results. We also looked at the remodelling components generated from the asymptomatic cases alone, increasing the size in the sample from 100 to 1900 (50 trials each). The RMSE with respect to the full 1991 asymptomatic dataset are shown in Figure 5b. This graph shows that we need about 1100 cases to get below 10 degrees in all components. The choice of training data depends on the application. In this paper we used all the available data to generate the remodelling components, since we were primarily interested in the proof of concept. In future work a balanced dataset of more than 1000 cases in each group would be ideal. This would enable calculation of the differences between “asymptomatic remodelling” and “symptomatic remodelling”, which would be of considerable interest in terms of physiological driving factors. These results and comments have been added to the Results and Discussion sections.

Reviewer #3: "This paper presents an approach to extract new shape indices from asymptomatic and infarcted ventricles such that they are orthogonal and have high prediction capability. The paper is well written and can be of interest to the statistical cardiac modeling community."

Comments/Questions:

R3.1

"PLS has already been used for myocardial infarction classification by Lekadir et al. in STACOM 2015. The authors should cite this paper and describe the differences between the two works."

We have added the following to the Discussion: "Previous studies have also used PLS to derive information on cardiac remodelling [28]. Lekadir et al [28] used PLS to characterize myocardial infarction using class labels as the response variable and the data matrix as the predictor variables. They found that running the regression with a range of latent factors and combining the estimations with a median operator could obtain better performance. In the current paper, logistic regression was used (instead of PLS in [28]) with the class labels as the response variable, because this is a commonly used clinical tool to examine associations with disease, and it is simple to calculate relative effects of the components on the response variable as odds ratios. The current paper also differs from [28] in the use of PLS to derive orthogonal remodelling components and the finding that a single latent factor reduces correlations in the resulting remodelling scores."

R3.2
"What is the difference between calculating the PLS indices based on the clinical indices (EDVI, sphericity, etc) instead of directly using the class labels (asymptomatic vs. Infarcted) as in Lekadir et al. STACOM 2015? The authors should compare the extracted PLS scores through the two methods and see if there are indeed differences."

See 3.1. The focus of the current paper was to derive orthogonal remodelling components based on clinical remodelling indices. We found PLS to be useful in this goal. For the examination of relative effects of these remodelling components on the presence of disease we preferred to use the more common logistic regression analysis.

R3.3
"The authors used an imbalance dataset to train the PLS models (300 abnormal vs. about 2000 healthy cases), which may affect the significance of the new shape indices. It would be good to verify if data imbalance affects or not the extraction of the new shape indices. I suggest that the authors run the same experiments with the 300 infarcted cases and 300 randomly selected asymptomatic cases and compare the results."

See R2.20. The choice of training data depends on the application. In this paper we used all the available data to generate the remodelling components. We have included experiments showing that different components are generated using different training data.

R3.4
"It would have been interesting to have a method that finds automatically the best order in the calculation of the PLS score, may be using some statistical criteria, instead of the ad hoc order used in the manuscript (i.e. EDVI, sphericity, EF, etc). What happens if you start with wall thickness for example, which is more directly linked to myocardial infarction?"

Yes, this would be a useful area of future research. We have included this in the Limitations section.

R3.5
"What is the clinical meaning of the extracted PLS indices? How can they be used by clinicians? Can you show some figures illustrating the variation induced by these indices and their clinical meaning? How do these indices describe better remodeling or infarction than standard clinical indices?"

We have included more motivation in the Background section and also expanded the Potential implications section. The main advantage of the remodelling scores generated by the proposed method is that they have clear clinical interpretation with respect to their corresponding clinical indices, as well as being an orthogonal decomposition of shape space.
