# Supplementary material for: Orthogonal decomposition of left ventricular remodeling in myocardial infarction
Source: Gigascience. 2017 Feb 6;6(3):1–15. doi: 10.1093/gigascience/gix005 (PMC5791439; doi:10.1093/gigascience/gix005)
Supplement: Response_to_Reviewer_Comments_Revision_1.pdf [file gix005_response_to_reviewer_comments_revision_1.pdf]

Reviewer #1: The rebuttal is very thorough and well annotated, which made it easy to track back the comments and modifications. The authors have made significant efforts to address all comments and as a result I believe this work is suitable for publication in this journal.

I have some very minor comments:

R1.1
The revised wording in the abstract (line 39) is still confusing without having read the manuscript and understood the methods. I would suggest the authors change to something like "A one-factor PLS regression led to more de-coupling between the clinical indices with respect to the shapes, where there was no correlation with subsequent remodelling indices". This is the most significant contribution of the work and should be very clear for the reader.

We have changed this sentence to read “A one-factor PLS regression led to more de-coupling between scores from the different remodelling components across the entire cohort, and zero correlation between clinical indices and subsequent scores.”

R1.2
Line 205 - the authors should specify what "most" means here

This sentence has been modified to read: “Standard 10-fold cross-validation was performed to test estimation error, showing that the mean squared error in estimating Y did not substantially improve after 10 latent factors.”

R1.3
Some discussion is needed to explain why the upper triangle in Table 2 is all zeros, and why this is not the case for the M=10 regression. This is to me a very surprising result and intuitively I don't see why this would be the case, especially for one regression and not the other.

We have added the following to the Discussion: “…resulted in zero correlation between component scores and previously removed indices (upper triangle of Table 2). This result is a feature of one-factor PLS applied in this context. One-factor PLS computes a single latent factor which maximizes the cross-correlation between X and Y. The resulting remodelling component is a vector in the same direction as this single latent factor (in fact $\beta \propto X^T Y$). Subtracting this component from the shape space leads to zero correlation between the residual shapes and Y. For multi-factor PLS, the resulting remodelling component is a combination of all the latent factors, and no longer has this property.”

R1.4
Figs 3,4, and 7 should be annotated with the image views (septal wall, free wall, base, apex)

We have added the following to the figure legends: “Viewpoint is from the posterior with the septum on the left.”
