# Supplementary material for: Orthogonal decomposition of left ventricular remodeling in myocardial infarction
Source: Gigascience. 2017 Feb 6;6(3):1–15. doi: 10.1093/gigascience/gix005 (PMC5791439; doi:10.1093/gigascience/gix005)
Supplement: Reviewer_1_Report_(Original_Submission).pdf [file gix005_reviewer_1_report_(original_submission).pdf]

Reviewer Report

Title: "Orthogonal Decomposition of Left Ventricular Remodelling in Myocardial Infarction"

Version: Original Submission **Date:** 8/8/2016

Reviewer name: Kristin McLeod

Reviewer Comments to Author:

General comments:

I appreciate the large effort from this group to share data and code to help advance progress in the research community. It is also nice to see analysis of large populations.

Overall, I find the manuscript well written and concise. However, some of the methods and motivation are still unclear to me, and due to this I have some major concerns with the methodology and results, as summarised and further detailed below.

My main concern with this work is with the methods. Some of the results are not consistent with my experience with PLS (and SIMPLS). Based on looking at the code, it seems that the 'pc_scores' that are computed in 'GenerateOrthogonalModes.m' are actually the prediction of Y and not the 'scores' T. I believe this could be the reason why there are unusual results for the variance of the 10-component model plotted in Fig. 4, because the incorrect scores were used (the PCTVAR output of plsregress should be what is plotted). A simple test in Matlab highlights this:

```
clear

nObs = 20; % Assign the number of observations

nParam = 30; % Assign the number of parameters

% Generate the simulation data

X = nParam * rand(nObs,nParam) .* sign(rand(nObs,nParam) - 0.5);

Y = nParam * rand(nObs,1);
```

```
[XL1,YL1,XS1,YS1,BETA1,PCTVAR1,MSE1,stats1] = plsregress(X,Y,1);

[XL10,YL10,XS10,YS10,BETA10,PCTVAR10,MSE10,stats10] = plsregress(X,Y,10);
```

The first column of XL1 = the first column of XL10 (x loading, 'P'), same for XS (x scores 'T'), and PCTVAR (% of variance explained by the model, which is presumably what is plotted in Figure 4). Of course, the regression coefficients differ, because a different number of components were used to build the model of Y, but this does not change the scores (or the % of variance explained by the first component).

In addition, there is a strong emphasis placed on the computed latent variables being "de-correlated". In my experience, when one computes PLS for a given factor, the first component will maximise the covariance between X and Y, but not 100%, meaning that subsequent shapes will also have some correlation with other Y - e.g. EDVi score has -0.75 correlation with EF, so this shape does not seem 'de-correlating' at all (if I understand what the authors mean by 'de-correlating'. In fact, usually ~10 components still capture some correlation with Y. Removing the first component that was computed to maximise covariance with e.g. EDV will remove some amount of EDV-related shape, but not ALL of it, which is what seems to be implied from the phrasing used in the manuscript. Therefore, despite the fact that the model with 10 latent variables yielded lower performance, it seems more "de-correlating" than the model with 1 latent variable, because the shape features related to the first variable have been more "completely" removed. However, my intuition is that removing the first 10 EDV-related shapes probably removes most of the variability of the shape from the population, since within those shapes there are some features that are also related to the other variables. So, I would think that a 1-component method is more suitable with this approach.

Regarding the comparison of methods and results, I don't find a convincing improvement of using PLS as opposed to PCA, in terms of accuracy or prediction. I do, however, agree that for interpretability of the results there is added gain of using this method. Therefore, I believe the idea of using PLS is valid, but the motivation for using it needs to be shifted in the paper.

Detailed comments:

Abstract:

- I am not convinced that a "novel method" is proposed, as stated in the abstract. Perhaps I have misunderstood the methods but they seem to be the same as previously proposed methods using the method described in [24] and applying to the data described and previously analysed in [13]. In my opinion, this work is the application of existing methods to a data-set and should be stated as such.

- What is meant by "a single PLS hidden variable"? I'm perhaps not familiar with this terminology, but is this referring to a single PLS latent variable or single PLS component?

- I also didn't exactly understand what is meant by a "decorrelation between scores". Is this referring to the orthogonalisation of the scores or reduction in the correlation of scores?

Introduction:

- Is there a difference between "LV volume index" and "LV volume", or is this referring to indexed LV volume? (line 55).

- It could be useful for the reader to define what is an orthogonal decomposition of shape (line 64).

- Line 79 - I think it may be more correct to state that PCA components are not designed to be related to clinical factors (though this can be the case). Clinical interpretation is not so much difficult, as it is suboptimal (in fact it is easy using PCR).

- Line 91 - as mentioned above, the term "PLS hidden variable" is unclear to me, could the authors clarify what exactly is meant by this (i.e. what is "hidden")?

- Last sentence page 4 - is this to say that there is no possible relationship between a clinical index and a previous shape? This phrasing "complete decorrelation" seems a bit strong to me.

Methods:

- General question: I'm curious to know why the authors didn't use the PLS regression coefficients directly since that is what PLS was mainly developed for (e.g. following the tutorial in Matlab on PLSR and PCR). Can the authors mention why they chose logistic regression instead? Was a comparison performed? Did it improve the results? Would we expect a logistic relationship over a linear one? Please clarify.

- General comment: It would be useful to clarify for the reader (especially those not familiar with latent variable models), what the component, loading, and scores are (i.e. component = loading x score)

- Line 103 - typo? should it be "heart failure or atrial fibrillation"?

- Line 112 - presumably Simpson's rule was applied? A citation here for clarity would be useful.

- Line 154 - perhaps deflation could be defined here. Deflation is typically used in original PLS algorithms but not SIMPLS, thus it could be nice to differentiate between standard 'deflation' and the orthogonalisation process used here

- N_latent was described before being introduced (page 6).

- I think the equation for maximising the covariance between T and U should be added here, and it should be mentioned that this constraint is what distinguishes PLS from, for example, PCA (i.e. this is how the shape modes are computed to maximise the variance in Y).

- The formula for B should be provided.

- Y_residuals is not defined.

- Line 153 - "this step ensures orthogonality" with respect to what? Presumably with respect to B but this is not explicitly stated.

- Line 162 - the term "PLS component" is introduced here to refer to the normalised regression coefficients B_i. Please consider another term to avoid confusion e.g. with 'component' as is used in PCA.

- Page 8 - why was 10 chosen as the upper limit for the number of latent variables?

- Page 8 - The authors claim that there is no standard method to choose the number of latent variables. Cross-validation could typically be used for this, as mentioned in the Matlab tutorial for PLSR and PCR. For such an investigation it would be nice to compute and plot the leave-one-out or split-half errors for the number of latent variables = 1:299 (number of subjects - 1), and then just the optimal errors could be reported.

- Line 172 - it could be useful to mention why X^k+1 is orthogonal to B^k.

- Line 183 - details on the logistic regression technique and how this was performed could be added (stepwise forward logistic regression? SPSS?).

- Line 186 - BMI and SBP should be defined here.

- Line 187 - it would be nice to mention why these were chosen as the baseline variables and why baseline variables were included.

- Line 188 - Why was a 6 component PCA model used? According to [13] this model only represents ~75% of the shape variance in the population.

- Line 202 - is ESV used without indexing? If not, LVESVi should be used. If yes, why was EDV indexed and not ESV?

Analyses

- Line 199 - Please add the statistical significance threshold ($p < 0.05$), or to avoid repetition, just state once at the beginning of this section that statistical significance was set at $p<0.05$.

- For reproducibility purposes it could help the reader to mention which software (if any) was used to perform the statistical analyses

- Line 222 - could the authors elaborate on this sentence, I didn't get what is meant by 'retaining correlation with the index', and why this would be a bad thing

- Line 226 - I am very surprised to see that only 15% of the shape variance in the population was captured by 6 components from the N=10 model. Again, perhaps I have misunderstood, but my understanding based on the description of the methods is that the 10-component model should have 10-components for EDV, 10 for sphericity, and so on, so there should actually be 10 x 6 = 60 components for this model, and therefore I would expect a much larger amount of the variance to be captured in such a model. Could the authors clarify why this is not the case, or please correct me if I am wrong about the methods.

- Line 246 - presumably 'LR' stands for logistic regression? Could you add this to the text and figures

- Line 246 - why was the median chosen? Please mention briefly here.

- Line 250 - how are the baseline variables adjusted? Does this significant change the shapes? (This question is more out of curiosity than actually needing clarification)

Discussion

- Line 266 - as mentioned previously, I would rather state that an orthogonal PLS framework was applied, without implying that there are new methods proposed in the present work. Again, if this is not the case, please clearly describe the contributions of the present work and distinguish how this method differs from other orthogonal PLS methods.

- Line 273 - orthogonality was described here, but should also be mentioned at the beginning of the methods section.

- Line 274 - I got a bit lost here with the terminology, are the "PLS shape components" referring to loading x score or are you referring to the loading (which I guess is the case because PLS loadings are orthogonal)? And presumably "PLS shape component score" is referring just to the scores (which are not necessarily orthogonal for PLS)? Here there is also the mention of the term 'decorrelated', should that be 'orthogonal'?

- Line 284 - there is again the use of 'decorrelation' and I just now think I understand what is meant by this. Perhaps "reduction/decrease in correlation" is clearer?

- Line 285 - I'm honestly very surprised to see "total decorrelation" (and again, I would suggest using "zero correlation" rather than "decorrelation") between the PLS scores and clinical indices. Indeed this suggests that the 1-component model is able to remove any relationship with EDVi (for the second component), and so on.

Results

- In all results (and tables, figures) it would be useful to clarify when experiments are including both populations and when it is MI only, sometimes I got confused by that.

- I'm not sure how to interpret the results. Are the authors looking for the most predictive model? In that case I would expect to see a more thorough analysis of the number of latent variables (using cross-validation).

- Do the authors have some reasoning for why LS score was significant with the 1-component model and not the 10-component model, and vice versa for conicity?

Tables

- In all tables it would be useful to include the abbreviations

- The tables are in general very content-heavy, and it's not easy to see what the take-home message is from each table. Some additional annotations or descriptions in the legend would help guide the reader to interpret these results. For example, the statistically significant components in Table 8 could be highlighted for easy readability, rather than using an asterix.

- Are Tables 2-7 showing results for the MI population only or are these combined results for both populations (please specify in the legends).

- In Table 8 it would be useful to include some descriptions of what are "good" values in terms of the coefficient, error, OR and CI.

- Table 9 is a nice summary of the results and easy to interpret. Line 195 could be repeated here to remind the reader what is preferred for each measure (e.g. >AIC = better)

- Table 5 and 6 - it is not clear what is meant by 'PLS clinical mode scores' and how this is different from 'PLS component scores'.

- Table 7 should be moved to follow Tables 2,3 for easier readability.

Figures

- In all figures it would be helpful to include the abbreviations

- Figure 1 is nice and clear. If possible, it could be useful to include on the left-hand side an image depicting each measure or the formula for computing each measure, and on the right-hand side the corresponding modes at +1SD. X6 could be pointing downwards for consistency

- I don't find Figure 2 and Figure 3 very informative in the sense that I don't know what I should conclude from these images. Perhaps some annotation could help as guidance.

- It would be nice to have some interpretation and comparison of the modes in Figure 2 and 3 to the modes in Figure 14 of [13] in terms of highlighting for the reader regions of interest or interesting behaviour that is visible from these modes (i.e. what should we, as readers, take from these Figures?)

- The labels on the x-axis of Fig. 4 are a bit misleading. I would rather put 'PC1' directly below the blue column, and EDVI PC below the red/green columns (since PC1 in PCA is not related to EDVI, or am I mistaken?).

- Figure 4 - I am very confused by these results, especially for the first component. To my understanding, in both the 1-component and 10-component models, PLS was performed with the same X shape features and EDVI as the Y variable. There is no tuning of SIMPLS to force all of the variance to be in the N-components, therefore the variance of the first component should be equal, regardless of the number of components that was chosen. The number of chosen components changes the accuracy of the regression, but not the components themselves. Therefore, the variance of the first component should be much higher than what is reported for the 10-component model. While there would be large differences in the subsequent components (because there is much fewer variance in the other components for the 10-component model because so much of the shape has already been removed from X^k), the first component should be identical to the 1-component model (i.e. 50%). Please clarify why this is not the case.

- Figure 5 - The improvement from baseline alone is clear (and expected) but I don't see a dramatic improvement based on the figures for the shape-based models and using clinical indices alone. Moreover, there isn't a clear improvement above PCA.

- Figure 5- could the authors add AUC (as reported on line 239) to the figure?

- Figure 6 is interesting. Perhaps the author could consider adding some annotation to guide the reader about the shape differences (e.g. there seems to be less systolic contraction in the MI patients) and a summary of what to conclude in the legend (even a repetition of line 249 would be helpful here).

Tools:

- For the sake of this journal (being focused towards open-source tools), I would suggest that the authors use R (https://cran.r-project.org/web/packages/pls/index.html) instead of Matlab, to avoid the need for users to purchase a Matlab license. Using the plsregress function also requires a license for the Statistics and Machine Learning Toolbox.

- I am not familiar with Giga science, but based on the website it is stated that all research objects are published (data, software tools, and workflows). In order to reproduce the results from this study (or indeed to apply the methods to new data), the community would need to have access to the image processing tools that were used to extract the models. PCA (or similarly PLS) applied to data that has already been extracted and parameterised is straightforward using existing software (or indeed using built-in Matlab, python, or R functions). While it is a useful resource to have access to the images and the models extracted from these images, the biggest challenge we face in the field is in creating the models to be able to perform the analysis.

Novelty:

- As mentioned previously, to my knowledge this technique has already been described in [24] and there is inadequate referencing to previous techniques. Orthogonalisation using the Gram-Schmidt method has been discussed earlier, for example Izenman, A.J., 2008. Modern multivariate statistical techniques (Vol. 1). New York: Springer, page 570), and for PLS specifically: de Jong, S., Wise, B.M. and Ricker, N.L., 2001. Canonical partial least squares and continuum power regression. Journal of Chemometrics, 15(2), pp.85-100. Moreover, the Matlab code for canonical (i.e. orthogonal) SIMPLS is provided in this paper.

Code:

- It isn't clear to me why the regression coefficients ('Beta') are normalised in 'GenerateOrthogonalModes' and subsequently why the scores and loadings from the plsregress function are not used directly. Could the authors explicitly mention why this normalisation is important.

- As mentioned previously, from what I understand from the code, the 'pc_scores' are computed as X*B (data matrix X times the regression coefficients). However, this is the model of Y, not the computation of the scores. The scores T would usually be computed by projecting X onto the loadings P.

Methods

Are the methods appropriate to the aims of the study, are they well described, and are necessary controls included? No

Conclusions

Are the conclusions adequately supported by the data shown? No

Reporting Standards

Does the manuscript adhere to the journal's guidelines on minimum standards of reporting? Yes

Statistics

Are you able to assess all statistics in the manuscript, including the appropriateness of statistical tests used? Yes, and I have assessed the statistics in my report.

Quality of Written English

Please indicate the quality of language in the manuscript: Acceptable

Declaration of Competing Interests

Please complete a declaration of competing interests, considering the following questions:

- Have you in the past five years received reimbursements, fees, funding, or salary from an organisation that may in any way gain or lose financially from the publication of this manuscript, either now or in the future?
- Do you hold any stocks or shares in an organisation that may in any way gain or lose financially from the publication of this manuscript, either now or in the future?
- Do you hold or are you currently applying for any patents relating to the content of the manuscript?
- Have you received reimbursements, fees, funding, or salary from an organization that holds or has applied for patents relating to the content of the manuscript?
- Do you have any other financial competing interests?
- Do you have any non-financial competing interests in relation to this paper?

If you can answer no to all of the above, write 'I declare that I have no competing interests' below. If your reply is yes to any, please give details below.

I declare that I have no competing interests

I agree to the open peer review policy of the journal. I understand that my name will be included on my report to the authors and, if the manuscript is accepted for publication, my named report including any attachments I upload will be posted on the website along with the authors' responses. I agree for my report to be made available under an Open Access Creative Commons CC-BY license (http://creativecommons.org/licenses/by/4.0/). I understand that any comments which I do not wish to

be included in my named report can be included as confidential comments to the editors, which will not be published.

I agree to the open peer review policy of the journal

To further support our reviewers, we have joined with Publons, where you can gain additional credit to further highlight your hard work (see: https://publons.com/journal/530/gigascience). On publication of this paper, your review will be automatically added to Publons, you can then choose whether or not to claim your Publons credit. I understand this statement.

Yes
