# Supplementary material for: Orthogonal decomposition of left ventricular remodeling in myocardial infarction
Source: Gigascience. 2017 Feb 6;6(3):1–15. doi: 10.1093/gigascience/gix005 (PMC5791439; doi:10.1093/gigascience/gix005)
Supplement: Reviewer_2_Report_(Original_Submission).pdf [file gix005_reviewer_2_report_(original_submission).pdf]

Reviewer Report

Title: "Orthogonal Decomposition of Left Ventricular Remodelling in Myocardial Infarction"

Version: Original Submission **Date:** 9/12/2016

Reviewer name: Einar Heiberg

Reviewer Comments to Author:

GENERAL COMMENTS

Remodeling of the left ventricle as a response to disease is a complex and multifactorial process. Understanding the remodeling process and being able to predict who will remodel is great clinical interested, especially as heart failure is growing and is soon the biggest cause of death world-wide.

Traditionally this has been addressed in crude clinical indices and in longitudinal studies to see how they can predict remodeling and adverse events.

This paper and the field would benefit from going beyond both simple crude clinical measurements towards computational models incorporating both patient specific geometrical and functional data, as well as tissue characteristics such as for instance as infarct location, and infarct transmurality which is ignored in this paper.

The strength of this paper is the novel mathematical process of making decoupled geometrical modes, while still correlating with clinical indices, and the main limitations is that the study is cross sectional and us such limited understanding can be gained on what really drives the remodeling. The paper is missing a limitation section where the lack of cross sectional data is highlighted and the need for such future research is discussed.

The paper derives novel shape parameters correlating with known classical clinical indices for remodeling and their only validation presented is their ability to determine whether a subject has had an infarction or not.

Given major shortcoming of the lack of longitudinal data the paper still has merits enough warranting publication in terms of providing a general framework to construct linear and orthogonal shape decompositions that correlates with (rather) arbitrary clinical indices. This will give better understanding on shape compositions and make them less of a black box magic to clinicians.

DETAILED COMMENTS

1. Abstract: A novel method for deriving orthogonal shape components directly from any set of clinical indices. The word any is a strong word given the mathematical depth of the paper. For instance,

the clinical indices need to be reasonably well uncorrelated for the operation to be meaningful and produce shape components that do correlate with the chosen clinical indices.

2. Abstract. Why is not infarct size one of the clinical indices? Likely, it must be stronger than for instance longitudinal shortening to determine remodeling?

3. Page 3, Line 67. In this section you may lose some of the potential readers of the paper. I do understand and acknowledge that it greatly simplifies that any given metric tensor does not have off diagonal elements and is orthonormal ideally. However, is this really a practical limitation as in order to compute measures such as arc lengths and areas it simple to reconstruct the original shape of the patient and compute them directly in the Euclidean space? Well it is more computationally intensive, but it is more convenience rather than anything else?

4. Page 5, line 114. The definition of relative wall thickness is rather strange, I presume that this is the form where previous researchers got significant correlations for prognostics for this parameter, but it would be good to have some more rationale on why this rather bizarre formulation, and why not for instance absolute mean wall thickness in mm (or even minimal wall thickness from thinned after myocardial infarction etc).

5. Page 6, line 119. Some more details would be good here. Is it the basal AV plane movement divided by the straight distance to apex or is it divided by the curve length? Central basal point is this the middle point of the mitral valve?

6. Page 7, Line 156. How was the next component to be removed from the shape space determined? Greatest variance in what respect?

7. Page 11, line 252. This paragraph is somewhat important as I understand it in terms of possible application of the technology. This section could perhaps be better explained and expanded as it deals with how the shape decomposition can be used to derive new knowledge.

Please when introducing new abbreviations such as LR help write them out in the text. Is it correct that LR in this context it is logistic regression?

8. Page 14, line 306. The concept of tracking patients over time with shape decompositions should be highlighted better as this is a rather new concept at least to clinicians and how then such changes can be better understood given orthogonal bases. Please expand somewhat if possible.

9. Page 14, potential implications. Myocardial infarction is a rather broad category in terms of location, and transmurality of the infarct. Furthermore, nowhere throughout the paper it is discussed other causes of remodeling such as valvular disease. As I understood from the description of the normals they did not have valvular disease, but it is rather likely that the infarct patients had such comorbidities.

10. Page 21. Table 1. What is the "old" of the myocardial infarction, i.e how long was it between myocardial infarction and imaging. This may be highly important since that if all are fresh infarction s(< months), then rather little remodeling may have occurred such as limited wall thinning in the infarcted area etc. It is very acute then you have myocardial edema etc as well.

11. Page 22, Table 2, it is maybe worth commenting on in the text that LS and RWT achieves rather low correlations compared to their clinical indices. This is even visible in Figure 2, where the 90th percentile of LS does not really show much influence on longitudinal shortening. In fact, as I understand it as the correlation is about 0.5, then this shape mode do only explain 25% (0.5*0.5=0.25) of longitudinal shortening is this correct? How meaningful are really correlations below say 0.7(=> 50% explaining power)?

12. Page 25, Table 8. What is meant with the baseline model? I find the baseline model poorly described in the paper, please provide more details.

13. Figure 1. Is the order of the indices a design choice or is it based on data? Please expand the legend. See also comment 6.

14. Is it not strange that given the order EDVI, Sphericity, EF, RWT, Conicity, LS index that the correlations in Table 2 are not dropping in that order, or is this not necessary and rather reflects underlying correlation (or lack thereof) of the clinical indices. If possible, please expand on this.

15. Figure 4, is it possible to choice grayscale or colors that works when printed on a grey scale printer.

16. Figure 5, the legend does not describe what is really tested (the decompositions) power to tell if a given patient has in infarction or not? Correct? What is here meant with baseline?

17. What is the stability of the suggest method? You used the SIMPLS algorithm as implemented by Mathworks, would this change with another algorithm? Are there fundamental differences in possible solutions? Either perform some experiments or discuss this theoretically.

The other factor that would influence the choice of subject population. Here you have 300 infarct patients and some 2000 "normals". Would you get to the same decomposition if you used another set of infarct patients and normal as well as another ratio between normals and patients? This could be tested by taking a sub-population of the input data and perform the computations and compare how these two decompositions coincide in some suitable measure. This would significantly strengthen the paper as the paper describes a rather generic approach to shape decompositions.

Methods

Are the methods appropriate to the aims of the study, are they well described, and are necessary controls included? Yes

Conclusions

Are the conclusions adequately supported by the data shown? Yes

Reporting Standards

Does the manuscript adhere to the journal's guidelines on minimum standards of reporting? Yes

Statistics

Are you able to assess all statistics in the manuscript, including the appropriateness of statistical tests used? No, and I do not feel adequately qualified to assess the statistics.

Quality of Written English

Please indicate the quality of language in the manuscript: Acceptable

Declaration of Competing Interests

Please complete a declaration of competing interests, considering the following questions:

- Have you in the past five years received reimbursements, fees, funding, or salary from an organisation that may in any way gain or lose financially from the publication of this manuscript, either now or in the future?
- Do you hold any stocks or shares in an organisation that may in any way gain or lose financially from the publication of this manuscript, either now or in the future?
- Do you hold or are you currently applying for any patents relating to the content of the manuscript?
- Have you received reimbursements, fees, funding, or salary from an organization that holds or has applied for patents relating to the content of the manuscript?
- Do you have any other financial competing interests?
- Do you have any non-financial competing interests in relation to this paper?

If you can answer no to all of the above, write 'I declare that I have no competing interests' below. If your reply is yes to any, please give details below.

Founder and major owner of Medviso AB, a company that produce cardiovascular image analysis software.

I agree to the open peer review policy of the journal. I understand that my name will be included on my report to the authors and, if the manuscript is accepted for publication, my named report including any attachments I upload will be posted on the website along with the authors' responses. I agree for my report to be made available under an Open Access Creative Commons CC-BY license (http://creativecommons.org/licenses/by/4.0/). I understand that any comments which I do not wish to be included in my named report can be included as confidential comments to the editors, which will not be published.

I agree to the open peer review policy of the journal

To further support our reviewers, we have joined with Publons, where you can gain additional credit to further highlight your hard work (see: https://publons.com/journal/530/gigascience). On publication of this paper, your review will be automatically added to Publons, you can then choose whether or not to claim your Publons credit. I understand this statement.

Yes
