# Supplementary material for: Orthogonal decomposition of left ventricular remodeling in myocardial infarction
Source: Gigascience. 2017 Feb 6;6(3):1–15. doi: 10.1093/gigascience/gix005 (PMC5791439; doi:10.1093/gigascience/gix005)
Supplement: Reviewer_3_Report_(Original_Submission).pdf [file gix005_reviewer_3_report_(original_submission).pdf]

Reviewer Report

Title: "Orthogonal Decomposition of Left Ventricular Remodelling in Myocardial Infarction"

Version: Original Submission **Date:** 10/14/2016

Reviewer name: Karim Lekadir

Reviewer Comments to Author:

This paper presents an approach to extract new shape indices from asymptomatic and infarcted ventricles such that they are orthogonal and have high prediction capability. The paper is well written and can be of interest to the statistical cardiac modeling community.

Comments/Questions:

1) PLS has already been used for myocardial infarction classification by Lekadir et al. in STACOM 2015. The authors should cite this paper and describe the differences between the two works.

2) What is the difference between calculating the PLS indices based on the clinical indices (EDVI, sphericity, etc) instead of directly using the class labels (asymptomatic vs. Infarcted) as in Lekadir et al. STACOM 2015? The authors should compare the extracted PLS scores through the two methods and see if there are indeed differences.

3) The authors used an imbalance dataset to train the PLS models (300 abnormal vs. about 2000 healthy cases), which may affect the significance of the new shape indices. It would be good to verify if data imbalance affects or not the extraction of the new shape indices. I suggest that the authors run the same experiments with the 300 infarcted cases and 300 randomly selected asymptomatic cases and compare the results.

4) It would have been interesting to have a method that finds automatically the best order in the calculation of the PLS score, may be using some statistical criteria, instead of the ad hoc order used in the manuscript (i.e. EDVI, sphericity, EF, etc). What happens if you start with wall thickness for example, which is more directly linked to myocardial infarction?

5) What is the clinical meaning of the extracted PLS indices? How can they be used by clinicians? Can you show some figures illustrating the variation induced by these indices and their clinical meaning? How do these indices describe better remodeling or infarction than standard clinical indices?

Methods

Are the methods appropriate to the aims of the study, are they well described, and are necessary controls included? Yes

Conclusions

Are the conclusions adequately supported by the data shown? Yes

Reporting Standards

Does the manuscript adhere to the journal's guidelines on minimum standards of reporting? Yes

Statistics

Are you able to assess all statistics in the manuscript, including the appropriateness of statistical tests used? Yes, and I have assessed the statistics in my report.

Quality of Written English

Please indicate the quality of language in the manuscript: Choose an item.

Declaration of Competing Interests

Please complete a declaration of competing interests, considering the following questions:

- Have you in the past five years received reimbursements, fees, funding, or salary from an organisation that may in any way gain or lose financially from the publication of this manuscript, either now or in the future?
- Do you hold any stocks or shares in an organisation that may in any way gain or lose financially from the publication of this manuscript, either now or in the future?
- Do you hold or are you currently applying for any patents relating to the content of the manuscript?
- Have you received reimbursements, fees, funding, or salary from an organization that holds or has applied for patents relating to the content of the manuscript?
- Do you have any other financial competing interests?
- Do you have any non-financial competing interests in relation to this paper?

If you can answer no to all of the above, write 'I declare that I have no competing interests' below. If your reply is yes to any, please give details below.

I declare that I have no competing interests

I agree to the open peer review policy of the journal. I understand that my name will be included on my report to the authors and, if the manuscript is accepted for publication, my named report including any attachments I upload will be posted on the website along with the authors' responses. I agree for my report to be made available under an Open Access Creative Commons CC-BY license (http://creativecommons.org/licenses/by/4.0/). I understand that any comments which I do not wish to be included in my named report can be included as confidential comments to the editors, which will not be published.

I agree to the open peer review policy of the journal

To further support our reviewers, we have joined with Publons, where you can gain additional credit to further highlight your hard work (see: https://publons.com/journal/530/gigascience). On publication of this paper, your review will be automatically added to Publons, you can then choose whether or not to claim your Publons credit. I understand this statement.

Yes
